# Prescription Opioid Use and Risk for Major Depressive Disorder and Anxiety and Stress-Related Disorders: A Multivariable Mendelian Randomization Analysis

**DOI:** 10.1001/jamapsychiatry.2020.3554

**Published:** 2020-11-11

**Authors:** Daniel B. Rosoff, George Davey Smith, Falk W. Lohoff

**Affiliations:** 1 Section on Clinical Genomics and Experimental Therapeutics, National Institute on Alcohol Abuse and Alcoholism, National Institutes of Health, Bethesda, Maryland; 2 MRC Integrative Epidemiology Unit at the University of Bristol, Bristol, England

## Abstract

**Importance:**

Growing evidence suggests that prescription opioid use affects depression and anxiety disorders; however, observational studies are subject to confounding, making causal inference and determining the direction of these associations difficult.

**Objective:**

To investigate the potential bidirectional associations between the genetic liability for prescription opioid and other nonopioid pain medications and both major depressive disorder (MDD) and anxiety and stress-related disorders (ASRD) using genetically based methods.

**Design, Setting, and Participants:**

We performed 2-sample mendelian randomization (MR) using summary statistics from genome-wide association studies (GWAS) to assess potential associations of self-reported prescription opioid and nonopioid analgesics, including nonsteroidal anti-inflammatories (NSAIDs) and acetaminophen-like derivatives use with MDD and ASRD. The GWAS data were derived from participants of predominantly European ancestry included in observational cohorts. Data were analyzed February 20, 2020, to May 4, 2020.

**Main Outcomes and Measures:**

Major depressive disorder, ASRD, and self-reported pain medications (opioids, NSAIDs, anilides, and salicylic acid).

**Results:**

The GWAS data were derived from participants of predominantly European ancestry included in the population-based UK Biobank and Lundbeck Foundation Initiative for Integrative Psychiatric Research studies: approximately 54% of the initial UK Biobank sample and 55.6% of the Lundbeck Foundation Initiative for Integrative Psychiatric Research sample selected for the ASRD GWAS were women. In a combined sample size of 737 473 study participants, single-variable MR showed that genetic liability for increased prescription opioid use was associated with increased risk of both MDD (odds ratio [OR] per unit increase in log odds opioid use, 1.14; 95% CI, 1.06-1.22; *P* < .001) and ASRD (OR, 1.24; 95% CI, 1.07-1.44; *P* = .004). Using multivariable MR, these opioid use estimates remained after accounting for other nonopioid pain medications (MDD OR, 1.14; 95% CI, 1.04-1.25; *P* = .005; ASRD OR, 1.30; 95% CI, 1.08-1.46; *P* = .006), and in separate models, accounting for comorbid pain conditions. Bidirectional analyses showed that genetic liability for MDD but not ASRD was associated with increased prescription opioid use risk (OR, 1.18; 95% CI, 1.08-1.30; *P* < .001). These estimates were generally consistent across single-variable and multivariable inverse variance–weighted (MV-IVW) and MR-Egger sensitivity analyses. Pleiotropy-robust methods did not indicate bias in any MV-IVW estimates.

**Conclusions and Relevance:**

The findings of this mendelian randomization analysis suggest evidence for potential causal associations between the genetic liability for increased prescription opioid use and the risk for MDD and ASRD. While replication studies are necessary, these findings may inform prevention and intervention strategies directed toward the opioid epidemic and depression.

## Key Points

**Question:**

Does prescription opioid medication have a potentially causal role in the risk for depression and anxiety disorder?

**Findings:**

In this 2-sample mendelian randomization study using genetic instruments for common pain medications, the genetic liability for prescription opioid use was associated with increased risk for major depression.

**Meaning:**

While further work is needed, this genetics-based study supports conventional observational literature suggesting prescription opioid use increases the risk for depression.

## Teaser

This mendelian randomization analysis investigates the potential bidirectional associations between the genetic liability for prescription opioid and other nonopioid pain medications and both major depressive disorder and anxiety and stress-related disorders using genetically based methods

## Introduction

The United States is in the middle of an opioid epidemic,^1^ with an approximately 5-fold increase in opioid prescription use over the past 20 years resulting in large increases in opioid misuse.^2^ Opioid-related deaths are rapidly increasing, and approximately 68% of the 702 000 drug overdose deaths in the United States from 2013 to 2017 involved opioids.^3,4^ It is also reported that prescription opioids (vs illicit opioids) are the first opioids to be misused,^5^ with almost 30% of patients prescribed opioids for chronic pain misusing them^6^ and about 12% developing opioid use disorder (OUD).^6^

Informed prescribing practices require comprehensive understanding of the treatment’s risks and benefits, and while opioids alleviate pain, chronic use is associated with numerous adverse effects, including immunosuppression, natural reward processes dysregulation, and neurohormonal deficits.^7,8^ Observational studies have also found opioid use to be highly comorbid with both major depressive disorder (MDD) and anxiety and stress-related disorders (ASRD).^9,10,11,12^ It is estimated that the approximately 7.8 million adults with psychiatric disorders (primarily MDD and ASRD) receive more than half of the almost 200 million yearly opioid prescriptions.^10^ Compared with other patient populations, individuals with psychiatric disorders are also more likely to report long-term opioid use.^13,14,15^

Because MDD and ASRD are leading global causes of disability and death,^16,17^ elucidating the direction and potentially causal effect of these associations would be useful to inform prevention strategies. Literature suggests prescription opioid use increases MDD risk,^18,19,20,21^ and while observational findings suggest a potential association between prescription opioid use, MDD, and ASRD, observational data are subject to confounding and reverse causation, making causal inference difficult.^22,23^ Therefore it remains to be elucidated whether MDD is a cause or consequence of prescription opioid use.^18^ While prospective randomized clinical trials (RCTs) are the criterion standard of causal inference,^24^ performing RCTs to evaluate the effects of prescription opioid use or other nonopioid pain medications is often complicated by preexisting psychiatric comorbidities.

Mendelian randomization (MR), which uses single-nucleotide variants (SNVs) as unconfounded proxies for exposures to estimate their effect on outcomes of interest, minimizes the bias affecting observational epidemiologic studies.^23,25,26,27^ Conceptually, MR has analogies with RCTs, with randomization occurring at meiosis,^23,26^ and is an important strategy for strengthening causal inference when RCTs are impractical or unethical.^23^ Given the public health importance of assessing the possible bidirectional associations between prescription opioid use and neuropsychiatric disorders, gaining causal inference into these associations would be important to aid prevention strategies.

In the absence of RCTs, we used a 2-sample MR study design of summary-level data on self-reported prescription opioid use in the UK Biobank (UKB),^28^ the largest genome-wide association study (GWAS) to date on MDD^29^ and the largest GWAS to date on ASRD,^30^ to conduct bidirectional MR analyses investigating potential causal associations between the genetic liability for opioid pain medications and MDD and ASRD. Given prescription opioid medications are given for pain, and pain increases the risk for both MDD and ASRD,^31,32^ we aimed to provide context to our primary prescription opioid analysis by including other common pain medications, including anilides, salicylic acid and derivatives, and nonsteroidal anti-inflammatory drugs (NSAIDs) and chronic pain conditions to the study. We also leveraged multivariable MR (MVMR) methods developed in 2019^33^ to account for potential confounding owing to these pain medications and chronic pain conditions that may affect the use of prescription opioid analgesics and the risk for these psychiatric disorders.

## Methods

### Study Design and Data Sources

**Figure 1.**
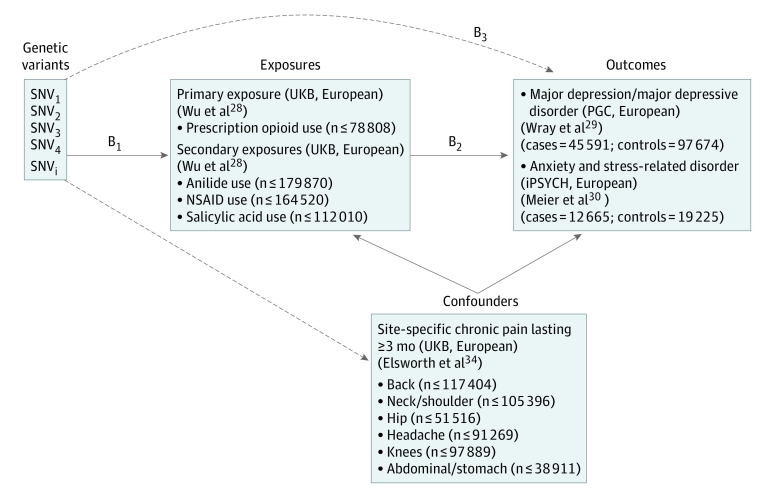
Study Overview and Mendelian Randomization (MR) Model All summary-level genetic associations were derived from cohorts of European ancestry. Consortium, study cohort, and author information of original genome-wide association study for each exposure, confounder, and outcome included in the study are in parentheses. B_2_ is the association of interest (prescription opioid use on major depressive disorder [MDD] and anxiety and stress-related disorder [ASRD] risk), estimated by B_2_ = B_1_ / B_3_. B_1_ and B_3_ are the estimated direct association of the genetic variants on the exposure (ie, prescription opioid use) and the outcomes (ie, MDD and ASRD), respectively. iPSYCH indicates The Lundbeck Foundation Initiative for Integrative Psychiatric Research; IVW, inverse variance–weighted MR; MRC-IEU, Medical Research Center-Integrative Epidemiology Center (UK Bristol); NSAID, nonsteroidal anti-inflammatory or antirheumatic drugs; PGC, Psychiatric Genomics Consortium; SNV, single-nucleotide variant; UKB, UK Biobank.

A detailed description of the methods used in this study is provided in the eMethods in the Supplement. We used publicly available summary statistics from 3 GWAS sources of predominantly European ancestry (Figure 1; eTable 1 in the Supplement). All studies have existing ethical permissions from their respective institutional review boards and include participant written informed consent and rigorous quality control. Because all analyses herein are based on publicly available summary data, no ethical approval from an institutional review board was required for this study. Data for this study were analyzed from February 20, 2020, to May 4, 2020.

### Data Sets

We used summary statistics from the first medication use case-control GWAS conducted among UKB study participants to generate genetic instruments for opioid and nonopioid pain medications.^28^ Pain medication categories were classified by active ingredient using the Anatomical Therapeutic Chemical Classification System and then assigned to 23 categories by active ingredient, including opioids (eg, morphine, oxycodone, codeine, fentanyl, pethidine, and tramadol), NSAIDs, anilides, and salicylic acid products (eTable 2 in the Supplement). Approximately 54% of UKB study participants are women, and the mean (SD) age of UKB study participants on the first visit UKB assessment was 56.5 (8.1) years.^28^ We also used summary statistics from MRC-IEU UKB GWASs^34^ for 6 PHEnome Scan Analysis Tool (PHESANT)^35^ phenotypes related to site-specific (back, knee, hip, neck/shoulder, headache, and abdominal/stomach) pain occurring for more than 3 months.

We used summary statistics from the largest publicly available GWASs for our MDD and ASRD gene associations.^29,30^ The MDD cases were required to meet international consensus criteria (*DSM-IV*, *International Classification of Diseases, Ninth Revision*, or *International Statistical Classification of Diseases and Related Health Problems, Tenth Revision*) for a lifetime diagnosis of MDD (45 591 cases and 97 674 controls).^29^ The MDD GWAS meta-analysis^29^ incorporated 29 cohorts. Cohorts had a wide range of ages (18 to >80 years), and approximately 56.0% of the participants were women.^29^ The ASRD cases and controls (12 665 cases and 19 220 controls) were selected from the Danish Lundbeck Foundation Initiative for Integrative Psychiatric Research (iPSYCH) cohort, a representative sample from the population of Denmark born between May 1, 1981, and December 31, 2005.^30^ Approximately 55.6% of ASRD sample participants were women. The ASRD cases were assigned by a psychiatrist during routine clinical care according to the international *Statistical Classification of Diseases and Related Health Problems (10th Revision)*.^30^ Both MDD and ASRD cohort participants were of European ancestry, and all GWASs included age and sex as covariates in the association analysis; population stratification is taken into account in the principal components analysis.^28,29,30^

### Sample Independence

Participant overlap in samples used to estimate genetic associations between exposures and outcomes can increase weak instrument bias in MR analyses.^36,37^ We avoided overlap in our analyses of pain medication use on MDD and ASRD: we used meta-analytic results for MDD that excluded the UKB cohort (n = 29 740). To improve precision for the bidirectional analyses of MDD on pain medication use, we used meta-analytic results for MDD including additional cohorts from 23andMe (n = 307 354) and UKB (overall n = 480 359). Hence, sample overlap (for bidirectional analyses) was minimal (6.2%), and because MDD instrument strength was considered strong (*F *statistic of approximately 32, see subsequent text), considerable weak instrument bias is not expected.^37^

### Statistical Analysis

All analyses were performed using the TwoSampleMR and MendelianRandomization R packages (The R Foundation). We selected all relevant SNVs identified in each GWASs as having reached selection threshold *P* less than  5 × 10^−6^ and being uncorrelated (10 000 kilobase pairs apart and *R^2^* ≤.001). We obtained SNV effects and corresponding standard errors from the exposure and outcome GWASs.^38^ We harmonized exposure and outcome data, removed palindromic SNVs with intermediate allele frequencies, and estimated the *F* parameter to evaluate instrument strength.^37^ We applied Steiger filtering to the harmonized data to identify and remove those SNVs exhibiting reverse causation by the test metric. The observed variance of the outcome exceeded the observed variance of the exposure explained by the SNVs (eTables 3-8 in the Supplement: harmonized data sets).^39^

For single-variable MR analysis, we used inverse variance–weighted (IVW) MR as the primary method. However, because this method gives consistent estimates only if all genetic variants are valid instrumental variables (IVs), we considered complementary MR-Egger and weighted median-based regression methods, which make different IV assumptions, as a sensitivity analysis to address the question of robustness of our IVW estimate.^23,38,40^ The MR-Egger regression gives consistent estimates when 100% of genetic variants are invalid IVs; weighted median requires 50% of the weight to come from valid IVs. However, regarding efficiency, weighted median estimates generally are nearly as precise as IVW estimates; both are substantially more precise than MR-Egger estimates, with MR-Egger regression estimates particularly imprecise if all IVs have similar magnitudes of association with the exposure.^41^ We used the MR-Egger intercept test,^42^ Cochran *Q* heterogeneity test,^43^ and MR pleiotropy residual sum and outlier (MR-PRESSO) test^44^ to evaluate potential IV violations. We also performed leave-one-out analyses to detect high influence points.^39^

For MVMR analyses, we constructed instruments using SNVs in each of the GWASs meeting our single-variable MR selection criteria, described previously. We used the MVMR extension of the inverse-variance weighted MR method^36^ and MR-Egger method to correct for both measured and unmeasured pleiotropy.^45^ We combined the SNVs from the relevant GWASs: prescription opioid plus nonprescription pain medications; prescription opioid plus each of the chronic pain conditions; and the bidirectional MDD plus ASRD instrument. We removed duplicate and correlated SNVs (within 10 000 kilobase pairs; *R^2^* ≥0.001), then extracted the SNV effects and corresponding standard errors from the exposures and outcome GWASs (eTables 9-14 in the Supplement: harmonized data sets).

The MR estimates are reported as odds ratios (ORs) interpreted as MDD or ASRD risk per unit increase in log odds of the opioid or nonopioid medication use (or alternatively, for bidirectional results, the medication use risk per unit increase in log odds of MDD or ASRD). We report confidence intervals for the main results. We index the strength of evidence against the null hypotheses (no association) by the exact *P* value. While we caution against interpreting study findings solely on the basis of a *P*-value threshold,^46^ we use a 2-sided α of .00625 based on testing 4 pain medications categories against 2 psychiatric outcomes as a heuristic allowing for follow-up of a plausible number of findings. In assessing consistency and robustness, we looked for estimates substantially agreeing in direction and magnitude (overlapping confidence intervals) across complementary MR methods.

## Results

**Figure 2.**
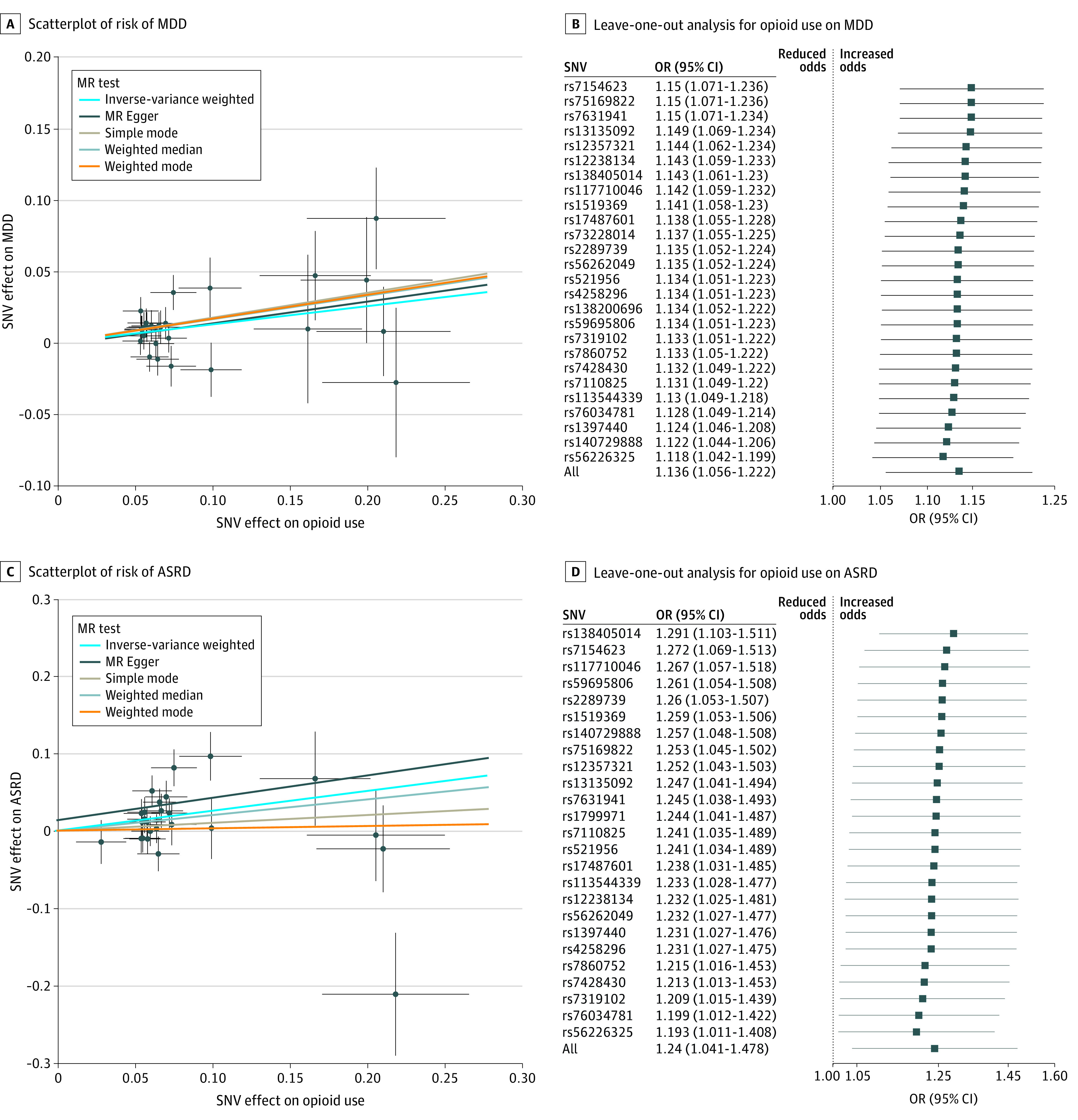
Scatterplot and Leave-One-Out Analysis of Associations of Genetic Risk of Prescription Opioid Use on Risk of MDD (A and B) and ASRD (C and D) Scatterplot of independent instrument single-nucleotide variant (SNV) exposure effects vs outcome effects from 2 independent samples augmented by the standard error of these effects on the vertical and horizontal sides (for presentation, alleles are coded so that all SNV exposure effects are positive). Solid lines are the regression slopes fitted by the primary inverse variance–weighted (IVW) and complementary mendelian randomization (MR) methods: slopes fitted by IVW MR method were very similar in direction and magnitude to slopes fitted by MR-Egger and weighted median methods for both risk of major depressive disorder (MDD) (A) and risk of anxiety and stress-related disorders (ASRD) (C). In leave-one-out sensitivity analyses, IVW MR was performed leaving out each SNV in turn to identify whether a single SNV may be driving the association, with results illustrated in plots showing that no single SNV was driving the association between genetic risk of prescription opioid use and MDD (B) or ASRD (D), respectively: the relevant comparisons would be between the overall IVW MR interval estimate ("ALL") vs each leave-one-out MR interval estimate, for MDD (B) and ASRD (D), respectively. Interval estimates are expressed as odds ratios (ORs) of risk of MDD or ASRD per unit increase in log odds of prescription opioid use. Heterogeneity tests did not indicate heterogeneity in the IVW estimates of prescription opioid use on either risk of MDD or ASRD; pleiotropy robust methods indicated no bias in the IVW estimates (eTable 15 in the Supplement).

Single-variable conventional MR analysis showed only genetically determined prescription opioid use having an effect estimate consistent with increased risk for both MDD (IVW OR, 1.14; 95% CI, 1.06-1.22; *P* < .001) and ASRD (IVW OR, 1.24; 95% CI, 1.07-1.44; *P* = .004) (Table 1 and Figure 2; eTables 15-18 in the Supplement). These estimates were broadly consistent with estimates from the weighted median and MR-Egger sensitivity analyses; the MR-Egger estimates were substantially less precise (MDD WMOR, 1.18; 95% CI, 1.07-1.30; *P* < .001; ASRD WM OR, 1.22; 95% CI, 1.00-1.49; *P* = .05; MDD MR-Egger OR, 1.17; 95% CI, 0.92-1.47; *P* = .21; ASRD MR Egger OR, 1.26; 95%CI, 0.78-2.02; *P* = .36). The MR-Egger intercept analysis did not indicate horizontal pleiotropy. Conventional IVW leave-one-out analysis did not identify any high leverage points with high influence (Figure 2; eFigures 1-5 in the Supplement). The *F* statistics for the genetic instruments were consistent with an absence of weak instrument bias.

**Table 1.** Single-Variable MR Results of Prescription Opioid and Nonopioid Pain Medication Use on Risk of MDD and ASRD^a^^,^^b^

Exposure	Method	MDD			ASRD		
		N SNV	OR (95% CI)	*P* value	N SNV	OR (95% CI)	*P* value
Opioid use	IVW	26	1.14 (1.06-1.22)	<.001	23	1.24 (1.07-1.44)	.004
	Weighted median	26	1.18 (1.07-1.30)	<.001	23	1.22 (1.00-1.49)	.05
	MR Egger	26	1.17 (0.92-1.47)	.21	23	1.26 (0.78-2.02)	.36
Salicylic acid use	IVW	27	1.07 (0.98-1.18)	.15	21	1.18 (1.00-1.40)	.05
	Weighted median	27	1.04 (0.92-1.18)	.52	21	1.14 (0.91-1.44)	.25
	MR Egger	27	0.83 (0.66-1.04)	.12	21	0.92 (0.56-1.52)	.75
Anilide use	IVW	29	1.14 (1.01-1.27)	.03	34	1.01 (0.82-1.25)	.93
	Weighted median	29	1.14 (0.97-1.34)	.10	34	0.96 (0.70-1.30)	.77
	MR Egger	29	1.05 (0.67-1.64)	.82	34	0.85 (0.37-1.94)	.70
NSAID use	IVW	29	1.12 (1.02-1.23)	.02	29	1.29 (1.06-1.56)	.01
	Weighted median	29	1.20 (1.05-1.37)	.008	29	1.13 (0.86-1.47)	.38
	MR Egger	29	1.11 (0.78-1.58)	.56	29	1.18 (0.52-2.68)	.70

Abbreviations: ASRD, anxiety and stress-related disorder; GWAS, genome-wide association studies; IVW, inverse variance weighted; MDD, major depression or major depressive disorder (depending on study); MR, mendelian randomization; N SNVs, number of genetic instruments; NSAID, nonsteroidal anti-inflammatory drug; OR, odds ratio; SNV, single-nucleotide variant; SVMR, single-variable mendelian randomization.

^a^ Results from 2-sample SVMR analysis; main analysis method: outliers identified by MR PRESSO tool, removed; estimated associations reported as OR of outcome per unit increase in log odds of pain medication use.

^b^ Genetic instruments selected from opioid and nonopioid pain medication use GWASs, selection threshold *P* less than 5 × 10^−6^, pruned at linkage disequilibrium *R^2^* less than .001 (10 000 kilobase pair window); N SNV differs across outcomes depending on number of genetic instruments found in outcome GWASs.

In MVMR, assessing the genetic liabilities for prescription opioid and nonopioid pain medications use jointly, opioid use retained a robust relationship with both MDD (IVW OR, 1.14; 95% CI, 1.04-1.25; *P* = .005) and ASRD (IVW OR, 1.30; 95% CI, 1.08-1.56; *P* = .006) (Table 2; eTable 19 in the Supplement). These estimates were broadly consistent with estimates from the MVMR-Egger sensitivity analyses, although the MVMR-Egger estimates were again substantially less precise for MDD (OR, 1.13; 95% CI, 1.02-1.26; *P* = .02), and similarly, ASRD (OR, 1.18; 95% CI, 0.95-1.46; *P* = .13). The MR-Egger intercept analysis again did not indicate horizontal pleiotropy. In additional MVMR analyses, assessing the genetic liability for prescription opioid use jointly with site-specific pain, opioid use retained that association with both MDD and ASRD (eg, controlling for back pain: MDD IVW OR, 1.12; 95% CI, 1.05-1.21; *P* = .001; ASRD OR, 1.26; 95% CI, 1.09-1.45; *P* = .002), again, the MVMR-Egger estimates consistent but substantially less precise (MDD MR-Egger OR, 1.12; 95% CI, 0.95-1.30; *P* = .19; ASRD MR-Egger OR, 1.27; 95% CI, 0.90-1.80; *P* = .18). The MR-Egger intercept analysis again did not indicate horizontal pleiotropy (eTable 20 in the Supplement).

**Table 2.** Multivariable MR Results of Prescription Opioid and Nonopioid Pain Medications Use on Risk of MDD and ASRD^a^^,^^b^

MV exposures	Methods	MDD			ASRD		
		No. of SNVs	OR (95% CI)	*P* value	No. of SNVs	OR (95% CI)	*P* value
Opioid use	MV IVW	92	1.14 (1.04-1.25)	.005	86	1.30 (1.08-1.56)	.006
	MV Egger	92	1.13 (1.02-1.26)	.02	86	1.18 (0.95-1.46)	.13
NSAID use	MV IVW	92	0.98 (0.84-1.15)	.81	86	1.37 (0.99-1.90)	.06
	MV Egger	92	0.97 (0.82-1.15)	.72	86	1.23 (0.87-1.73)	.24
Salicylic acid use	MV IVW	92	0.99 (0.89-1.11)	.88	86	0.96 (0.76-1.21)	.70
	MV Egger	92	0.98 (0.87-1.11)	.76	86	0.84 (0.64-1.10)	.21
Anilide use	MV IVW	92	1.07 (0.89-1.29)	.46	86	0.71 (0.49-1.03)	.07
	MV Egger	92	1.06 (0.87-1.28)	.56	86	0.64 (0.43-0.94)	.02

Abbreviations: ASRD, anxiety and stress-related disorder; GWAS, genome-wide association studies; IVW, inverse variance weighted MR; MDD, major depression or major depressive disorder (depending on study used in GWAS); MR, mendelian randomization; MV IVW, multivariable inverse variance weighted; MVMR, multivariable mendelian randomization; N SNV, number of SNVs; OR, odds ratio; SNV, single-nucleotide variant.

^a^ Results from outlier-corrected 2-sample multivariable MR analysis, 2 multivariable complementary methods reported; main analysis method: estimates reported as OR of psychiatric outcome per unit increase in log odds of pain medication use exposure, accounting for other pain medication uses.

^b^ Instruments selected from opioid and nonopioid GWASs, selection threshold *P* less than 5 × 10^−6^, pruned at linkage disequilibrium *R^2^* less than 0.001 (10 000 kilobase pair window); outliers identified by MR PRESSO tool, removed; N SNVs differs across outcomes depending on number of instrument SNVs found in psychiatric outcome GWASs.

Bidirectional single-variable analyses showed the genetic liability for MDD, but not ASRD, having effect size estimates consistent with increased risk of use for all opioid and nonopioid pain medications (opioids OR, 1.18; 95% CI, 1.08-1.30; *P* < .001; anilides OR, 1.20; 95% CI, 1.00-1.28; *P* < .001; NSAIDs OR, 1.15; 95% CI, 1.09-1.22; *P* < .001; salicylic acid/derivatives OR, 1.10; 95% CI, 1.03-1.17; *P* = .002) (Table 3; eTables 15-18 in the Supplement). These estimates were broadly consistent with estimates from the weighted median and MR-Egger sensitivity analyses; the MR-Egger estimates were substantially less precise. The MR-Egger intercept analysis did not indicate horizontal pleiotropy. The *F* statistics for the genetic instruments were consistent with an absence of weak instrument bias.

**Table 3.** Single-Variable MR Results of Risk of MDD and ASRD on Risk of Prescription Opioid and Nonopioid Pain Medications Use ^a^^,^^b^

Exposure	Methods	Opioid use			Salicylic acid use			Anilide use			NSAIDs use		
		No. of SNVs	OR (95% CI)	*P* value	No. of SNVs	OR (95% CI)	*P* value	No. of SNVs	OR (95% CI)	*P* value	No. of SNVs	OR (95% CI)	*P* value
MDD	IVW	72	1.18 (1.08-1.30)	<.001	93	1.10 (1.03-1.17)	.002	89	1.20 (1.00-1.28)	<.001	92	1.15 (1.09-1.22)	<.001
	Weighted median	72	1.25 (1.10-1.42)	<.001	93	1.11 (1.01-1.21)	.02	89	1.22 (1.00-1.32)	<.001	92	1.17 (1.08-1.27)	<.001
	MR Egger	72	1.54 (1.01-2.34)	.05	93	1.22 (0.91-1.63)	.18	89	1.14 (1.43-1.50)	.36	92	1.08 (0.82-1.43)	.57
ASRD	IVW	17	1.04 (0.97-1.12)	.29	10	1.05 (0.99-1.11)	.11	17	1.02 (0.97-1.06)	.43	17	1.01 (0.96-1.06)	.80
	Weighted median	17	0.98 (0.90-1.07)	.70	10	1.06 (0.98-1.14)	.13	17	1.00 (0.95-1.06)	.92	17	1.02 (0.96-1.08)	.57
	MR Egger	17	1.01 (0.79-1.30)	.94	10	0.98 (0.82-1.16)	.79	17	0.99 (0.85-1.15)	.86	17	1.05 (0.89-1.24)	.59

Abbreviations: ASRD, anxiety and stress-related disorder; GWAS, genome-wide association studies; IVW, inverse-variance weighted; MDD, major depression or major depressive disorder (depending on study); MR, mendelian randomization; MV, multivariable; N SNV, number of SNVs; NSAID, nonsteroidal anti-inflammatory drug; OR, odds ratio; SNV, single-nucleotide variant; SVMR, single-variable mendelian randomization.

^a^ Results from 2-sample SVMR analysis; main analysis method: IVW is boldfaced; outliers identified by MR PRESSO tool, removed; estimated associations reported as OR of pain medication use per unit increase in log odds of psychiatric exposure.

^b^ Genetic instruments selected from MDD and ASRD GWASs, selection threshold *P *less than 5 × 10^−6^, pruned at linkage disequilibrium *R^2 ^*less than 0.001 (10 000 kilobase pair window); N SNV differs across outcomes depending on number of genetic instruments found in outcome GWASs.

## Discussion

We evaluated potential bidirectional associations between the genetic liability for prescription opioid and nonopioid pain medication use and both MDD and ASRD and found genetic evidence that prescription opioid use was associated with increased MDD and ASRD risk, while strikingly, nonopioid analgesics had no direct association with the risk for MDD or ASRD.

Opioid MDD MR estimates were consistent in magnitude and direction across IVW, weighted median, and MR-Egger analyses, with the MR-Egger estimate substantially less precise, as is typically expected in MR genetic association studies, and the MR Egger intercept terms, being close to zero, consistent with absence of pleiotropy.^47^ Our findings extend observational literature suggesting prescription opioid use increases the risk for MDD,^19,20,21^ and unlike 2020 evidence for a causal effect of *ICD*-defined OUD on MDD,^48^ our opioid use instrument only included prescription opioids and not heroin, suggesting important neuropsychiatric implications, beyond the risk for opioid misuse, and OUD, that may be considered when prescribing opioids.

The underlying mechanisms of prescription opioids in the pathophysiology of MDD remain to be elucidated, but potentially include opioid-induced dysregulation of reward circuitry that results in reduced reward perception or pleasure and relief generation^49^ or other physical medical dysregulation (ie, endocrine and autonomic nervous system abnormalities^50^) that potentially contributes to the physical symptoms of MDD. Notably, it has been suggested that the endogenous opioid system is directly involved in the regulation of mood and the dysregulation of that system may factor into depression and anxiety^11,51,52^; μ-opioid receptors (MOR) are widely distributed within the brain, including regions involved in emotion regulation,^11^ and κ-opioid receptors (KOR) are expressed in the cortex, striatum, hippocampus, amygdala, and thalamus,^53^ suggesting a role in reward, pain, and emotion. Opioid receptor antagonism has also been shown to attenuate ketamine’s antidepressant effects,^54^ and subeuphoric doses of partial opioid agonists have been shown to improve MDD symptoms.^11^ However, while small opioid doses may improve mood by activating MORs, prolonged opioid use may saturate MORs and activate the KOR, which also modulate mood^55,56^ and are associated with depression^54,57^; in rodents, KOR agonists increase anxiety while deficiencies in the KOR system decrease anxiety.^51^ In humans, prolonged opioid use (30 days or more), which may saturate the opioid receptor system to affect mood,^54^ increased risk for developing treatment-resistant depression by more than 25%, compared with opioid use for less than 30 days.^21^ Elucidating the underlying mechanisms of the opioid system dysregulation potentially shared between MDD and OUD may be important to combat these crises.^11^

Our bidirectional analyses, with genetic liability for prescription opioid use as an outcome, point to genetic liability for MDD but not ASRD as a possible causal risk factor of opioid use in psychiatric populations. Depression severity has been shown to be associated with increasing likelihood of misusing opioid medications for nonpain symptoms and self-increasing opioid dosage^58^ and up to 30% of long-term opioid users who have MDD qualify for moderate-to-severe OUD,^15,59^ suggesting that targeting opioid use prevention for patients with MDD may help mitigate the US opioid epidemic.^1,10^ Given more than half of individuals with OUD have comorbid MDD,^10^ the increased use of opioids by individuals with MDD may be owing to self-medication of social or emotional pain,^11^ suggesting that the development of therapeutic interventions with minimal risk targeted at endogenous opioid dysregulation represents another important prevention opportunity.^11^ In sum, our findings support recommendations that caution is needed with prescribing in settings of mood disorders in favor of nonopioid alternatives, with screening for MDD prior to initiating opioid treatment.^10^ Further, our findings that the genetic liability for MDD increased the risk of the genetic liability to take NSAIDs, anilide, and salicylic acid and derivatives support the well-known association between depression and physical pain; comorbid depression and pain experience reduce physical, mental, and social functioning when beyond either depression or pain.^60^ Further still, our findings that genetic liability for NSAIDs use increased the risk for MDD and ASRD supports observational evidence suggesting NSAIDs have adverse neural effects, including neuropsychiatric symptoms,^61,62^ although the association direction is opposite to reported RCTs using NSAIDs as adjunct therapies for depression.^63^ The NSAID-related adverse neuropsychiatric symptoms are most often reported with indomethacin and selective cyclooxygenase-2 inhibitors, which may modulate neural processes and synaptic signaling processes where cyclooxygenase-2 is localized.^61^

### Strengths and Limitations

This innovative 2-sample MR study investigating the association between genetic liability for opioid and other pain medication use and neuropsychiatric outcomes has several strengths. We use summary genetic associations from the largest available GWASs, important genetic analyses investigating small effect sizes; generally, larger sample sizes increase measurement precision. We also use complementary 2-sample MR methods for sensitivity analysis.^64^ The MVMR models are a major strength, enabling us to account for potential confounding owing to chronic pain, and also to estimate the direct association for genetic liability for prescription opioid use. Relatedly, including nonopioid pain medications provided additional context to the prescription opioid findings because none of the nonopioid pain medications retained a direct association with our neuropsychiatric outcomes in MVMR analyses. Finally, the list of prescribed prescription opioids comprising our opioid variable excludes heroin and other illicit opioid substances, enabling us to evaluate the specific association between the genetic liability for prescribed opioids and MDD.

There are also limitations to this study. The pain medication use and psychiatric disorder SNV effect estimates were obtained in mostly European studies, thus minimizing the possibility of population stratification bias and increasing the plausibility of the 2-sample MR assumption that summary associations derived from comparable populations.^39,65^ We performed sensitivity analyses to assess and minimize heterogeneity and pleiotropy. Nonetheless, we emphasize the importance of triangulating multiple lines of experimental evidence to strengthen causal inference.^66^

Regarding genetic instrument selection, following the example of prior MR studies,^67,68^ we used a stringent selection threshold (*P* < 5 × 10^−6^) for the pain medication use and ASRD risk instruments to compensate for lack of SNVs with effect *P* values less than conventional genome-wide significance (*P* < 5 × 10^−8^). We also used stringent LD clumping thresholds to ensure instrument independence. The biologic mechanisms of the selected SNVs are unknown; however, sensitivity analyses failed to find evidence for horizontal pleiotropy. Further, each pain medication phenotype was a binary variable (use/nonuse), so we could not assess potential dose-dependent changes in risk associated with prescription opioid use. Also, the genetic variants for pain medication use may be related to underlying disease, pain conditions, or even subclinical levels of the traits that influence MDD risk,^28^ which may affect results, although we note the association of the genetic liability of opioid use found in single-variable MR persisted in MVMR accounting for genetic liability for site-specific chronic pain. Nonetheless, future genetic studies using detailed hospital-based information about pain medication and including negative control populations would possibly allow to further strengthen causal inference. Finally, as has been noted previously, the UKB cohorts may not represent the general UK population,^69^ and analyses were limited to individuals of European ancestry; caution is warranted before generalizing findings to other populations.

## Conclusions

We provide preliminary genetic evidence that prescription opioid use increases MDD and ASRD risk, suggesting important clinical consequences. We also find genetic evidence that MDD is a potential causal risk factor for increased prescription opioid use, which may help identify patient populations to aim prevention strategies to curb the ongoing opioid epidemic.

## Notes

Supplement. **eMethods.** Detailed Methods. **eTable 1.** Neuropsychiatric Disorders and Prescription Medication Use Phenotypes: Source and Description **eTable 2.** Opioid and Non-Opioid Pain Medication and Active Ingredients by ATC Category in 318 177 UK Biobank European Individuals **eTable 3.** Independent Instruments for Prescription Opioid Use: Harmonized Data **eTable 4.** Independent Instruments for Salicylic Acid Use: Harmonized Data **eTable 5.** Independent Instruments for Anilides Use: Harmonized Data **eTable 6.** Independent Instruments for NSAID Use: Harmonized Data **eTable 7.** Independent Instruments for Anxiety and Stress Related Disorder: Harmonized Data **eTable 8.** Independent Instruments for Major Depressive Disorder: Harmonized Data **eTable 9.** Multivariable Instrument for Medications Use: Independent Instruments for Prescription Opioid and Non-Opioid Pain Medications **eTable 10.** Multivariable Instruments for Exposures Prescription Opioid Use and Abdominal/ Stomach Pain for 3+ Months in UKB Participants on MDD and ASRD: Data Harmonized **eTable 11.** Multivariable Instruments for Exposures Prescription Opioid Use and Neck/ Shoulder Pain for 3+ Months in UKB Participants on MDD and ASRD: Data Harmonized **eTable 12.** Multivariable Instruments for Exposures Prescription Opioid Use and Pain for 3+ Months in UKB Participants on MDD and ASRD: Data Harmonized **eTable 13.** Multivariable Instruments for Exposures Prescription Opioid Use and Knee Pain for 3+ Months in UKB Participants on MDD and ASRD: Data Harmonized **eTable 14.** Multivariable Instruments for Exposures Prescription Opioid Use and Headache Pain for 3+ Months in UKB Participants on MDD and ASRD: Data Harmonized **eTable 15.** Bidirectional Single Variable Mendelian Randomization Results of Opioid Use Risk in UK Biobank Participants Versus Neuropsychiatric Disorders **eTable 16.** Bidirectional Single Variable Mendelian Randomization Results of Salicylic Acid and Derivatives Use Risk in UK Biobank Participants Versus Neuropsychiatric Disorders **eTable 17.** Bidirectional Single Variable Mendelian Randomization Results of Anilide Use Risk in UK Biobank Participants Versus Neuropsychiatric Disorders **eTable 18.** Bidirectional Single Variable Mendelian Randomization Results of NSAID Use Risk in UK Biobank Participants Versus Neuropsychiatric Disorders **eTable 19.** Multivariable Mendelian Randomization Results of Opioid and Non-Opioid Use Risk in UK Biobank Participants on Neuropsychiatric Disorders **eTable 20.** Multivariable Mendelian Randomization Results of Opioid Use Risk Accounting for Chronic Pain in UK Biobank Participants on MDD and ASRD **eFigure 1.** Leave-1-Out Analysis of Associations of Genetic Risk of Salicylic Acid/ Derivatives Use on Risk of MDD and ASRD **eFigure 2.** Leave-1-Out Analysis of Associations of Genetic Risk of Anilide Use on Risk of MDD and ASRD **eFigure 3.** Leave-1-Out Analysis of Associations of Genetic Risk of NSAID Use on Risk of MDD and ASRD **eFigure 4.** Leave-1-Out Analysis of Associations of Genetic Risk of ASRD on Risk of Pain Medications Use **eFigure 5.** Leave-1-Out Analysis of Associations of Genetic Risk of MDD on Risk of Pain Medications Use
