# Supplementary material for: Prescription Opioid Use and Risk for Major Depressive Disorder and Anxiety and Stress-Related Disorders: A Multivariable Mendelian Randomization Analysis
Source: JAMA Psychiatry. 2020 Nov 11;78(2):1–10. doi: 10.1001/jamapsychiatry.2020.3554 (PMC7658804; doi:10.1001/jamapsychiatry.2020.3554)
Supplement: Supplement. — eMethods. Detailed Methods. eTable 1. Neuropsychiatric Disorders and Prescription Medication Use Phenotypes: Source and Description eTable 2. Opioid and Non-Opioid Pain Medication and Active Ingredients by ATC Category in 318 177 UK Biobank European Individuals eTable 3. Independent Instruments for Prescription Opioid Use: Harmonized Data eTable 4. Independent Instruments for Salicylic Acid Use: Harmonized Data eTable 5. Independent Instruments for Anilides Use: Harmonized Data eTable 6. Independent Instruments for NSAID Use: Harmonized Data eTable 7. Independent Instruments for Anxiety and Stress Related Disorder: Harmonized Data eTable 8. Independent Instruments for Major Depressive Disorder: Harmonized Data eTable 9. Multivariable Instrument for Medications Use: Independent Instruments for Prescription Opioid and Non-Opioid Pain Medications eTable 10. Multivariable Instruments for Exposures Prescription Opioid Use and Abdominal/ Stomach Pain for 3+ Months in UKB Participants on MDD and ASRD: Data Harmonized eTable 11. Multivariable Instruments for Exposures Prescription Opioid Use and Neck/ Shoulder Pain for 3+ Months in UKB Participants on MDD and ASRD: Data Harmonized eTable 12. Multivariable Instruments for Exposures Prescription Opioid Use and Pain for 3+ Months in UKB Participants on MDD and ASRD: Data Harmonized eTable 13. Multivariable Instruments for Exposures Prescription Opioid Use and Knee Pain for 3+ Months in UKB Participants on MDD and ASRD: Data Harmonized eTable 14. Multivariable Instruments for Exposures Prescription Opioid Use and Headache Pain for 3+ Months in UKB Participants on MDD and ASRD: Data Harmonized eTable 15. Bidirectional Single Variable Mendelian Randomization Results of Opioid Use Risk in UK Biobank Participants Versus Neuropsychiatric Disorders eTable 16. Bidirectional Single Variable Mendelian Randomization Results of Salicylic Acid and Derivatives Use Risk in UK Biobank Participants Versus Neuropsychiatric Disorders eTable 17. [file jamapsychiatry-e203554-s001.pdf]

Supplemental Online Content

Rosoff DB, Smith GD, Lohoff FW. Prescription opioid use and risk for major depressive disorder: a multivariable mendelian randomization analysis. *JAMA Psychiatry*. Published online November 11, 2020. doi:10.1001/jamapsychiatry.2020.3554

eMethods. Detailed Methods.
eTable 1. Neuropsychiatric Disorders and Prescription Medication Use Phenotypes: Source and Description
eTable 2. Opioid and Non-Opioid Pain Medication and Active Ingredients by ATC Category in 318 177 UK Biobank European Individuals
eTable 3. Independent Instruments for Prescription Opioid Use: Harmonized Data
eTable 4. Independent Instruments for Salicylic Acid Use: Harmonized Data
eTable 5. Independent Instruments for Anilides Use: Harmonized Data
eTable 6. Independent Instruments for NSAID Use: Harmonized Data
eTable 7. Independent Instruments for Anxiety and Stress Related Disorder: Harmonized Data
eTable 8. Independent Instruments for Major Depressive Disorder: Harmonized Data
eTable 9. Multivariable Instrument for Medications Use: Independent Instruments for Prescription Opioid and Non-Opioid Pain Medications
eTable 10. Multivariable Instruments for Exposures Prescription Opioid Use and Abdominal/ Stomach Pain for 3+ Months in UKB Participants on MDD and ASRD: Data Harmonized
eTable 11. Multivariable Instruments for Exposures Prescription Opioid Use and Neck/ Shoulder Pain for 3+ Months in UKB Participants on MDD and ASRD: Data Harmonized
eTable 12. Multivariable Instruments for Exposures Prescription Opioid Use and Pain for 3+ Months in UKB Participants on MDD and ASRD: Data Harmonized
eTable 13. Multivariable Instruments for Exposures Prescription Opioid Use and Knee Pain for 3+ Months in UKB Participants on MDD and ASRD: Data Harmonized
eTable 14. Multivariable Instruments for Exposures Prescription Opioid Use and Headache Pain for 3+ Months in UKB Participants on MDD and ASRD: Data Harmonized
eTable 15. Bidirectional Single Variable Mendelian Randomization Results of Opioid Use Risk in UK Biobank Participants Versus Neuropsychiatric Disorders
eTable 16. Bidirectional Single Variable Mendelian Randomization Results of Salicylic Acid and Derivatives Use Risk in UK Biobank Participants Versus Neuropsychiatric Disorders
eTable 17. Bidirectional Single Variable Mendelian Randomization Results of Anilide Use Risk in UK Biobank Participants Versus Neuropsychiatric Disorders
eTable 18. Bidirectional Single Variable Mendelian Randomization Results of NSAID Use Risk in UK Biobank Participants Versus Neuropsychiatric Disorders

© 2020 Rosoff DB et al. *JAMA Psychiatry.*

eTable 19. Multivariable Mendelian Randomization Results of Opioid and Non-Opioid Use Risk in UK Biobank Participants on Neuropsychiatric Disorders
eTable 20. Multivariable Mendelian Randomization Results of Opioid Use Risk Accounting for Chronic Pain in UK Biobank Participants on MDD and ASRD
eFigure 1. Leave-1-Out Analysis of Associations of Genetic Risk of Salicylic Acid/ Derivatives Use on Risk of MDD and ASRD
eFigure 2. Leave-1-Out Analysis of Associations of Genetic Risk of Anilide Use on Risk of MDD and ASRD
eFigure 3. Leave-1-Out Analysis of Associations of Genetic Risk of NSAID Use on Risk of MDD and ASRD
eFigure 4. Leave-1-Out Analysis of Associations of Genetic Risk of ASRD on Risk of Pain Medications Use
eFigure 5. Leave-1-Out Analysis of Associations of Genetic Risk of MDD on Risk of Pain Medications Use

This supplemental material has been provided by the authors to give readers additional information about their work.

© 2020 Rosoff DB et al. *JAMA Psychiatry*.

eMethods. Detailed Methodology.

Opioid and Non-Opioid Pain Medication Use

We used summary genetic association statistics from a recent medication use case-control GWAS published in 2019 based on 318 177 individuals of European ancestry from the UK Biobank (UKB) prospective cohort study (collected across the United Kingdom from 2006-2010).[1] In the overall starting cohort of 502 616 UK Biobank participants with medication records, approximately 54% were female, with mean age 56.5 (standard deviation 8.1years); the proportion of participants taking medications increased with age and was higher for females than males: approximately 67% females took medications at 40 years of age, and 90% at 70, compared to 50% males at age 40 years, and 85% at 70. UKB classified medications into 6 745 categories; only 1 809 were reported by ≥ 10 participants: 1752 were classified (classifiable) by active ingredient using the Anatomical Therapeutic Chemical Classification System (ATC) and then assigned to 23 categories by active ingredient, including but not limited to opioids (e.g. morphine, oxycodone, codeine, fentanyl, pethidine, and tramadol), non-steroidal anti-inflammatory and antirheumatic products, anilides and salicylic acid products (the medications included in the present study are listed in eTable 2 in the Supplement). For the GWAS, 318 177 of the initial cohort remained having non-blank medication records and taking any of the 1 752 medications taken by ≥ 10 participants and classifiable using the ATC.

Major Depression and/or Major Depressive Disorder (MDD)

We used summary statistics from the recent PGC GWAS meta-analysis published in 2018 based on 135 458 cases and 344 901 controls from 35 studies from populations of predominantly European ancestry.[2] The included studies used a range of methods for assessing MDD or major depression: cases were required to meet international consensus criteria (DSM-IV, ICD-9, or ICD-10) for a lifetime diagnosis of MDD, established based upon any of the following: structured diagnostic instruments from assessments by trained interviewers; clinician-administered checklists or medical record reviews; electronic hospital records, or national inpatient records; and in two cohorts, self-report (i.e. in the UKB cohort, self-identification as MDD inpatient, MDD or anxiety psychiatric out-patient, or depression or anhedonia diagnosis; in the 23andMe cohort, self-identification of diagnosed clinical depression or depression). Most of the studies included in the PGC GWAS meta-analysis applied typical inclusion and exclusion criteria for both cases and controls (e.g. excluding cases with lifetime bipolar disorder or schizophrenia and excluding controls with major depression). Notwithstanding the range of methods for assessing major depressive disorder or major depression (combined, MDD), the cohorts were determined to be genetically comparable (for purposes of the GWAS). Due to the restrictions on the use of the 23andMe cohort, and to minimize sample overlap (see section, below), we used summary statistics from the meta-analysis done excluding both the UKB (14 260 cases and 15 480 controls) and 23andMe cohorts (59 851 cases and 113 154 controls) to extract SNP outcome effects corresponding to pain medications use instrument SNPs for the single variable Mendelian randomization (SVMR) analyses of pain medication use risk. We used the meta-analysis done with both UKB and 23andMe cohorts (total N = 480 359).

Anxiety and Stress-Related Disorder (ASRD)

We used summary statistics from the recent iPSYCH GWAS based on 12 655 individuals with anxiety and stress-related diagnoses and 19 225 controls from Denmark: all individuals participated in iPSYCH, a study designed to disentangle risk factors of severe psychiatric disorders, including schizophrenia, autism, attention-deficit/hyperactivity disorder, anorexia nervosa, and affective disorders.[3] The cases were identified through the national research registers on the basis of having a record of an anxiety or stress-related diagnosis assigned by a psychiatrist during routine clinical care according to the international Statistical Classification of Diseases and Related Health Problems (10th Revision), excluding individuals with comorbid autism. Controls comprised individuals not diagnosed with anxiety, stress-related disorders, or mood disorders. The GWAS was performed [2]using logistic regression, with covariates including the first 4 principal components to control for remaining population stratification; the results were meta-analyzed using an inverse variance–weighted fixed-effects model.

Pain-Related Covariates

To account for pain in our MVMR analyses, we extracted SNPs from the MRC-IEU UK Biobank GWAS based on the responses of individuals of European ancestry from the UK Biobank prospective cohort study.[4] We included all independent SNPs associated with opioid use and each of six PHESANT-derived phenotypes from the UK Biobank

© 2020 Rosoff DB et al. *JAMA Psychiatry.*

binary variables ("Yes/No") related to site-specific chronic pain occurring for 3+ months: back (UK Biobank data-field 3571); knee (UK Biobank data-field 3773); hip (UK Biobank data-field 3414); neck/shoulder (UK Biobank data-field 3404); headache (UK Biobank data-field 3799); abdominal/stomach (UK Biobank data-field 3741). We pruned the results to exclude all SNPs with a pairwise LD (r^2 > .001, 10 000 kb window), then extracted the resulting multivariable MR instrument SNPs from the MDD and ASRD GWASs, and harmonized exposure and outcome data (eTables 10-14 in the Supplement).

Sample Independence

Participant overlap in samples used to estimate genetic associations between exposure and outcome in two sample MR can bias results,[5,6] so we endeavored to minimize overlapping to reduce this source of weak instrument bias. In the present study, we avoided overlap between the cohorts used in the UK Biobank medication use GWASs vis-à-vis those used in the iPSYCH ASRD and the PGC MDD GWASs, the latter using the PGC MDD GWAS excluding both UK Biobank (*N* = 29 740) and 23andMe cohorts (*N* = 307 354). However, to improve precision for the bidirectional analyses, we used the PGC MDD GWAS including both UK Biobank and 23and Me cohorts for the largest sample size publicly available (*N* = 480 359) and extracted exposure instruments therefrom: the single common cohort (between MDD vis-à-vis opioid and non-opioid pain medication use) accounted for 6.2% of participants in the PGC MDD GWAS: the relevant percentage overlap for purposes of determining weak instrument bias (WIB) is taken with respect to the larger data set since only the presence of participants in both studies leads to correlation in estimates.[6] Given the strength of the instruments for MDD (the combined instrument *F* statistic ranged from 31.7 to 32.3; the corresponding lower limit of the 95% confidence interval of the estimate ranged from 29.9 to 30.4 (eTables 15-18 in the Supplement)), and simulation studies of the association between sample overlap and the degree of WIB, considerable WIB bias is not expected.[6] We were unable to calculate conditional *F*-statistics to assess the strength of our multivariable instruments: SVMR statistical methods recently extended by Sanderson et al. to two sample MVMR are appropriate only for non-overlapping exposure summary level data sources; when overlapping, the requisite pairwise covariances between SNP associations are determinable only using individual level data.[7]

Statistical Analysis

For SVMR, applied to assess the total effects of each of the opioid and non-opioid pain medication use risk on each of MDD and ASRD, and vice versa, we used inverse-variance weighted MR (MR IVW) (single variable weighted linear regression) as the main method. We included MR Egger, weighted median, and weighted mode methods to assess the evidence of the causal effects of opioid and non-opioid pain medication use on risk of each of the neuropsychiatric outcomes, and vice versa, so as to detect the sensitivity of our results to different patterns of violations of IV assumptions: consistency of results across MR methods strengthens an inference of causality.[8] MR IVW is regarded as the main method: in the absence of pleiotropy and assuming the instruments are valid, MR IVW returns unbiased estimates of a causal effect so long as horizontal pleiotropy is balanced.[8,9] MR Egger extends MR IVW by not setting the intercept to zero, thus allowing the net horizontal pleiotropic effect across all SNPs to be unbalanced or directional (i.e. some SNPs could be acting on the outcome through a pathway other than through the exposure).[9,10] MR Egger returns unbiased causal effect estimates even if the assumption of no horizontal pleiotropy is violated for all SNPs, but the estimates may be substantially less precise than MR IVW: while the precision of the IVW estimate depends on the proportion of variance in the exposure explained by the genetic instruments, MR Egger precision depends as well on the variability of the SNP exposure effects. Weighted median MR uses the median effect of all available SNPs, so that only half of the SNPs need to be valid instruments (i.e. no horizontal pleiotropy, no association with confounders, and robust association with the exposure) to return an unbiased causal effect estimate. Stronger SNPs contribute more towards the causal estimate, with the contribution of each SNP weighted by the inverse variance of its association with the outcome.[11] Weighted mode-based MR clusters the SNPs into groups based on similarity of causal effects and returns the causal effect estimate based on the cluster that has the largest number of SNPs: unbiased causal effects so long as the SNPs within the largest cluster are valid instruments. Weighted mode MR weights each SNP's contribution to the clustering by the inverse variance of its outcome effect. Assuming the most common causal effect is consistent, the estimated causal effects would be unbiased even if all other instruments are invalid.[12]

Multivariable Mendelian Randomization

We estimated the direct effect of each exposure using multivariable Mendelian randomization (MVMR). MVMR simultaneously estimates the effects of multiple exposures using multiple sets of overlapping SNPs identified in the

relevant GWASs at $P < 5x10^{-6}$ (less than conventional genome wide significance) as instruments, pruned to exclude duplicative SNPs, as well as SNPs in LD ($R^2 > .001$, 10 000 kilobase pair windows, using the 1000 Genomes Reference Panel) with other SNPs.

For MVMR, applied to assess the direct or independent effects of opioid and non-opioid pain medication use on MDD and ASRD, we used the extension developed by Burgess et al.[5] of the inverse-variance weighted MR method, performing multivariable weighted linear regression (variants uncorrelated, random effect model) with the intercept term set to zero,[9] and, additionally, the MVMR extension of the MR Egger method to correct for both measured and unmeasured pleiotropy.[13]

Sensitivity Analyses and Diagnostics

To evaluate heterogeneity in genetic instruments effects, indicating potential violations of the instrumental variable (IV) assumptions, we used the MR Egger intercept test,[14] the Cochran Q heterogeneity test,[15] and the MR pleiotropy residual sum and outlier (MR-PRESSO) test.[16] A MR Egger regression intercept is generally interpreted as the average pleiotropic effect across all instruments: MR-Egger regression thus provides a test for average pleiotropy, and further has been extended to correct for both measured and unmeasured pleiotropy in MVMR.[13] The Cochran Q test, used to identify outliers in regression analysis generally, has been applied in MR to detect average pleiotropy: pleiotropy can induce heterogeneity of individual ratio estimates.[16] MR-PRESSO detects pleiotropic bias in MR caused by violation of the exclusion restriction IV assumption; extending the principal of the Cochran *Q* test, MR-PRESSO provides a global test to detect pleiotropic bias and identify sources of the bias (so called outlier SNPs), and further has been extended to detect pleiotropic bias also in MVMR [16]. We used the MR-PRESSO global test to identify outlier SNPS; removing the outlier SNPs, we reran the MR, and retested so as to determine whether removing outliers resolved the detected heterogeneity.

Two sample MR is normally conceptualized as meta-analyzing the Wald ratios from each instrument, but this is of course equivalent to a weighted regression of the SNP exposure effects on the SNP outcome effects. We have implicitly tested linear regression violations – e.g. median based estimator tests how sensitive the results are to outliers.[17] We used Steiger filtering to identify and exclude instruments that exhibited reverse causation.[18] We also used MR PRESSO methods to identify and remove points of high leverage that may be driving the results.[16] Finally, we performed leave-one-out analysis (leaving each genetic variant out of the IVW MR analysis in turn) to assess whether points of high leverage were points of high influence[18] (analyses plotted Figure 2; eFigures 1-5 in the Supplement).

We estimated the total effects of risk of opioid and non-opioid pain medication use on MDD and ASRD, and vice versa in a bidirectional check, using two-sample single variable Mendelian randomization (Tables 1 and 2; Figure 2; eTables 15-18 in the Supplement), which approach estimates the effect including any effects mediated or moderated through any other trait.[19] Results for the former are reported as odds ratios (OR) of MDD or ASRD risk per unit increase in log odds of the opioid or non-opioid use, and results for the latter are reported as OR of opioid or non-opioid use per unit increase in the log odds of MDD or ASRD.

References

1. Wu Y, Byrne EM, Zheng Z, et al. Genome-wide association study of medication-use and associated disease in the UK Biobank. *Nature Communications.* 2019;10(1):1891.
2. Wray NR, Ripke S, Mattheisen M, et al. Genome-wide association analyses identify 44 risk variants and refine the genetic architecture of major depression. *Nat Genet.* 2018;50(5):668-681.
3. Meier SM, Trontti K, Purves KL, et al. Genetic Variants Associated With Anxiety and Stress-Related Disorders: A Genome-Wide Association Study and Mouse-Model Study. *JAMA Psychiatry.* 2019;76(9):924-932.
4. Elsworth B, Mitchell, R, Raistrick, CA, Paternoster, L, Hemani, G, Gaunt, TR MRC IEU UK Biobank GWAS pipeline version 1. 2017. doi: https://doi.org/10.5523/bris.2fahpksont1zi26xosyamqo8rr. Accessed Feb 2, 2019.
5. Burgess S, Thompson SG. Multivariable Mendelian randomization: the use of pleiotropic genetic variants to estimate causal effects. *Am J Epidemiol.* 2015;181(4):251-260.

© 2020 Rosoff DB et al. *JAMA Psychiatry.*

6. Burgess S, Davies NM, Thompson SG. Bias due to participant overlap in two-sample Mendelian randomization. *Genet Epidemiol.* 2016;40(7):597-608.
7. Sanderson E, Spiller W, Bowden J. Testing and Correcting for Weak and Pleiotropic Instruments in Two-Sample Multivariable Mendelian Randomisation. *bioRxiv (preprint).* 2020:2020.2004.2002.021980.
8. Hemani G, Zheng J, Elsworth B, et al. The MR-Base platform supports systematic causal inference across the human phenome. *Elife.* 2018;7.
9. Bowden J, Smith GD, Burgess S. Mendelian randomization with invalid instruments: effect estimation and bias detection through Egger regression. *International Journal of Epidemiology.* 2015;44(2):512-525.
10. Davey Smith G, Bowden J, Del Greco M F, Minelli C, Thompson JR, Sheehan NA. Assessing the suitability of summary data for two-sample Mendelian randomization analyses using MR-Egger regression: the role of the I2 statistic. *International Journal of Epidemiology.* 2016;45(6):1961-1974.
11. Bowden J, Davey Smith G, Haycock PC, Burgess S. Consistent Estimation in Mendelian Randomization with Some Invalid Instruments Using a Weighted Median Estimator. *Genet Epidemiol.* 2016;40(4):304-314.
12. Hartwig FP, Davey Smith G, Bowden J. Robust inference in summary data Mendelian randomization via the zero modal pleiotropy assumption. *Int J Epidemiol.* 2017;46(6):1985-1998.
13. Rees JMB, Wood AM, Burgess S. Extending the MR-Egger method for multivariable Mendelian randomization to correct for both measured and unmeasured pleiotropy. *Stat Med.* 2017;36(29):4705-4718.
14. Bowden J, Del Greco MF, Minelli C, Smith GD, Sheehan N, Thompson J. A framework for the investigation of pleiotropy in two-sample summary data Mendelian randomization. *Statistics in Medicine.* 2017;36(11):1783-1802.
15. Bowden J, Del Greco MF, Minelli C, et al. Improving the accuracy of two-sample summary-data Mendelian randomization: moving beyond the NOME assumption. *Int J Epidemiol.* 2019;48(3):728-742.
16. Verbanck M, Chen CY, Neale B, Do R. Detection of widespread horizontal pleiotropy in causal relationships inferred from Mendelian randomization between complex traits and diseases. *Nat Genet.* 2018;50(5):693-698.
17. Bowden J, Davey Smith G, Haycock PC, Burgess S. Consistent Estimation in Mendelian Randomization with Some Invalid Instruments Using a Weighted Median Estimator. *Genet Epidemiol.* 2016;40(4):304-314.
18. Hemani G, Bowden J, Davey Smith G. Evaluating the potential role of pleiotropy in Mendelian randomization studies. *Human Molecular Genetics.* 2018;27(R2):R195-R208.
19. Sanderson E, Davey Smith G, Windmeijer F, Bowden J. An examination of multivariable Mendelian randomization in the single-sample and two-sample summary data settings. *Int J Epidemiol.* 2019;48(3):713-727.

© 2020 Rosoff DB et al. *JAMA Psychiatry.*

eTable 1. Neuropsychiatric Disorders and Prescription Medication Use Phenotypes: Source and Description

PHENOTYPES	Consortium	First Author (Year)	Sample Size	Cases	Controls	h	SE (h)	Population	Download
**Neuropsychiatric disorders:**									
STRESS RELATED DISORDER	iPSYCH	Maier (2019)[3]	31890	12665	19225	0.28	0.027	European	https://ipsych.dk/forskning/downloads/
MAJOR DEPRESSIVE DISORDER (outcome)	PGC	Wray (2018)[2]	143265	45591	97674	N/A	N/A	European	https://www.med.unc.edu/pgc/results-and-downloads/mdd/
MAJOR DEPRESSIVE DISORDER (exposure)	PGC	Wray (2018)	480359	135458	344901	0.09	0.004	European	https://www.med.unc.edu/pgc/results-and-downloads/mdd/
**Medications Use:**									
OPIOID USE	NA	Wu (2019)[1]	78808	22982	55826	0.15	0.008	European (UKB)	ftp://ftp.ebi.ac.uk/pub/databases/gwas/summary_statistics/WuY_31015401_GCST007936
ANILIDE USE	NA	Wu (2019)	179870	83218	96592	0.06	0.004	European (UKB)	ftp://ftp.ebi.ac.uk/pub/databases/gwas/summary_statistics/WuY_31015401_GCST007938
NSAID USE	NA	Wu (2019)	164520	74150	90370	0.06	0.004	European (UKB)	ftp://ftp.ebi.ac.uk/pub/databases/gwas/summary_statistics/WuY_31015401_GCST007934
SALICYLIC ACID AND DERIVATIVES USE	NA	Wu (2019)	112010	61583	50427	0.03	0.004	European (UKB)	ftp://ftp.ebi.ac.uk/pub/databases/gwas/summary_statistics/WuY_31015401_GCST007942
**Chronic Pain (3+ months):**									
Back	MRC-IEU	Elsworth (2018)[4]	117404	80588	36816	NA	NA	European (UKB)	https://gwas.mrcieu.ac.uk/datasets/ukb-b-8463
Neck/ Shoulder	MRC-IEU	Elsworth (2018)	105396	72887	32509	NA	NA	European (UKB)	https://gwas.mrcieu.ac.uk/datasets/ukb-b-16118
Hip	MRC-IEU	Elsworth (2018)	51516	40152	11369	NA	NA	European (UKB)	https://gwas.mrcieu.ac.uk/datasets/ukb-b-133
Headaches	MRC-IEU	Elsworth (2018)	91269	41719	49550	NA	NA	European (UKB)	https://gwas.mrcieu.ac.uk/datasets/ukb-b-13092
Knee	MRC-IEU	Elsworth (2018)	97889	76910	20979	NA	NA	European (UKB)	https://gwas.mrcieu.ac.uk/datasets/ukb-b-8906
Stomach/ Abdominal	MRC-IEU	Elsworth (2018	38911	21711	17200	NA	NA	European (UKB)	https://gwas.mrcieu.ac.uk/datasets/ukb-b-19097

**Abbreviations:**
PGC: Psychiatric Genomics Consortium
iPSYCH: The Lundbeck Foundation for Integrative Psychiatric Research
MRC-IEU: Medical Research Center-Integrative Epidemiology Center (UK Bristol)
UKB: UK Biobank
NSAID: Non-steroidal anti-inflammatory or antirheumatic drugs
MDD: Major depressive disorder/ major depression
h: heritability (reported in GWAS)
SE(h): standard error of h

© 2020 Rosoff DB et al. *JAMA Psychiatry*.

eTable 2. Opioid and Non-Opioid Pain Medication and Active Ingredients by ATC Category in 318 177 UK Biobank European Individuals

Category	Medication	UK Biobank Code	ATC Code	Drug Name/ Active Ingredient(s)
OPIOIDS	morphine	1140871692	N02AA01	Morphine
OPIOIDS	oramorph 10mg/5ml oral solution	1140882272	N02AA01	Morphine
OPIOIDS	mst continus 30mg m/r tablet	1140871780	N02AA01	Morphine
OPIOIDS	mst continus 10mg m/r tablet	1140871776	N02AA01	Morphine
OPIOIDS	zomorph 10mg m/r capsule	1141152986	N02AA01	Morphine
OPIOIDS	oramorph 100mg/5ml concentrated oral solution	1140882274	N02AA01	Morphine
OPIOIDS	sevredol 10mg tablet	1140871700	N02AA01	Morphine
OPIOIDS	zomorph 30mg m/r capsule	1141152988	N02AA01	Morphine
OPIOIDS	oramorph 10mg/5ml oral unit dose vial	1140871778	N02AA01	Morphine
OPIOIDS	mst continus 100mg m/r tablet	1140871786	N02AA01	Morphine
OPIOIDS	oramorph sr 10mg m/r tablet	1140871712	N02AA01	Morphine
OPIOIDS	mst continus 60mg m/r tablet	1140871782	N02AA01	Morphine
OPIOIDS	sevredol 20mg tablet	1140871704	N02AA01	Morphine
OPIOIDS	zomorph 100mg m/r capsule	1141152992	N02AA01	Morphine
OPIOIDS	oxycodone hydrochloride	1141171038	N02AA05	Oxycodone
OPIOIDS	oxycontin 10mg m/r tablet	1141171048	N02AA05	Oxycodone
OPIOIDS	oxycontin 20mg m/r tablet	1141171050	N02AA05	Oxycodone
OPIOIDS	oxynorm 5mg capsule	1141171066	N02AA05	Oxycodone
OPIOIDS	oxynorm 10mg capsule	1141170964	N02AA05	Oxycodone
OPIOIDS	oxycontin 5mg m/r tablet	1141180792	N02AA05	Oxycodone
OPIOIDS	oxycontin 40mg m/r tablet	1141171052	N02AA05	Oxycodone
OPIOIDS	oxynorm 20mg capsule	1141170966	N02AA05	Oxycodone
OPIOIDS	oxycontin 80mg m/r tablet	1141171054	N02AA05	Oxycodone
OPIOIDS	dihydrocodeine	1140884464	N02AA08	Dihydrocodeine
OPIOIDS	dhc continus 60mg m/r tablet	1140871920	N02AA08	Dihydrocodeine
OPIOIDS	df118 30mg tablet	1140856454	N02AA08	Dihydrocodeine
OPIOIDS	dhc continus 120mg m/r tablet	1140871926	N02AA08	Dihydrocodeine
OPIOIDS	codeine phosphate+kaolin 10mg/3g/10ml mixture	1140865654	N02AA59	Codeine |Kaolin
OPIOIDS	migraleve duopack tablet	1140872026	N02AA79	Acetaminophen |Codeine |Buclizine
OPIOIDS	feminax tablet	1141188522	N02AA79	Caffeine |Acetaminophen |Codeine |Scopolamine
OPIOIDS	pethidine	1140884388	N02AB02	Pethidine
OPIOIDS	durogesic 25micrograms/hour patch	1140911830	N02AB03	Fentanyl
OPIOIDS	durogesic 50micrograms/hour patch	1140911832	N02AB03	Fentanyl
OPIOIDS	durogesic 75micrograms/hour patch	1140911834	N02AB03	Fentanyl
OPIOIDS	durogesic 100micrograms/hour patch	1140911836	N02AB03	Fentanyl
OPIOIDS	co-proxamol	1140923348	N02AC54	Acetaminophen |Dextropropoxyphene
OPIOIDS	temgesic 200mcg sublingual tablet	1140871734	N02AE01	Buprenorphine

© 2020 Rosoff DB et al. *JAMA Psychiatry*.

Category	Medication	UK Biobank Code	ATC Code	Drug Name/ Active Ingredient(s)
OPIOIDS	temgesic 400mcg sublingual tablet	1140871738	N02AE01	Buprenorphine
OPIOIDS	transtec 35micrograms/hour transdermal patch	1141180012	N02AE01	Buprenorphine
OPIOIDS	transtec 70micrograms/hour transdermal patch	1141180020	N02AE01	Buprenorphine
OPIOIDS	buprenorphine	1140871732	N02AE01 |N07BC01	Buprenorphine
OPIOIDS	distalgesic tablet	1140868260	N02AJ	Acetaminophen |Dextropropoxyphene
OPIOIDS	co-dydramol	1140923350	N02AJ01	Acetaminophen |Dihydrocodeine
OPIOIDS	remedeine tablet	1140871684	N02AJ01	Acetaminophen |Dihydrocodeine
OPIOIDS	paracetamol+dihydrocodeine tartrate	1140882396	N02AJ01	Acetaminophen |Dihydrocodeine
OPIOIDS	paramol tablet	1140863552	N02AJ01	Acetaminophen |Dihydrocodeine
OPIOIDS	remedeine forte tablet	1140871686	N02AJ01	Acetaminophen |Dihydrocodeine
OPIOIDS	co-codamol	1140923346	N02AJ06	Acetaminophen |Codeine
OPIOIDS	paracetamol + codeine	1140882394	N02AJ06	Acetaminophen |Codeine
OPIOIDS	solpadol caplet	1140871688	N02AJ06	Acetaminophen |Codeine
OPIOIDS	syndol tablet	1140856342	N02AJ06	Acetaminophen |Codeine |Ketorolac
OPIOIDS	solpadol capsule	1141168122	N02AJ06	Acetaminophen |Codeine
OPIOIDS	tylex capsule	1140871680	N02AJ06	Acetaminophen |Codeine
OPIOIDS	zapain caplet	1141178052	N02AJ06	Acetaminophen |Codeine
OPIOIDS	solpadeine capsule	1141168650	N02AJ06	Caffeine |Acetaminophen |Codeine
OPIOIDS	kapake tablet	1140864070	N02AJ06	Acetaminophen |Codeine
OPIOIDS	codipar caplet	1141187304	N02AJ06	Acetaminophen |Codeine
OPIOIDS	solpadeine tablet	1141168648	N02AJ06	Acetaminophen |Codeine
OPIOIDS	kapake capsule	1141165512	N02AJ06	Acetaminophen |Codeine
OPIOIDS	solpadol effervescent tablet	1140871682	N02AJ06	Acetaminophen |Codeine
OPIOIDS	zapain capsule	1141178054	N02AJ06	Acetaminophen |Codeine
OPIOIDS	paracodol capsule	1140925778	N02AJ06	Acetaminophen |Codeine
OPIOIDS	migraleve yellow tablet	1140872030	N02AJ06	Acetaminophen |Codeine
OPIOIDS	kapake 30/500 effervescent tablet	1141190656	N02AJ06	Acetaminophen |Codeine
OPIOIDS	solpadeine forte dispersible tablet	1140856340	N02AJ06	Caffeine |Acetaminophen |Codeine
OPIOIDS	codis dispersible tablet	1140856336	N02AJ07	Codeine |Acetylsalicylic Acid
OPIOIDS	aspirin+codeine	1140882392	N02AJ07	Codeine |Acetylsalicylic Acid
OPIOIDS	co-codaprin	1140923344	N02AJ07	Codeine |Acetylsalicylic Acid
OPIOIDS	ibuprofen+codeine phosphate	1140878030	N02AJ08	Codeine |Ibuprofen
OPIOIDS	cuprofen plus tablet	1141190952	N02AJ08	Codeine |Ibuprofen
OPIOIDS	migraleve tablet	1141168554	N02AJ09	Acetaminophen |Codeine |Buclizine
OPIOIDS	solpadeine soluble effervescent tablet	1140856442	N02AJ09	Caffeine |Acetaminophen |Codeine
OPIOIDS	solpadeine plus soluble effervescent tablet	1141189064	N02AJ09	Caffeine |Acetaminophen |Codeine
OPIOIDS	solpadeine plus capsule	1141189008	N02AJ09	Caffeine |Acetaminophen |Codeine
OPIOIDS	solpadeine max tablet	1141167748	N02AJ09	Acetaminophen |Codeine
OPIOIDS	solpadeine plus tablet	1141189010	N02AJ09	Caffeine |Acetaminophen |Codeine
OPIOIDS	veganin tablet	1140856348	N02AJ09	Caffeine |Acetaminophen |Codeine

© 2020 Rosoff DB et al. *JAMA Psychiatry*.

Category	Medication	UK Biobank Code	ATC Code	Drug Name/ Active Ingredient(s)
OPIOIDS	propain tablet	1140856436	N02AJ09	Caffeine |Acetaminophen |Codeine |Diphenhydramine
OPIOIDS	propain caplet	1141172966	N02AJ09	Caffeine |Acetaminophen |Codeine |Diphenhydramine
OPIOIDS	tramacet 325mg/37.5mg tablet	1141190960	N02AJ13	Tramadol |Acetaminophen
OPIOIDS	tramadol	1140864992	N02AX02	Tramadol
OPIOIDS	zydol 50mg capsule	1140865000	N02AX02	Tramadol
OPIOIDS	zamadol 50mg capsule	1140928742	N02AX02	Tramadol
OPIOIDS	zamadol sr 100mg m/r capsule	1141153424	N02AX02	Tramadol
OPIOIDS	zydol sr 100mg m/r tablet	1140922636	N02AX02	Tramadol
OPIOIDS	zydol soluble 50mg tablet	1140928372	N02AX02	Morphine
OPIOIDS	meptazinol	1140881026	N02AX05	Meptazinol Hydrochloride
OPIOIDS	meptid 200mg tablet	1140881028	N02AX05	Meptazinol Hydrochloride
OPIOIDS	paracetamol+tramadol	1141190956	N02AX52	Tramadol |Acetaminophen
NSAID	indocid 25mg capsule	1140871354	M01AB01	Indomethacin
NSAID	sulindac	1140871604	M01AB02	Sulindac
NSAID	dicloflex 25mg e/c tablet	1140921828	M01AB05	Diclofenac
NSAID	voltarol 25mg e/c tablet	1140871168	M01AB05	Diclofenac
NSAID	diclomax sr 75mg m/r capsule	1140917394	M01AB05	Diclofenac
NSAID	rhumalgan 25mg e/c tablet	1140871180	M01AB05	Diclofenac
NSAID	volsaid retard 75mg m/r tablet	1140923920	M01AB05	Diclofenac
NSAID	motifene 75mg e/c+m/r capsule	1140909354	M01AB05	Diclofenac
NSAID	voltaren retard 100mg m/r tablet	1140877872	M01AB05	Diclofenac
NSAID	fenactol 25mg e/c tablet	1141182674	M01AB05	Diclofenac
NSAID	voltarol 100mg suppository	1140871174	M01AB05	Diclofenac
NSAID	rheumatac retard 75mg m/r tablet	1141167426	M01AB05	Diclofenac
NSAID	voltarene lp 100mg m/r tablet	1140877874	M01AB05	Diclofenac
NSAID	etodolac	1140871188	M01AB08	Etodolac
NSAID	lodine 200mg tablet	1140871196	M01AB08	Etodolac
NSAID	eccoxolac 300mg capsule	1141193170	M01AB08	Etodolac
NSAID	acemetacin	1140875278	M01AB11	Acemetacin
NSAID	ketorolac	1140884558	M01AB15 |S01BC05	Ketorolac
NSAID	aceclofenac	1140925806	M01AB16 |M02AA25	Aceclofenac
NSAID	arthrotec tablet	1140871266	M01AB55	Diclofenac |Misoprostol
NSAID	arthrotec 50 tablet	1140927086	M01AB55	Diclofenac |Misoprostol
NSAID	diclofenac sodium+misoprostol	1140878036	M01AB55	Diclofenac |Misoprostol
NSAID	feldene 10mg capsule	1140871672	M01AC01	Piroxicam
NSAID	brexidol 20mg tablet	1141169530	M01AC01	Piroxicam
NSAID	piroxicam	1140871666	M01AC01 |M02AA07 |S01BC06	Piroxicam
NSAID	tenoxicam	1140875346	M01AC02	Tenoxicam
NSAID	meloxicam	1140926732	M01AC06	Meloxicam
NSAID	mobic 15mg tablet	1140926796	M01AC06	Meloxicam
NSAID	mobic 7.5mg tablet	1140926794	M01AC06	Meloxicam

Category	Medication	UK Biobank Code	ATC Code	Drug Name/ Active Ingredient(s)
NSAID	nurofen 200mg tablet	1141187776	M01AE01	Ibuprofen
NSAID	cuprofen 200mg tablet	1140871388	M01AE01	Ibuprofen
NSAID	anadin ibuprofen 200mg tablet	1141153134	M01AE01	Ibuprofen
NSAID	naprosyn 250mg tablet	1140871472	M01AE02	Naproxen
NSAID	oruvail 100 m/r capsule	1140871522	M01AE03	Ketoprofen
NSAID	ketoprofen	1140871506	M01AE03 |M02AA10	Ketoprofen
NSAID	froben 50mg tablet	1140871238	M01AE09	Flurbiprofen
NSAID	flurbiprofen	1140871236	M01AE09 |M02AA19 |R02AX01 |S01BC04	Flurbiprofen
NSAID	surgam 200mg tablet	1140871616	M01AE11	Tiaprofenic Acid
NSAID	ibuprofen+menthol 5%/3% gel	1140911748	M01AE51	Ibuprofen |Menthol
NSAID	napratec tablet combination pack	1140871638	M01AE56	Naproxen |Misoprostol
NSAID	naproxen+misoprostol	1140881612	M01AE56	Naproxen |Misoprostol
NSAID	mefenamic acid	1140871542	M01AG01	Mefenamic Acid
NSAID	ponstan 250mg capsule	1140871546	M01AG01	Mefenamic Acid
NSAID	tolfenamic acid	1140928840	M01AG02	Tolfenamic Acid
NSAID	celebrex 100mg capsule	1141176668	M01AH01	Celecoxib
NSAID	celebrex 200mg capsule	1141176670	M01AH01	Celecoxib
NSAID	etoricoxib	1141180140	M01AH05	Etoricoxib
NSAID	arcoxia 60mg tablet	1141180148	M01AH05	Etoricoxib
NSAID	arcoxia 90mg tablet	1141180150	M01AH05	Etoricoxib
NSAID	arcoxia 120mg tablet	1141180152	M01AH05	Etoricoxib
NSAID	nabumetone	1140875336	M01AX01	Nabumetone
NSAID	relifex 500mg tablet	1140875338	M01AX01	Nabumetone
NSAID	glucosamine product	1141188442	M01AX05	Glucosamine
NSAID	chondroitin product	1187	M01AX25	Chondroitin Sulfate
ANILIDES	paracetamol	2038460150	N02BE01	Acetaminophen
ANILIDES	paracetamol product	1140868240	N02BE01	Acetaminophen
ANILIDES	anadin paracetamol 500mg tablet	1141188516	N02BE01	Acetaminophen
ANILIDES	panadol 500mg tablet	1140856240	N02BE01	Acetaminophen
ANILIDES	panadol 500mg capsule	1140856238	N02BE01	Acetaminophen
ANILIDES	hedex tablet	1140911758	N02BE01	Acetaminophen
ANILIDES	paramax tablet	1140872036	N02BE51	Acetaminophen |Metoclopramide
ANILIDES	solpadeine headache soluble tablet	1141189068	N02BE51	Caffeine |Acetaminophen
ANILIDES	non-drowsy sinutab tablet	1141188512	N02BE51	Acetaminophen |Pseudoephedrine
ANILIDES	sudafed dual relief capsule	1141171672	N02BE51	Acetaminophen |Phenylephrine
ANILIDES	lemsip sachet	1140911762	N02BE51	Acetaminophen |Phenylephrine
ANILIDES	panadol extra tablet	1141168646	N02BE51	Caffeine |Acetaminophen
ANILIDES	solpadeine headache tablet	1141189066	N02BE51	Caffeine |Acetaminophen
ANILIDES	paracetamol+caffeine	1140884404	N02BE51	Caffeine |Acetaminophen
ANILIDES	midrid capsule	1140871966	N02BE51	Acetaminophen |Isometheptene
ANILIDES	paracetamol+metoclopramide 500mg/5mg tablet	1140872044	N02BE51	Acetaminophen |Metoclopramide

© 2020 Rosoff DB et al. *JAMA Psychiatry*.

Category	Medication	UK Biobank Code	ATC Code	Drug Name/ Active Ingredient(s)
SALICYLIC ACID	aspro clear maximum strength soluble tablet	1140909480	N02BA01	Acetylsalicylic Acid
SALICYLIC ACID	disprin direct dispersible tablet	1140882192	N02BA01	Acetylsalicylic Acid
SALICYLIC ACID	anadin tablet	1140911754	N02BA51	Caffeine |Acetylsalicylic Acid
SALICYLIC ACID	beechams powder	1140911760	N02BA51	Caffeine |Acetylsalicylic Acid
SALICYLIC ACID	alka-seltzer tablet	1140917114	N02BA51	Acetylsalicylic Acid |Sodium Bicarbonate |Citric Acid
SALICYLIC ACID	migramax sachet powder	1141175756	N02BA51	Acetylsalicylic Acid |Metoclopramide

**Abbreviations:**
NSAIDS: Anti-inflammatory and antirheumatic products, non-steroids
SALICYLIC ACID: Salicylic acid and derivatives
Anatomical Therapeutic Chemical Classification System

© 2020 Rosoff DB et al. *JAMA Psychiatry*.

eTable 3. Independent Instruments for Prescription Opioid Use: Harmonized Data

							EXPOSURE:				OUTCOMES:					
							OPIOID USE				MDD			ASRD		
SNP	CHR	POS	EA	OA	PAL	F	beta	eaf	se	P-value	beta	se	P-value	beta	se	P-value
rs138200696	1	117727725	C	A	F	21.95	0.199	0.016	0.042	2.80E-06	0.044	0.044	0.32	0.211	0.079	0.008
rs2618039	1	112324111	T	A	T	30.57	0.061	0.381	0.011	3.20E-08	0.007	0.009	0.47	0.007	0.018	0.68
rs945211	1	32191798	G	C	T	21.71	-0.051	0.383	0.011	3.20E-06	0.010	0.010	0.30	0.000	0.019	0.99
rs138405014	2	221947203	A	G	F	21.01	-0.218	0.013	0.048	4.60E-06	0.028	0.052	0.59	0.044	0.056	0.43
rs139152977	2	199098906	A	T	F	22.69	0.164	0.025	0.034	1.90E-06	0.010	0.030	0.74	-0.022	0.018	0.23
rs140729888	3	10521537	C	T	F	21.11	-0.205	0.015	0.045	4.30E-06	-0.087	0.036	0.01	0.010	0.019	0.58
rs521956	3	173117975	T	C	F	25.22	-0.054	0.483	0.011	5.10E-07	-0.010	0.009	0.30	-0.011	0.018	0.54
rs7428430	3	50174184	T	C	F	37.00	-0.065	0.483	0.011	1.20E-09	-0.012	0.009	0.19	-0.037	0.017	0.04
rs7631941	3	80480052	T	C	F	20.87	0.073	0.131	0.016	4.90E-06	-0.016	0.014	0.24	0.008	0.027	0.77
rs13135092	4	103198082	G	A	F	25.19	0.099	0.081	0.020	5.20E-07	-0.019	0.019	0.31	0.003	0.040	0.94
rs1397440	4	140892872	T	C	F	21.51	-0.053	0.325	0.012	3.50E-06	-0.022	0.010	0.02	0.006	0.059	0.92
rs76034781	4	3272782	A	G	F	23.52	0.098	0.077	0.020	1.20E-06	0.038	0.021	0.07	0.096	0.031	0.002
rs11743674	5	151418837	C	G	T	22.07	0.061	0.222	0.013	2.60E-06	0.007	0.011	0.53	0.010	0.021	0.64
rs56226325[b]	7	2078981	T	C	F	25.30	-0.074	0.155	0.015	4.90E-07	-0.035	0.012	0.004	-0.081	0.024	6.41E-04
rs117710046	8	59430010	T	C	F	23.64	0.210	0.016	0.043	1.20E-06	0.008	0.031	0.80	-0.024	0.056	0.68
rs1519369	8	139393909	T	C	F	21.24	-0.053	0.327	0.012	4.00E-06	-0.001	0.010	0.91	-0.015	0.018	0.41
rs12238134	9	128746612	A	G	F	37.71	-0.071	0.305	0.012	8.20E-10	-0.003	0.010	0.75	-0.023	0.019	0.24
rs17487601	9	37213641	A	G	F	24.42	-0.055	0.356	0.011	7.70E-07	-0.005	0.010	0.62	0.015	0.028	0.60
rs7860752	9	81389671	G	A	F	26.01	0.069	0.193	0.014	3.40E-07	0.014	0.011	0.23	0.044	0.021	0.04
rs12357321	10	21790476	A	G	F	29.05	0.063	0.312	0.012	7.10E-08	-0.001	0.010	0.95	0.002	0.019	0.89
rs4258296	10	134953361	C	T	F	24.76	0.056	0.363	0.011	6.50E-07	0.011	0.011	0.32	0.024	0.019	0.22
rs7110825	11	46120317	C	T	F	22.32	0.057	0.270	0.012	2.30E-06	0.014	0.010	0.18	0.011	0.019	0.56
rs4763505	12	10477712	T	A	T	21.04	-0.053	0.317	0.012	4.50E-06	-0.011	0.010	0.29	-0.037	0.020	0.06

© 2020 Rosoff DB et al. *JAMA Psychiatry*.

							EXPOSURE:				OUTCOMES:					
							OPIOID USE				MDD			ASRD		
**SNP**	**CHR**	**POS**	**EA**	**OA**	**PAL**	**F**	**beta**	**eaf**	**se**	**P-value**	**beta**	**se**	**P-value**	**beta**	**se**	**P-value**
rs56262049	13	42644705	C	T	F	22.20	-0.066	0.182	0.014	2.50E-06	-0.011	0.012	0.35	-0.026	0.022	0.25
rs7319102	13	58252801	G	A	F	22.22	0.060	0.229	0.013	2.40E-06	0.012	0.011	0.28	0.051	0.020	0.01
rs7154623	14	73798243	G	A	F	22.25	0.064	0.193	0.014	2.40E-06	-0.012	0.011	0.31	-0.030	0.022	0.18
rs113544339	15	86576076	G	T	F	21.47	-0.166	0.023	0.036	3.60E-06	-0.047	0.031	0.13	-0.067	0.061	0.27
rs2289739	15	41801512	T	G	F	22.62	0.054	0.343	0.011	2.00E-06	0.009	0.010	0.36	-0.010	0.018	0.59
rs75169822	15	75887405	G	A	F	23.40	0.059	0.262	0.012	1.30E-06	-0.010	0.010	0.33	-0.001	0.019	0.97
rs975376	16	73100503	C	G	T	23.87	0.071	0.164	0.015	1.00E-06	-0.006	0.013	0.65	0.014	0.025	0.58
rs59695806	17	43156023	C	T	F	23.80	0.057	0.294	0.012	1.10E-06	0.011	0.010	0.29	-0.011	0.019	0.57
rs2297197	20	44673546	G	C	T	24.44	0.060	0.281	0.012	7.70E-07	0.028	0.010	0.006	0.028	0.020	0.15
rs6141277	20	30831157	C	G	T	22.40	0.052	0.392	0.011	2.20E-06	0.006	0.009	0.52	0.045	0.017	0.009
rs73228014	21	42752754	A	G	F	21.38	0.161	0.024	0.035	3.80E-06	0.010	0.052	0.85	NA	NA	NA

[a] Identified as potentially invalid instrument due to reverse causation by Steiger filtering for exposure on outcome MDD.
[b] Identified as potentially invalid instrument due to reverse causation by Steiger filtering for exposure on outcome ASRD.

Abbreviations:

MDD: Major depressive disorder/ major depression outcome
ASRD: Anxiety and stress-related disorders outcome
SNP: Single nucleotide polymorphism - genetic instrument
CHR: Chromosome
POS: Position
beta: SNP effect of effect allele
se: standard error of SNP effect
P-value: P-value associated with SNP effect

N: GWAS sample size
EA: effect allele
OA: other allele
PAL: palindromic (true or false)
F: F-statistic for the instrument.

eTable 4. Independent Instruments for Salicylic Acid Use: Harmonized Data

							EXPOSURE:				OUTCOMES:					
							SALICYLIC ACID USE				MDD			ASRD		
**SNP**	**CHR**	**POS**	**EA**	**OA**	**PAL**	**F**	**beta**	**eaf**	**se**	**P-value**	**beta**	**se**	**P-value**	**beta**	**se**	**P-value**
rs10218528	1	174447588	A	T	T	25.45	-0.041	0.393	0.008	4.50E-07	-0.004	0.010	0.67	-0.004	0.018	0.83
rs11206268	1	54445348	G	A	F	22.38	-0.075	0.066	0.016	2.20E-06	-0.015	0.018	0.39	0.002	0.033	0.96
rs113242618	1	234118046	A	G	F	21.59	-0.154	0.014	0.033	3.40E-06	-0.011	0.039	0.77	0.086	0.086	0.31
rs583104	1	109821307	G	T	F	67.95	-0.078	0.225	0.009	1.70E-16	0.018	0.011	0.10	-0.010	0.020	0.61
rs4299376	2	44072576	G	T	F	28.66	0.045	0.324	0.008	8.60E-08	0.002	0.010	0.85	-0.011	0.019	0.57
rs896232	2	2732877	T	C	F	23.18	-0.040	0.361	0.008	1.50E-06	-0.020	0.010	0.04	-0.015	0.019	0.41
rs13082582	3	170202670	C	G	T	23.59	0.039	0.403	0.008	1.20E-06	-0.001	0.010	0.96	0.019	0.018	0.30
rs9865841[b]	3	135876125	A	G	F	22.31	-0.045	0.222	0.010	2.30E-06	-0.014	0.012	0.23	-0.067	0.022	0.002
rs1825548	4	20150967	T	A	T	21.31	0.055	0.124	0.012	3.90E-06	0.004	0.015	0.78	0.044	0.027	0.11
rs12188010[b]	5	166996466	A	T	T	23.60	0.040	0.376	0.008	1.20E-06	0.034	0.009	3.56E-04	0.059	0.018	8.82E-04
rs117733303	6	160922870	G	A	F	42.05	0.185	0.020	0.028	8.90E-11	0.013	0.037	0.73	-0.012	0.088	0.89
rs1931656	6	82610188	A	T	T	21.29	0.037	0.456	0.008	4.00E-06	-0.002	0.009	0.85	0.005	0.017	0.79
rs2523589	6	31327334	T	G	F	31.39	0.044	0.495	0.008	2.10E-08	0.041	0.024	0.09	0.043	0.017	0.01
rs74617384	6	160997118	T	A	T	53.41	0.105	0.083	0.014	2.70E-13	0.008	0.018	0.65	-0.005	0.033	0.88
rs56226325[b]	7	2078981	T	C	F	26.86	-0.057	0.155	0.011	2.20E-07	-0.035	0.012	0.004	-0.081	0.024	6.41E-04
rs28601761	8	126500031	G	C	T	54.84	-0.061	0.415	0.008	1.30E-13	-0.005	0.009	0.58	0.000	0.017	0.99
rs75278536	8	19821425	G	T	F	27.45	-0.067	0.106	0.013	1.60E-07	0.001	0.015	0.96	0.011	0.028	0.69
rs75786943	8	97103955	C	G	T	22.98	0.149	0.016	0.031	1.60E-06	-0.003	0.038	0.93	-0.038	0.081	0.64
rs1831733	9	22076071	C	T	F	32.96	0.046	0.483	0.008	9.40E-09	0.019	0.010	0.04	0.012	0.018	0.51
rs635634	9	136155000	T	C	F	42.40	0.067	0.185	0.010	7.40E-11	-0.004	0.012	0.71	0.030	0.022	0.16
rs10508267	10	3846959	C	G	T	22.01	-0.039	0.359	0.008	2.70E-06	0.008	0.010	0.41	-0.025	0.019	0.18
rs10887793	10	89990905	T	G	F	28.30	0.043	0.448	0.008	1.00E-07	0.002	0.011	0.88	0.023	0.019	0.24
rs59681006	11	70179401	C	T	F	22.87	-0.057	0.130	0.012	1.70E-06	0.009	0.014	0.49	0.019	0.026	0.45

© 2020 Rosoff DB et al. *JAMA Psychiatry*.

							EXPOSURE:				OUTCOMES:					
							SALICYLIC ACID USE				MDD			ASRD		
**SNP**	**CHR**	**POS**	**EA**	**OA**	**PAL**	**F**	**beta**	**eaf**	**se**	**P-value**	**beta**	**se**	**P-value**	**beta**	**se**	**P-value**
rs77507211	11	24918930	T	G	F	20.91	0.137	0.018	0.030	4.80E-06	0.034	0.044	0.43	NA	NA	NA
rs964184	11	116648917	G	C	T	29.77	0.063	0.134	0.012	4.90E-08	0.001	0.013	0.96	-0.016	0.025	0.53
rs113551213	12	12513870	A	G	F	21.21	0.085	0.050	0.018	4.10E-06	-0.012	0.024	0.61	0.075	0.048	0.12
rs12580718	12	67407656	A	G	F	22.38	-0.039	0.367	0.008	2.20E-06	-0.011	0.009	0.25	0.003	0.018	0.87
rs7315004	12	31928182	A	T	T	25.42	-0.041	0.419	0.008	4.60E-07	0.018	0.009	0.06	0.026	0.018	0.14
rs1960743	13	83832609	A	G	F	21.26	0.059	0.109	0.013	4.00E-06	-0.032	0.015	0.03	0.001	0.028	0.97
rs4995008	13	106831286	A	G	F	21.13	0.054	0.131	0.012	4.30E-06	0.007	0.013	0.60	0.017	0.026	0.49
rs950100	14	42971842	A	G	F	21.06	-0.072	0.069	0.016	4.50E-06	-0.004	0.019	0.81	0.001	0.035	0.97
rs185197153	16	58450949	C	A	F	22.12	-0.163	0.013	0.035	2.60E-06	0.007	0.035	0.85	0.005	0.017	0.79
rs11874619	18	46500936	C	G	T	21.61	-0.039	0.388	0.008	3.30E-06	-0.013	0.010	0.16	-0.014	0.018	0.43
rs7244655	18	67986087	A	G	F	22.38	-0.043	0.248	0.009	2.20E-06	-0.017	0.011	0.12	-0.010	0.020	0.62
rs73015016	19	11191300	A	G	F	60.77	-0.096	0.118	0.012	6.40E-15	-0.012	0.015	0.40	-0.016	0.028	0.58
rs7412	19	45412079	T	C	F	56.77	-0.111	0.078	0.015	4.90E-14	-0.002	0.019	0.94	-0.010	0.031	0.74
rs117571676	20	21002369	A	T	T	21.78	-0.133	0.020	0.029	3.10E-06	0.003	0.033	0.92	0.015	0.057	0.80
rs9982261	21	17528955	A	G	F	21.62	-0.175	0.011	0.038	3.30E-06	0.035	0.051	0.49	NA	NA	NA
rs139696858	22	47836888	A	G	F	23.05	-0.164	0.014	0.034	1.60E-06	0.018	0.044	0.69	NA	NA	NA
rs9612563	22	24597540	C	A	F	21.92	-0.080	0.058	0.017	2.80E-06	0.001	0.019	0.94	-0.027	0.037	0.47

[a] Identified as potentially invalid instrument due to reverse causation by Steiger filtering for exposure on outcome MDD.
[b] Identified as potentially invalid instrument due to reverse causation by Steiger filtering for exposure on outcome ASRD.

**Abbreviations:**
MDD: Major depressive disorder/ major depression outcome<br>ASRD: Anxiety and stress-related disorders outcome<br>SNP: Single nucleotide polymorphism - genetic instrument<br>CHR: Chromosome<br>POS: Position<br>beta: SNP effect<br>se: standard error of SNP effect<br>P-value: P-value associated with SNP effect
N: GWAS sample size<br>EA: effect allele

© 2020 Rosoff DB et al. *JAMA Psychiatry*.

OA: other allele
PAL: palindromic (true or false)
F: F-statistic for the instrument.

© 2020 Rosoff DB et al. *JAMA Psychiatry*.

eTable 5. Independent Instruments for Anilides Use: Harmonized Data

							EXPOSURE:				OUTCOMES:					
							ANILIDE USE				ASRD			MDD		
**SNP**	**CHR**	**POS**	**EA**	**OA**	**PAL**	**F**	**beta**	**eaf**	**se**	**P-value**	**beta**	**se**	**P-value**	**beta**	**se**	**P-value**
rs10158289	1	174050667	G	A	F	24.58	-0.033	0.450	0.007	7.10E-07	-0.012	0.018	0.51	-0.005	0.009	0.60
rs1050316	1	156434703	G	T	F	27.27	0.036	0.348	0.007	1.80E-07	-0.028	0.018	0.13	-0.009	0.010	0.35
rs11807461	1	153832768	A	T	T	22.58	-0.060	0.073	0.013	2.00E-06	0.035	0.037	0.34	0.015	0.019	0.43
rs1476901	1	187760345	C	T	F	21.62	-0.031	0.456	0.007	3.30E-06	-0.013	0.018	0.46	-0.004	0.009	0.71
rs41272663	2	211302627	A	C	F	22.21	-0.035	0.262	0.007	2.40E-06	-0.005	0.021	0.79	0.002	0.011	0.81
rs62180637	2	217424243	C	T	F	22.76	-0.031	0.466	0.007	1.80E-06	0.006	0.018	0.72	-0.020	0.009	0.03
rs685139	2	234758539	C	T	F	25.97	-0.061	0.080	0.012	3.50E-07	0.053	0.030	0.08	0.019	0.016	0.23
rs74335824	2	242452616	T	C	F	20.96	0.041	0.166	0.009	4.70E-06	-0.020	0.025	0.41	0.004	0.013	0.79
rs1264194	3	48625266	T	C	F	21.32	0.034	0.284	0.007	3.90E-06	-0.012	0.020	0.53	0.006	0.011	0.58
rs4356969	4	112302592	C	G	T	23.32	-0.036	0.264	0.008	1.40E-06	-0.039	0.021	0.06	-0.013	0.011	0.24
rs62305543	4	87217713	C	T	F	23.85	0.033	0.376	0.007	1.00E-06	-0.013	0.018	0.47	-0.005	0.009	0.57
rs62315620	4	67719607	T	C	F	21.52	0.035	0.255	0.008	3.50E-06	0.003	0.021	0.89	0.006	0.011	0.54
rs62378567	5	173106225	G	A	F	23.87	-0.116	0.019	0.024	1.00E-06	0.024	0.080	0.77	0.037	0.038	0.34
rs11242905	6	474405	C	T	F	21.14	0.045	0.127	0.010	4.30E-06	-0.033	0.026	0.21	-0.011	0.014	0.42
rs4236040	6	25761209	T	C	F	22.27	-0.049	0.111	0.010	2.40E-06	-0.039	0.029	0.18	-0.038	0.016	0.02
rs6456469	6	22107290	G	A	F	21.95	0.031	0.455	0.007	2.80E-06	0.025	0.018	0.16	-0.005	0.009	0.62
rs9399923[b]	6	96850379	G	A	F	20.93	0.036	0.219	0.008	4.80E-06	0.072	0.021	4.64E-04	0.007	0.011	0.55
rs4415205	7	67214927	A	G	F	21.76	0.041	0.172	0.009	3.10E-06	-0.016	0.023	0.49	0.004	0.012	0.74
rs73168813	7	135846513	C	G	NA	22.49	0.106	0.022	0.022	2.10E-06	NA	NA	NA	-0.052	0.045	0.25
rs4741328	9	13764507	C	T	F	23.74	-0.033	0.368	0.007	1.10E-06	0.019	0.018	0.30	-0.008	0.010	0.43
rs73531210	9	112519447	T	C	F	22.14	-0.070	0.051	0.015	2.50E-06	0.001	0.039	0.98	0.006	0.020	0.74
rs1648372	11	115893928	T	G	F	22.04	-0.046	0.126	0.010	2.70E-06	-0.016	0.030	0.59	-0.029	0.015	0.05
rs725957	12	57331741	A	G	F	32.43	0.039	0.395	0.007	1.20E-08	0.008	0.018	0.64	0.009	0.009	0.32

© 2020 Rosoff DB et al. *JAMA Psychiatry*.

							EXPOSURE:				OUTCOMES:					
							ANILIDE USE				ASRD			MDD		
SNP	CHR	POS	EA	OA	PAL	F	beta	eaf	se	P-value	beta	se	P-value	beta	se	P-value
rs1413124	13	59210253	C	G	T	20.95	0.035	0.247	0.008	4.70E-06	0.023	0.020	0.24	0.006	0.010	0.58
rs9571576	13	66936133	C	T	F	23.60	0.032	0.494	0.007	1.20E-06	0.024	0.017	0.16	0.013	0.009	0.16
rs17128721	14	93462039	A	C	F	22.46	0.066	0.060	0.014	2.20E-06	0.023	0.039	0.56	-0.016	0.020	0.44
rs2803966	14	73100786	C	T	F	21.07	-0.030	0.472	0.007	4.40E-06	0.017	0.017	0.32	0.006	0.009	0.49
rs12905100	15	74601813	G	A	F	21.66	-0.032	0.363	0.007	3.30E-06	0.004	0.018	0.84	0.002	0.010	0.80
rs2947009[b]	15	46300580	A	T	T	21.55	0.031	0.490	0.007	3.40E-06	0.051	0.019	0.006	0.009	0.010	0.33
rs80114555	16	54597818	A	G	F	21.02	-0.096	0.025	0.021	4.50E-06	-0.059	0.077	0.45	-0.063	0.034	0.06
rs9302806	16	5793163	A	G	F	22.74	0.031	0.424	0.007	1.90E-06	-0.005	0.017	0.78	0.004	0.009	0.63
rs2239936	17	10535349	C	G	T	23.89	0.041	0.185	0.008	1.00E-06	0.016	0.022	0.47	0.011	0.012	0.36
rs73998427	17	70840296	T	C	F	21.90	0.088	0.031	0.019	2.90E-06	-0.005	0.056	0.93	0.039	0.027	0.15
rs1261097	18	52868977	G	T	F	23.80	0.035	0.300	0.007	1.10E-06	0.028	0.019	0.13	0.023	0.010	0.02
rs6141314	20	31093514	A	G	F	22.60	0.037	0.242	0.008	2.00E-06	0.035	0.020	0.08	0.011	0.011	0.29
rs4271845	21	20164265	C	G	T	21.87	0.031	0.433	0.007	2.90E-06	0.020	0.017	0.26	0.019	0.009	0.04

[a] Identified as potentially invalid instrument due to reverse causation by Steiger filtering for exposure on outcome MDD.
[b] Identified as potentially invalid instrument due to reverse causation by Steiger filtering for exposure on outcome ASRD.

Abbreviations:

MDD: Major depressive disorder/ major depression outcome
ASRD: Anxiety and stress-related disorders outcome
SNP: Single nucleotide polymorphism - genetic instrument
CHR: Chromosome
POS: Position
beta: SNP effect
se: standard error of SNP effect
P-value: P-value associated with SNP effect

N: GWAS sample size
EA: effect allele
OA: other allele
PAL: palindromic (true or false)
F: F-statistic for the instrument.

© 2020 Rosoff DB et al. *JAMA Psychiatry*.

eTable 6. Independent Instruments for NSAID Use: Harmonized Data

							EXPOSURE:				OUTCOMES:					
							NSAID USE				MDD			ASRD		
SNP	CHR	POS	EA	OA	PAL	F	beta	eaf	se	P-value	beta	se	P-value	beta	se	P-value
rs12409445	1	192403191	C	T	F	24.66	-0.045	0.189	0.009	6.80E-07	-0.013	0.012	0.26	0.001	0.022	0.95
rs1632360	1	154267672	G	A	F	27.01	0.079	0.055	0.015	2.00E-07	0.026	0.022	0.25	0.032	0.048	0.51
rs3737240	1	150483355	T	C	F	27.28	-0.037	0.395	0.007	1.80E-07	0.003	0.009	0.77	-0.013	0.017	0.46
rs4110942	1	29337802	G	A	F	21.31	-0.056	0.088	0.012	3.90E-06	0.007	0.016	0.67	0.016	0.032	0.61
rs7532754	1	115824515	T	C	F	22.85	-0.034	0.360	0.007	1.70E-06	-0.014	0.010	0.16	0.005	0.019	0.78
rs3821269	2	70698590	A	G	F	29.39	0.038	0.498	0.007	5.90E-08	0.015	0.009	0.10	0.036	0.017	0.04
rs11097493	4	97020614	A	T	T	22.33	0.034	0.361	0.007	2.30E-06	-0.009	0.009	0.32	-0.007	0.018	0.68
rs151415	4	103137539	G	C	T	26.98	0.043	0.237	0.008	2.10E-07	-0.001	0.011	0.94	-0.017	0.021	0.40
rs56166763	4	140943169	C	G	T	32.53	-0.041	0.369	0.007	1.20E-08	-0.024	0.009	0.01	-0.018	0.018	0.31
rs7687973	4	81139104	C	T	F	28.38	-0.044	0.224	0.008	1.00E-07	-0.016	0.011	0.14	-0.018	0.021	0.39
rs12522598	5	120174466	G	A	F	35.20	0.045	0.289	0.008	3.00E-09	0.015	0.010	0.13	0.046	0.019	0.02
rs359431	5	173288534	C	T	F	27.84	0.037	0.437	0.007	1.30E-07	-0.003	0.009	0.71	0.006	0.017	0.72
rs6891880	5	149972794	G	A	F	29.74	-0.038	0.427	0.007	4.90E-08	0.017	0.009	0.07	0.000	0.018	>0.99
rs114212906	6	31391389	T	C	F	31.80	0.092	0.047	0.016	1.70E-08	-0.002	0.022	0.93	-0.029	0.040	0.47
rs1589738	6	147967761	A	G	F	21.45	0.033	0.368	0.007	3.60E-06	0.016	0.009	0.09	0.005	0.018	0.77
rs2237257	6	112005126	G	T	F	23.79	-0.052	0.123	0.011	1.10E-06	0.019	0.014	0.17	0.025	0.025	0.32
rs11993835	8	88867707	A	G	F	22.88	-0.049	0.134	0.010	1.70E-06	-0.009	0.014	0.50	0.004	0.027	0.88
rs2608029	8	129170126	G	C	T	24.25	0.036	0.334	0.007	8.50E-07	0.022	0.010	0.02	0.010	0.019	0.61
rs35990522	9	119274481	A	T	T	22.94	0.063	0.075	0.013	1.70E-06	-0.011	0.019	0.54	0.024	0.038	0.53
rs71510292	9	91898790	C	T	F	25.13	0.082	0.048	0.016	5.40E-07	-0.010	0.026	0.70	0.039	0.044	0.37
rs7856204	9	95173814	C	G	T	22.84	0.034	0.364	0.007	1.80E-06	-0.008	0.010	0.42	-0.015	0.018	0.40
rs113041162	10	71106964	A	G	F	26.10	0.050	0.147	0.010	3.20E-07	-0.010	0.013	0.44	0.010	0.025	0.68
rs11597763	10	98244903	G	A	F	25.14	0.043	0.203	0.009	5.30E-07	0.003	0.012	0.81	-0.003	0.022	0.90

© 2020 Rosoff DB et al. *JAMA Psychiatry*.

							EXPOSURE:				OUTCOMES:					
							NSAID USE				MDD			ASRD		
SNP	CHR	POS	EA	OA	PAL	F	beta	eaf	se	P-value	beta	se	P-value	beta	se	P-value
rs12285912	11	126727315	G	A	F	22.86	0.103	0.026	0.022	1.70E-06	0.019	0.028	0.49	-0.010	0.058	0.86
rs1544861	11	10679441	T	C	F	24.72	-0.037	0.333	0.007	6.60E-07	-0.010	0.010	0.29	-0.018	0.018	0.32
rs11171710	12	56368078	A	G	F	23.26	0.034	0.447	0.007	1.40E-06	-0.004	0.010	0.70	0.000	0.018	0.99
rs1458612	12	75483802	T	C	F	21.41	-0.035	0.303	0.008	3.70E-06	0.005	0.010	0.62	-0.038	0.019	0.05
rs3001426	12	57509055	C	T	F	64.37	-0.056	0.453	0.007	1.00E-15	-0.010	0.009	0.27	-0.031	0.018	0.08
rs3809218	12	9066613	C	T	F	21.04	-0.034	0.335	0.007	4.50E-06	-0.010	0.010	0.29	-0.019	0.018	0.29
rs17081175	13	66919700	A	C	F	23.48	-0.038	0.265	0.008	1.30E-06	-0.011	0.011	0.31	0.001	0.021	0.96
rs56044130	14	94821812	G	A	F	24.05	0.058	0.095	0.012	9.40E-07	0.023	0.016	0.17	0.025	0.033	0.45
rs8013531	14	23247687	T	C	F	23.25	0.034	0.405	0.007	1.40E-06	-0.001	0.010	0.94	NA	NA	NA
rs34862454	15	75101530	C	T	F	29.97	-0.040	0.330	0.007	4.40E-08	0.003	0.010	0.73	0.022	0.019	0.24
rs10164103	18	65419274	G	A	F	24.36	-0.058	0.097	0.012	8.00E-07	-0.019	0.015	0.20	-0.006	0.030	0.85
rs144713500	19	5158138	A	G	F	21.02	0.140	0.013	0.031	4.50E-06	0.015	0.048	0.76	NA	NA	NA

[a] Identified as potentially invalid instrument due to reverse causation by Steiger filtering for exposure on outcome MDD.
[b] Identified as potentially invalid instrument due to reverse causation by Steiger filtering for exposure on outcome ASRD.

**Abbreviations:**
MDD: Major depressive disorder/ major depression outcome
ASRD: Anxiety and stress-related disorders outcome
SNP: Single nucleotide polymorphism - genetic instrument
CHR: Chromosome
POS: Position
beta: SNP effect
se: standard error of SNP effect
P-value: P-value associated with SNP effect
N: GWAS sample size

© 2020 Rosoff DB et al. *JAMA Psychiatry*.

eTable 7. Independent Instruments for Anxiety and Stress Related Disorder: Harmonized Data

							EXPOSURE			OUTCOMES:							
							ASRD			OPIOID USE				NSAID USE			
**SNP**	**CHR**	**POS**	**EA**	**OA**	**PAL**	**F**	**beta**	**se**	**P-value**	**beta**	**eaf**	**se**	**P-value**	**beta**	**eaf**	**se**	**P-value**
rs11122148	1	6848505	A	C	F	21.83	-0.113	0.024	2.85E-06	-0.015	0.156	0.015	0.16	0.010	0.156	0.010	0.15
rs12726220	1	154171550	A	G	F	22.53	0.217	0.046	2.10E-06	-0.019	0.945	0.024	0.21	-0.008	0.946	0.015	0.30
rs4655809	1	66389097	T	C	F	31.80	-0.103	0.018	1.60E-08	-0.032	0.361	0.011	0.003	-0.010	0.363	0.007	0.10
rs6425515	1	179021232	A	G	F	21.64	0.084	0.018	3.38E-06	-0.002	0.663	0.011	0.42	0.004	0.664	0.007	0.28
rs698763	2	44573624	A	G	F	20.92	0.090	0.020	4.65E-06	-0.009	0.753	0.012	0.23	-0.016	0.753	0.008	0.03
rs79928194	2	233649290	T	C	F	23.55	-0.148	0.031	1.26E-06	0.002	0.904	0.018	0.45	-0.007	0.905	0.012	0.28
rs11131050	3	6733772	T	C	F	20.94	-0.081	0.018	4.59E-06	0.002	0.535	0.011	0.44	-0.003	0.535	0.007	0.32
rs153248	5	83469915	T	C	F	22.16	0.082	0.017	2.34E-06	0.000	0.483	0.011	0.49	-0.011	0.483	0.007	0.06
rs2451828	5	7448796	T	C	F	24.55	0.293	0.059	7.37E-07	-0.027	0.021	0.037	0.23	0.016	0.022	0.024	0.25
rs118050449	7	67517974	T	G	F	22.01	0.270	0.058	2.76E-06	0.026	0.016	0.043	0.27	0.010	0.016	0.027	0.35
rs73035954	7	3563804	T	C	F	21.27	-0.093	0.020	4.19E-06	-0.039	0.246	0.012	9.43E-04	-0.026	0.246	0.008	5.76E-04
rs16916239	8	87643741	A	G	F	24.05	-0.102	0.021	8.96E-07	-0.013	0.796	0.013	0.16	-0.004	0.797	0.009	0.33
rs7041651	9	25183143	C	G	T	21.59	0.121	0.026	3.33E-06	0.005	0.878	0.016	0.37	-0.013	0.877	0.011	0.11
rs17141926	11	81021839	T	C	F	22.77	-0.094	0.020	1.92E-06	0.012	0.739	0.012	0.17	0.001	0.739	0.008	0.44
rs2263551	15	40979543	A	G	F	21.31	0.079	0.017	4.01E-06	0.001	0.506	0.011	0.47	0.000	0.505	0.007	0.48
rs4780239	15	29852093	A	G	F	21.49	-0.113	0.024	3.74E-06	0.005	0.773	0.013	0.34	0.013	0.775	0.008	0.05
rs113545947	19	32632463	T	C	F	20.89	-0.191	0.042	4.87E-06	-0.015	0.957	0.027	0.29	-0.028	0.957	0.017	0.05
rs6030245	20	41070559	T	C	F	25.36	0.113	0.023	5.06E-07	0.014	0.781	0.013	0.15	-0.006	0.781	0.008	0.23

© 2020 Rosoff DB et al. *JAMA Psychiatry*.

							EXPOSURE:			OUTCOMES:							
							ASRD			SALICYLIC ACID/ DERIVATIVES USE				ANILIDE USE			
**SNP**	**CHR**	**POS**	**EA**	**OA**	**PAL**	**F**	**beta**	**se**	**P-value**	**beta**	**eaf**	**se**	**P-value**	**beta**	**eaf**	**se**	**P-value**
rs11122148	1	6848505	A	C	F	21.83	-0.113	0.024	2.85E-06	-0.011	0.156	0.011	0.32	-0.002	0.156	0.009	0.86
rs12726220	1	154171550	A	G	F	22.53	0.217	0.046	2.10E-06	-0.004	0.946	0.017	0.80	-0.015	0.945	0.014	0.29
rs4655809	1	66389097	T	C	F	31.80	-0.103	0.018	1.60E-08	NA	NA	NA	NA	-0.011	0.361	0.007	0.12
rs6425515	1	179021232	A	G	F	21.64	0.084	0.018	3.38E-06	0.004	0.663	0.008	0.62	0.017	0.664	0.007	0.02
rs698763	2	44573624	A	G	F	20.92	0.090	0.020	4.65E-06	NA	NA	NA	NA	-0.011	0.753	0.008	0.15
rs79928194	2	233649290	T	C	F	23.55	-0.148	0.031	1.26E-06	-0.017	0.904	0.014	0.20	-0.001	0.904	0.011	0.96
rs11131050	3	6733772	T	C	F	20.94	-0.081	0.018	4.59E-06	NA	NA	NA	NA	0.002	0.536	0.007	0.81
rs153248	5	83469915	T	C	F	22.16	0.082	0.017	2.34E-06	NA	NA	NA	NA	-0.012	0.482	0.007	0.07
rs2451828	5	7448796	T	C	F	24.55	0.293	0.059	7.37E-07	-0.023	0.021	0.028	0.39	-0.006	0.022	0.023	0.78
rs118050449	7	67517974	T	G	F	22.01	0.270	0.058	2.76E-06	0.030	0.016	0.031	0.33	0.019	0.016	0.026	0.46
rs73035954	7	3563804	T	C	F	21.27	-0.093	0.020	4.19E-06	NA	NA	NA	NA	-0.017	0.246	0.008	0.02
rs16916239	8	87643741	A	G	F	24.05	-0.102	0.021	8.96E-07	-0.002	0.797	0.010	0.86	0.000	0.798	0.008	0.99
rs7041651	9	25183143	C	G	T	21.59	0.121	0.026	3.33E-06	NA	NA	NA	NA	0.002	0.878	0.010	0.82
rs17141926	11	81021839	T	C	F	22.77	-0.094	0.020	1.92E-06	NA	NA	NA	NA	0.003	0.738	0.007	0.70
rs2263551	15	40979543	A	G	F	21.31	0.079	0.017	4.01E-06	NA	NA	NA	NA	0.010	0.506	0.007	0.13
rs4780239	15	29852093	A	G	F	21.49	-0.113	0.024	3.74E-06	-0.008	0.773	0.009	0.41	0.011	0.774	0.008	0.18
rs113545947	19	32632463	T	C	F	20.89	-0.191	0.042	4.87E-06	-0.009	0.957	0.020	0.65	-0.012	0.957	0.016	0.47
rs6030245	20	41070559	T	C	F	25.36	0.113	0.023	5.06E-07	0.010	0.780	0.010	0.31	-0.001	0.780	0.008	0.95

[a] Identified as potentially invalid instrument due to reverse causation by Steiger filtering for exposure on outcome MDD
[b] Identified as potentially invalid instrument due to reverse causation by Steiger filtering for exposure on outcome ASRD

**Abbreviations:**
MDD: Major depressive disorder/ major depression outcome
ASRD: Anxiety and stress-related disorders outcome
SNP: Single nucleotide polymorphism - genetic instrument
CHR: Chromosome
POS: Position
beta: SNP effect

© 2020 Rosoff DB et al. *JAMA Psychiatry*.

se: standard error of SNP effect
P-value: P-value associated with SNP effect
N: GWAS sample size<br>F: F statistic

© 2020 Rosoff DB et al. *JAMA Psychiatry*.

eTable 8. Independent Instruments for Major Depressive Disorder: Harmonized Data

							EXPOSURE:			OUTCOMES:							
							MDD			NSAID USE				ANILIDE USE			
SNP	CHR	POS	EA	OA	PAL	F	effect	se	P-value	effect	eaf	se	P-value	effect	eaf	se	P-value
rs11205624	1	49867034	T	C	F	21.03	-0.027	0.006	4.89E-06	0.004	0.772	0.008	0.30	-0.013	0.771	0.008	0.09
rs1432639	1	72813218	A	C	F	60.84	0.039	0.005	4.55E-15	0.004	0.604	0.007	0.28	0.000	0.603	0.007	0.94
rs159963	1	8504421	A	C	F	30.37	-0.027	0.005	3.19E-08	-0.012	0.584	0.007	0.05	-0.006	0.585	0.007	0.33
rs2102341[a]	1	37193908	T	C	F	28.72	0.028	0.005	6.28E-08	0.019	0.291	0.008	0.006	0.015	0.292	0.007	0.04
rs2332571	1	181625702	T	C	F	28.63	0.031	0.006	1.08E-07	-0.023	0.225	0.008	0.003	-0.016	0.227	0.008	0.05
rs2389016[a]	1	80799329	T	C	F	33.12	0.031	0.005	1.02E-08	0.020	0.295	0.008	0.004	0.022	0.295	0.007	0.002
rs2576241	1	217100192	A	C	F	22.97	-0.023	0.005	1.66E-06	-0.007	0.522	0.007	0.16	-0.004	0.523	0.007	0.59
rs451162	1	4834202	C	G	T	20.55	0.027	0.006	4.89E-06	-0.005	0.790	0.008	0.30	-0.002	0.791	0.008	0.76
rs4660091	1	241628430	T	C	F	25.98	-0.026	0.005	2.88E-07	0.010	0.652	0.007	0.09	-0.005	0.650	0.007	0.49
rs7543975	1	23381116	A	G	F	23.51	-0.026	0.005	1.40E-06	-0.004	0.291	0.008	0.31	0.014	0.292	0.007	0.05
rs9427672	1	197754741	A	G	F	30.63	-0.032	0.006	3.12E-08	-0.008	0.236	0.008	0.16	-0.014	0.237	0.008	0.07
rs10193609	2	58890837	A	G	F	21.69	0.033	0.007	3.16E-06	0.017	0.854	0.010	0.04	0.006	0.854	0.009	0.54
rs11674333[a]	2	22517989	A	G	F	28.23	0.026	0.005	1.04E-07	0.005	0.473	0.007	0.22	0.008	0.472	0.007	0.23
rs11682175	2	57987593	T	C	F	34.27	-0.028	0.005	4.68E-09	0.004	0.526	0.007	0.29	-0.008	0.527	0.007	0.22
rs1226412	2	157111313	T	C	F	31.67	0.033	0.006	2.38E-08	0.009	0.797	0.009	0.14	0.015	0.797	0.008	0.07
rs13032969	2	173828544	T	C	F	22.83	-0.026	0.005	1.69E-06	-0.010	0.300	0.008	0.10	-0.009	0.300	0.007	0.19
rs13414769	2	240201797	T	C	F	23.65	0.025	0.005	1.10E-06	-0.003	0.668	0.007	0.36	-0.004	0.668	0.007	0.60
rs17229439	2	144096234	T	C	F	26.66	-0.028	0.006	2.99E-07	0.003	0.740	0.008	0.34	-0.009	0.739	0.007	0.24
rs62188629[a,c,d]	2	208044470	A	G	F	26.17	0.027	0.005	3.07E-07	0.012	0.309	0.007	0.05	0.028	0.310	0.007	6.40E-05
rs7580862	2	220734528	T	C	F	24.28	0.028	0.006	7.80E-07	0.014	0.234	0.008	0.04	0.006	0.233	0.008	0.42
rs76485002	2	127342267	A	G	F	36.47	0.109	0.018	1.60E-09	-0.004	0.975	0.022	0.43	0.038	0.976	0.021	0.07
rs113689582[a]	3	108150991	T	C	F	22.53	-0.034	0.007	1.74E-06	-0.004	0.135	0.010	0.35	-0.021	0.135	0.010	0.03
rs2316896	3	56383610	T	C	F	23.36	0.038	0.008	1.41E-06	-0.002	0.888	0.011	0.44	-0.004	0.889	0.011	0.73

© 2020 Rosoff DB et al. *JAMA Psychiatry*.

							EXPOSURE:			OUTCOMES:							
							MDD			NSAID USE				ANILIDE USE			
SNP	CHR	POS	EA	OA	PAL	F	effect	se	P-value	effect	eaf	se	P-value	effect	eaf	se	P-value
rs4645169[b]	3	158908427	T	C	F	23.59	-0.024	0.005	1.39E-06	-0.026	0.406	0.007	1.16E-04	-0.006	0.405	0.007	0.36
rs73102900	3	61337306	A	G	F	26.77	-0.056	0.011	2.07E-07	0.014	0.051	0.016	0.19	-0.004	0.050	0.015	0.81
rs7430565	3	158107180	A	G	F	36.00	-0.029	0.005	2.87E-09	0.009	0.586	0.007	0.11	0.003	0.585	0.007	0.60
rs7649917	3	193289549	T	C	F	28.63	-0.031	0.006	8.91E-08	0.002	0.775	0.008	0.40	-0.001	0.774	0.008	0.93
rs9869821	3	44393136	A	G	F	29.28	0.028	0.005	6.34E-08	0.009	0.320	0.007	0.10	0.003	0.318	0.007	0.68
rs13112458	4	28085218	A	C	F	25.96	0.027	0.005	3.13E-07	0.008	0.286	0.008	0.15	0.011	0.286	0.007	0.14
rs1484144[c]	4	80217597	T	C	F	21.77	0.022	0.005	3.44E-06	0.014	0.460	0.007	0.02	0.019	0.459	0.007	0.004
rs34215985	4	42047778	C	G	T	35.05	-0.037	0.006	3.13E-09	0.000	0.205	0.009	0.49	-0.010	0.204	0.008	0.20
rs6848655	4	14928149	A	G	F	20.67	0.024	0.005	4.70E-06	0.003	0.306	0.008	0.34	-0.004	0.306	0.007	0.59
rs75990749	4	175349867	C	G	T	28.57	0.074	0.014	9.84E-08	0.003	0.046	0.016	0.42	0.006	0.046	0.016	0.68
rs77087420[a]	4	123122856	A	G	F	27.80	0.058	0.011	1.52E-07	0.023	0.945	0.015	0.06	0.017	0.945	0.014	0.23
rs11135349	5	164523472	A	C	F	37.51	-0.029	0.005	1.09E-09	0.009	0.467	0.007	0.10	0.001	0.468	0.007	0.89
rs3806843	5	140212538	T	C	F	23.19	-0.024	0.005	1.37E-06	-0.002	0.466	0.007	0.37	0.003	0.467	0.007	0.65
rs55993664	5	106603482	A	C	F	26.40	-0.037	0.007	2.62E-07	0.005	0.135	0.010	0.32	0.007	0.136	0.010	0.46
rs58141521	5	77077571	A	G	F	22.35	-0.031	0.007	2.40E-06	0.003	0.845	0.010	0.36	0.003	0.846	0.009	0.70
rs71639293[a]	5	92995013	A	G	F	25.34	0.031	0.006	6.16E-07	-0.005	0.798	0.009	0.27	0.003	0.798	0.008	0.75
rs80315381	5	50484253	T	C	F	22.01	0.026	0.006	2.76E-06	-0.002	0.720	0.008	0.41	-0.001	0.720	0.007	0.90
rs12194348	6	130552744	T	G	F	23.36	-0.026	0.005	1.20E-06	0.006	0.276	0.008	0.24	-0.006	0.276	0.007	0.40
rs13213510[a]	6	72985011	T	C	F	22.02	-0.037	0.008	2.35E-06	0.011	0.099	0.012	0.17	0.010	0.099	0.011	0.38
rs1933802[a]	6	105365891	C	G	T	28.89	-0.026	0.005	9.48E-08	-0.014	0.453	0.007	0.02	-0.015	0.454	0.007	0.03
rs4870062[a]	6	152237618	T	G	F	25.19	-0.026	0.005	4.19E-07	0.005	0.660	0.007	0.24	0.000	0.660	0.007	0.96
rs6905391[c]	6	28262686	A	G	F	41.21	-0.044	0.007	1.35E-10	-0.033	0.164	0.009	1.89E-04	-0.042	0.162	0.009	2.50E-06
rs9355502	6	165136229	A	G	F	26.71	-0.025	0.005	2.60E-07	-0.001	0.448	0.007	0.45	-0.004	0.447	0.007	0.51
rs9402472[a]	6	99566521	A	G	F	30.72	0.033	0.006	2.78E-08	0.009	0.241	0.008	0.14	0.008	0.241	0.008	0.30
rs12666117	7	109105611	A	G	F	32.59	0.027	0.005	1.35E-08	0.007	0.464	0.007	0.15	0.007	0.465	0.007	0.28

© 2020 Rosoff DB et al. *JAMA Psychiatry*.

							EXPOSURE:			OUTCOMES:							
							MDD			NSAID USE				ANILIDE USE			
SNP	CHR	POS	EA	OA	PAL	F	effect	se	P-value	effect	eaf	se	P-value	effect	eaf	se	P-value
rs1403175[a,d]	7	1964357	A	T	T	23.44	-0.031	0.006	1.14E-06	-0.013	0.163	0.009	0.09	-0.011	0.164	0.009	0.22
rs2237517	7	81827390	A	C	F	26.04	0.025	0.005	2.68E-07	-0.003	0.429	0.007	0.34	-0.001	0.428	0.007	0.92
rs62450957	7	53320318	T	C	F	21.53	0.023	0.005	3.87E-06	0.005	0.352	0.007	0.25	0.004	0.352	0.007	0.55
rs75903747	7	82730477	A	G	F	25.77	-0.040	0.008	4.38E-07	0.015	0.884	0.011	0.09	0.000	0.884	0.010	0.98
rs79631004[a]	7	24844736	T	G	F	24.73	-0.038	0.008	6.50E-07	0.012	0.880	0.011	0.13	0.010	0.879	0.010	0.35
rs4242470	8	52646298	T	G	F	27.79	-0.029	0.006	1.35E-07	-0.004	0.713	0.008	0.28	0.003	0.713	0.007	0.64
rs7017108	8	20772332	T	C	F	25.21	0.025	0.005	4.16E-07	0.016	0.420	0.007	0.01	0.020	0.420	0.007	0.002
rs11140773	9	87444058	T	C	F	26.05	-0.030	0.006	3.08E-07	0.001	0.237	0.008	0.46	0.002	0.237	0.008	0.83
rs1354115	9	2983774	A	C	F	31.72	0.028	0.005	2.37E-08	-0.013	0.630	0.007	0.04	0.003	0.630	0.007	0.62
rs4526442[a]	9	37207269	T	C	F	29.06	-0.034	0.006	7.77E-08	-0.007	0.838	0.009	0.22	-0.017	0.839	0.009	0.05
rs7856424	9	119733595	T	C	F	33.34	-0.031	0.005	8.48E-09	-0.018	0.284	0.008	0.01	-0.019	0.284	0.007	0.009
rs61867293	10	106563924	T	C	F	37.59	-0.037	0.006	6.97E-10	0.002	0.192	0.009	0.41	-0.014	0.190	0.008	0.10
rs9663959	10	70181632	T	C	F	23.07	0.032	0.007	1.78E-06	0.005	0.828	0.009	0.28	0.006	0.828	0.009	0.47
rs10895718	11	104551887	A	G	F	25.39	-0.026	0.005	4.02E-07	-0.009	0.652	0.007	0.11	-0.002	0.652	0.007	0.77
rs141769277	11	89343606	T	G	F	27.06	-0.070	0.013	2.08E-07	-0.016	0.959	0.017	0.18	-0.008	0.959	0.016	0.65
rs1526565	11	131639170	T	C	F	20.89	0.022	0.005	4.67E-06	-0.001	0.479	0.007	0.47	0.004	0.480	0.007	0.56
rs174594	11	61619829	A	C	F	25.21	-0.026	0.005	4.26E-07	0.004	0.628	0.007	0.31	0.011	0.627	0.007	0.12
rs1806153	11	31850105	T	G	F	37.44	0.036	0.006	1.18E-09	0.013	0.230	0.008	0.05	0.015	0.231	0.008	0.05
rs2412137[a]	11	3090205	A	G	F	23.23	0.024	0.005	1.81E-06	0.011	0.382	0.007	0.07	0.012	0.382	0.007	0.08
rs2509805[a,c]	11	57650796	T	C	F	27.77	0.027	0.005	1.47E-07	0.018	0.318	0.007	0.008	0.025	0.318	0.007	4.00E-04
rs2514218[a]	11	113392994	T	C	F	25.81	-0.025	0.005	4.90E-07	0.003	0.356	0.007	0.33	-0.006	0.355	0.007	0.37
rs326783	11	29633243	A	T	T	26.01	-0.026	0.005	3.27E-07	-0.014	0.642	0.007	0.03	-0.013	0.641	0.007	0.05
rs7118275[a]	11	16271678	T	C	F	24.02	0.029	0.006	1.09E-06	0.007	0.794	0.009	0.22	0.017	0.795	0.008	0.04
rs7130926	11	113496928	T	C	F	22.13	-0.033	0.007	2.22E-06	-0.003	0.858	0.010	0.37	-0.019	0.858	0.009	0.04
rs2956287[a]	12	84709216	A	G	F	21.50	-0.026	0.006	3.70E-06	-0.008	0.748	0.008	0.16	-0.011	0.748	0.008	0.16

© 2020 Rosoff DB et al. *JAMA Psychiatry*.

							EXPOSURE:			OUTCOMES:							
							MDD			NSAID USE				ANILIDE USE			
SNP	CHR	POS	EA	OA	PAL	F	effect	se	P-value	effect	eaf	se	P-value	effect	eaf	se	P-value
rs4074723[a]	12	23947737	A	C	F	30.37	-0.027	0.005	3.12E-08	-0.010	0.411	0.007	0.09	-0.002	0.411	0.007	0.77
rs58235352	12	121186246	A	G	F	27.92	-0.058	0.011	1.29E-07	-0.009	0.053	0.015	0.28	0.008	0.054	0.015	0.59
rs12552	13	53625781	A	G	F	79.87	0.043	0.005	6.07E-19	0.014	0.436	0.007	0.03	0.005	0.436	0.007	0.46
rs548886	13	75792799	A	C	F	25.79	0.026	0.005	3.19E-07	0.011	0.343	0.007	0.07	0.010	0.343	0.007	0.13
rs574593	13	27118279	T	C	F	26.66	-0.028	0.006	2.11E-07	0.006	0.271	0.008	0.22	-0.009	0.271	0.007	0.22
rs9592461[a,b,c]	13	66941792	A	G	F	27.14	0.025	0.005	1.77E-07	0.031	0.480	0.007	2.80E-06	0.030	0.480	0.007	5.40E-06
rs10149470	14	104017953	A	G	F	35.02	-0.029	0.005	3.05E-09	-0.005	0.486	0.007	0.25	-0.008	0.486	0.007	0.20
rs12882789	14	32467041	A	G	F	21.95	0.025	0.005	2.89E-06	-0.001	0.728	0.008	0.43	0.003	0.728	0.007	0.67
rs2005864	14	75377692	T	C	F	33.12	0.028	0.005	6.73E-09	0.003	0.438	0.007	0.36	0.013	0.438	0.007	0.04
rs4904738[a,c,d]	14	42179732	T	C	F	34.79	-0.029	0.005	2.57E-09	-0.018	0.572	0.007	0.005	-0.025	0.572	0.007	1.60E-04
rs915057	14	64686207	A	G	F	37.47	-0.030	0.005	7.61E-10	-0.004	0.433	0.007	0.27	-0.009	0.433	0.007	0.20
rs12440728	15	36405461	A	T	T	25.62	0.025	0.005	3.93E-07	-0.007	0.404	0.007	0.17	-0.002	0.404	0.007	0.74
rs281279[a]	15	47660902	T	C	F	24.85	0.033	0.007	5.78E-07	-0.002	0.150	0.010	0.41	0.012	0.149	0.009	0.19
rs4542627[a]	15	78070743	A	T	T	23.59	-0.024	0.005	1.20E-06	-0.006	0.416	0.007	0.18	-0.019	0.415	0.007	0.005
rs8025231	15	37648402	A	C	F	49.87	-0.034	0.005	2.36E-12	-0.006	0.552	0.007	0.21	-0.012	0.553	0.007	0.07
rs11643192	16	72214276	A	C	F	30.37	0.027	0.005	3.36E-08	0.009	0.390	0.007	0.11	0.003	0.388	0.007	0.69
rs11649444[a]	16	83435153	C	G	T	21.72	-0.025	0.005	3.44E-06	-0.010	0.713	0.008	0.09	-0.020	0.712	0.007	0.007
rs1542919	16	19844790	C	G	T	23.55	-0.030	0.006	1.14E-06	0.000	0.786	0.008	0.48	-0.012	0.786	0.008	0.14
rs67253370	16	52294303	A	T	T	22.33	-0.029	0.006	2.61E-06	-0.002	0.182	0.009	0.43	-0.013	0.182	0.009	0.14
rs7198928	16	7666402	T	C	F	32.27	0.028	0.005	1.00E-08	-0.010	0.617	0.007	0.08	-0.011	0.616	0.007	0.09
rs8063603	16	6310645	A	G	F	33.77	-0.031	0.005	6.87E-09	-0.006	0.681	0.008	0.20	-0.002	0.683	0.007	0.78
rs17727765	17	27576962	T	C	F	33.32	-0.051	0.009	8.51E-09	0.001	0.924	0.013	0.48	0.006	0.925	0.013	0.62
rs11663393[a]	18	50614732	A	G	F	32.19	0.028	0.005	1.65E-08	0.004	0.461	0.007	0.26	0.012	0.461	0.007	0.06
rs12958048	18	53101598	A	G	F	43.93	0.034	0.005	3.61E-11	0.007	0.335	0.007	0.16	0.008	0.335	0.007	0.27
rs62099069	18	36883737	A	T	T	32.41	-0.028	0.005	1.31E-08	0.002	0.421	0.007	0.37	0.004	0.421	0.007	0.51

© 2020 Rosoff DB et al. *JAMA Psychiatry*.

							EXPOSURE:			OUTCOMES:							
							MDD			NSAID USE				ANILIDE USE			
SNP	CHR	POS	EA	OA	PAL	F	effect	se	P-value	effect	eaf	se	P-value	effect	eaf	se	P-value
rs75344947	18	40940932	T	C	F	24.01	-0.034	0.007	1.01E-06	-0.010	0.863	0.010	0.17	-0.007	0.863	0.010	0.45
rs28567442	19	30930222	T	C	F	26.63	0.026	0.005	2.57E-07	-0.004	0.590	0.007	0.29	0.013	0.591	0.007	0.05
rs7248751	19	32447377	A	G	F	21.05	-0.023	0.005	3.99E-06	-0.006	0.322	0.007	0.21	-0.009	0.322	0.007	0.20
rs11697370[b]	20	47731767	T	C	F	21.71	-0.023	0.005	3.53E-06	0.022	0.378	0.007	0.001	0.009	0.379	0.007	0.20
rs41278104	20	39990700	T	C	F	29.55	-0.034	0.006	7.13E-08	-0.015	0.818	0.009	0.05	-0.014	0.818	0.008	0.11
rs6063085[a,c]	20	45840459	A	C	F	24.60	0.025	0.005	7.84E-07	0.009	0.625	0.007	0.09	0.029	0.627	0.007	2.10E-05
rs372519[a,d]	21	46574554	A	G	F	22.37	0.023	0.005	2.94E-06	0.011	0.538	0.007	0.06	0.013	0.537	0.007	0.05
rs2267983	22	50698157	A	C	F	28.32	-0.028	0.005	1.00E-07	-0.006	0.326	0.007	0.20	-0.014	0.326	0.007	0.05
rs415551	22	34152661	T	G	F	21.69	0.033	0.007	2.78E-06	-0.012	0.137	0.010	0.12	-0.015	0.138	0.009	0.12
rs5758265	22	41617897	A	G	F	32.97	0.031	0.005	7.55E-09	-0.003	0.286	0.008	0.36	0.018	0.285	0.007	0.01

							EXPOSURE:			OUTCOMES:							
							MDD			SALICYLIC ACID/ DERIVATIVES USE				OPIOID USE			
SNP	CHR	POS	EA	OA	PAL	F	effect	se	P-value	effect	eaf	se	P-value	effect	eaf	se	P-value
rs11205624	1	49867034	T	C	F	21.03	-0.027	0.006	4.89E-06	-0.009	0.770	0.009	0.31	-0.019	0.770	0.013	0.07
rs1432639	1	72813218	A	C	F	60.84	0.039	0.005	4.55E-15	0.003	0.603	0.008	0.70	0.006	0.603	0.011	0.30
rs159963	1	8504421	A	C	F	30.37	-0.027	0.005	3.19E-08	-0.006	0.586	0.008	0.44	-0.007	0.585	0.011	0.26
rs2102341[a]	1	37193908	T	C	F	28.72	0.028	0.005	6.28E-08	0.008	0.290	0.009	0.34	0.027	0.291	0.012	0.011
rs2332571	1	181625702	T	C	F	28.63	0.031	0.006	1.08E-07	0.005	0.227	0.010	0.59	-0.008	0.227	0.013	0.28
rs2389016[a]	1	80799329	T	C	F	33.12	0.031	0.005	1.02E-08	0.016	0.294	0.009	0.06	0.036	0.293	0.012	0.001
rs2576241	1	217100192	A	C	F	22.97	-0.023	0.005	1.66E-06	-0.011	0.522	0.008	0.18	-0.006	0.522	0.011	0.28
rs451162	1	4834202	C	G	T	20.55	0.027	0.006	4.89E-06	-0.010	0.790	0.010	0.32	0.013	0.792	0.013	0.15
rs4660091	1	241628430	T	C	F	25.98	-0.026	0.005	2.88E-07	0.010	0.652	0.008	0.24	0.004	0.650	0.011	0.35

© 2020 Rosoff DB et al. *JAMA Psychiatry*.

							EXPOSURE:			OUTCOMES:							
							MDD			SALICYLIC ACID/ DERIVATIVES USE				OPIOID USE			
SNP	CHR	POS	EA	OA	PAL	F	effect	se	P-value	effect	eaf	se	P-value	effect	eaf	se	P-value
rs7543975	1	23381116	A	G	F	23.51	-0.026	0.005	1.40E-06	0.005	0.291	0.009	0.55	0.012	0.290	0.012	0.16
rs9427672	1	197754741	A	G	F	30.63	-0.032	0.006	3.12E-08	0.005	0.239	0.009	0.56	0.002	0.238	0.013	0.44
rs10193609	2	58890837	A	G	F	21.69	0.033	0.007	3.16E-06	0.013	0.851	0.011	0.25	0.010	0.853	0.015	0.25
rs11674333[a]	2	22517989	A	G	F	28.23	0.026	0.005	1.04E-07	0.009	0.473	0.008	0.26	0.027	0.474	0.011	0.007
rs11682175	2	57987593	T	C	F	34.27	-0.028	0.005	4.68E-09	0.007	0.529	0.008	0.40	0.006	0.528	0.011	0.28
rs1226412	2	157111313	T	C	F	31.67	0.033	0.006	2.38E-08	0.005	0.796	0.010	0.64	0.008	0.795	0.013	0.28
rs13032969	2	173828544	T	C	F	22.83	-0.026	0.005	1.69E-06	-0.020	0.298	0.009	0.02	0.006	0.300	0.012	0.30
rs13414769	2	240201797	T	C	F	23.65	0.025	0.005	1.10E-06	0.000	0.668	0.008	0.98	0.001	0.668	0.011	0.48
rs17229439	2	144096234	T	C	F	26.66	-0.028	0.006	2.99E-07	-0.007	0.738	0.009	0.41	0.001	0.739	0.012	0.48
rs62188629[a,c,d]	2	208044470	A	G	F	26.17	0.027	0.005	3.07E-07	0.024	0.311	0.009	0.005	0.041	0.310	0.012	2.30E-04
rs7580862	2	220734528	T	C	F	24.28	0.028	0.006	7.80E-07	-0.004	0.232	0.009	0.70	0.015	0.234	0.013	0.12
rs76485002	2	127342267	A	G	F	36.47	0.109	0.018	1.60E-09	0.003	0.976	0.026	0.91	0.036	0.976	0.035	0.16
rs113689582[a]	3	108150991	T	C	F	22.53	-0.034	0.007	1.74E-06	-0.024	0.135	0.012	0.04	-0.026	0.135	0.016	0.04
rs2316896	3	56383610	T	C	F	23.36	0.038	0.008	1.41E-06	0.005	0.888	0.013	0.69	0.016	0.889	0.017	0.17
rs4645169[b]	3	158908427	T	C	F	23.59	-0.024	0.005	1.39E-06	-0.002	0.407	0.008	0.76	0.010	0.407	0.011	0.18
rs73102900	3	61337306	A	G	F	26.77	-0.056	0.011	2.07E-07	-0.012	0.050	0.018	0.51	-0.026	0.050	0.025	0.14
rs7430565	3	158107180	A	G	F	36.00	-0.029	0.005	2.87E-09	-0.003	0.587	0.008	0.71	0.003	0.585	0.011	0.40
rs7649917	3	193289549	T	C	F	28.63	-0.031	0.006	8.91E-08	0.000	0.774	0.009	0.96	-0.013	0.774	0.013	0.16
rs9869821	3	44393136	A	G	F	29.28	0.028	0.005	6.34E-08	-0.007	0.318	0.008	0.41	-0.001	0.318	0.011	0.46
rs13112458	4	28085218	A	C	F	25.96	0.027	0.005	3.13E-07	0.003	0.284	0.009	0.76	0.006	0.285	0.012	0.31
rs1484144[c]	4	80217597	T	C	F	21.77	0.022	0.005	3.44E-06	0.000	0.458	0.008	0.96	0.004	0.459	0.011	0.37
rs34215985	4	42047778	C	G	T	35.05	-0.037	0.006	3.13E-09	0.003	0.206	0.010	0.74	-0.001	0.206	0.013	0.48
rs6848655	4	14928149	A	G	F	20.67	0.024	0.005	4.70E-06	0.010	0.306	0.009	0.24	0.011	0.307	0.012	0.18
rs75990749	4	175349867	C	G	T	28.57	0.074	0.014	9.84E-08	-0.018	0.046	0.019	0.33	0.010	0.046	0.026	0.35
rs77087420[a]	4	123122856	A	G	F	27.80	0.058	0.011	1.52E-07	0.007	0.945	0.017	0.68	0.066	0.945	0.024	0.003

© 2020 Rosoff DB et al. *JAMA Psychiatry*.

							EXPOSURE:			OUTCOMES:							
							MDD			SALICYLIC ACID/ DERIVATIVES USE				OPIOID USE			
**SNP**	**CHR**	**POS**	**EA**	**OA**	**PAL**	**F**	**effect**	**se**	**P-value**	**effect**	**eaf**	**se**	**P-value**	**effect**	**eaf**	**se**	**P-value**
rs11135349	5	164523472	A	C	F	37.51	-0.029	0.005	1.09E-09	0.004	0.467	0.008	0.59	0.011	0.466	0.011	0.16
rs3806843	5	140212538	T	C	F	23.19	-0.024	0.005	1.37E-06	0.014	0.468	0.008	0.09	-0.004	0.466	0.011	0.35
rs55993664	5	106603482	A	C	F	26.40	-0.037	0.007	2.62E-07	0.012	0.135	0.012	0.29	-0.015	0.136	0.016	0.17
rs58141521	5	77077571	A	G	F	22.35	-0.031	0.007	2.40E-06	0.001	0.845	0.011	0.91	0.008	0.847	0.015	0.29
rs71639293[a]	5	92995013	A	G	F	25.34	0.031	0.006	6.16E-07	-0.004	0.798	0.010	0.66	0.035	0.799	0.013	0.004
rs80315381	5	50484253	T	C	F	22.01	0.026	0.006	2.76E-06	-0.001	0.721	0.009	0.95	0.006	0.721	0.012	0.31
rs12194348	6	130552744	T	G	F	23.36	-0.026	0.005	1.20E-06	0.016	0.275	0.009	0.07	-0.005	0.275	0.012	0.35
rs13213510[a]	6	72985011	T	C	F	22.02	-0.037	0.008	2.35E-06	0.006	0.100	0.013	0.63	0.031	0.100	0.018	0.05
rs1933802[a]	6	105365891	C	G	T	28.89	-0.026	0.005	9.48E-08	-0.015	0.454	0.008	0.06	-0.024	0.454	0.011	0.014
rs4870062[a]	6	152237618	T	G	F	25.19	-0.026	0.005	4.19E-07	-0.013	0.660	0.008	0.12	-0.032	0.658	0.011	0.002
rs6905391[c]	6	28262686	A	G	F	41.21	-0.044	0.007	1.35E-10	-0.019	0.165	0.011	0.07	-0.028	0.165	0.014	0.03
rs9355502	6	165136229	A	G	F	26.71	-0.025	0.005	2.60E-07	-0.010	0.448	0.008	0.20	-0.003	0.448	0.011	0.39
rs9402472[a]	6	99566521	A	G	F	30.72	0.033	0.006	2.78E-08	0.007	0.242	0.009	0.45	0.031	0.242	0.013	0.007
rs12666117	7	109105611	A	G	F	32.59	0.027	0.005	1.35E-08	0.013	0.464	0.008	0.10	0.009	0.464	0.011	0.21
rs1403175[a,d]	7	1964357	A	T	T	23.44	-0.031	0.006	1.14E-06	-0.037	0.163	0.011	5.10E-04	-0.053	0.163	0.015	1.32E-04
rs2237517	7	81827390	A	C	F	26.04	0.025	0.005	2.68E-07	-0.006	0.427	0.008	0.47	0.005	0.428	0.011	0.32
rs62450957	7	53320318	T	C	F	21.53	0.023	0.005	3.87E-06	0.001	0.352	0.008	0.91	0.008	0.352	0.011	0.23
rs75903747	7	82730477	A	G	F	25.77	-0.040	0.008	4.38E-07	0.014	0.884	0.012	0.27	0.013	0.884	0.017	0.21
rs79631004[a]	7	24844736	T	G	F	24.73	-0.038	0.008	6.50E-07	0.011	0.881	0.012	0.37	0.032	0.879	0.017	0.03
rs4242470	8	52646298	T	G	F	27.79	-0.029	0.006	1.35E-07	0.004	0.713	0.009	0.68	0.007	0.714	0.012	0.28
rs7017108	8	20772332	T	C	F	25.21	0.025	0.005	4.16E-07	0.013	0.418	0.008	0.12	0.012	0.419	0.011	0.14
rs11140773	9	87444058	T	C	F	26.05	-0.030	0.006	3.08E-07	-0.002	0.237	0.009	0.83	-0.003	0.237	0.013	0.41
rs1354115	9	2983774	A	C	F	31.72	0.028	0.005	2.37E-08	-0.005	0.630	0.008	0.57	0.010	0.632	0.011	0.17
rs4526442[a]	9	37207269	T	C	F	29.06	-0.034	0.006	7.77E-08	-0.012	0.839	0.011	0.26	-0.044	0.839	0.015	0.001
rs7856424	9	119733595	T	C	F	33.34	-0.031	0.005	8.48E-09	-0.005	0.285	0.009	0.56	-0.018	0.284	0.012	0.06

© 2020 Rosoff DB et al. *JAMA Psychiatry*.

							EXPOSURE:			OUTCOMES:							
							MDD			SALICYLIC ACID/ DERIVATIVES USE				OPIOID USE			
SNP	CHR	POS	EA	OA	PAL	F	effect	se	P-value	effect	eaf	se	P-value	effect	eaf	se	P-value
rs61867293	10	106563924	T	C	F	37.59	-0.037	0.006	6.97E-10	-0.013	0.190	0.010	0.21	-0.008	0.190	0.014	0.27
rs9663959	10	70181632	T	C	F	23.07	0.032	0.007	1.78E-06	-0.006	0.828	0.011	0.54	0.006	0.829	0.014	0.35
rs10895718	11	104551887	A	G	F	25.39	-0.026	0.005	4.02E-07	0.000	0.651	0.008	0.99	-0.005	0.650	0.011	0.34
rs141769277	11	89343606	T	G	F	27.06	-0.070	0.013	2.08E-07	-0.028	0.959	0.020	0.17	-0.023	0.959	0.027	0.20
rs1526565	11	131639170	T	C	F	20.89	0.022	0.005	4.67E-06	-0.004	0.479	0.008	0.66	-0.001	0.480	0.011	0.45
rs174594	11	61619829	A	C	F	25.21	-0.026	0.005	4.26E-07	0.019	0.626	0.008	0.018	0.019	0.624	0.011	0.05
rs1806153	11	31850105	T	G	F	37.44	0.036	0.006	1.18E-09	0.001	0.230	0.009	0.94	-0.002	0.232	0.013	0.43
rs2412137[a]	11	3090205	A	G	F	23.23	0.024	0.005	1.81E-06	0.007	0.380	0.008	0.42	0.018	0.381	0.011	0.05
rs2509805[a,c]	11	57650796	T	C	F	27.77	0.027	0.005	1.47E-07	0.013	0.319	0.009	0.14	0.037	0.319	0.012	7.32E-04
rs2514218[a]	11	113392994	T	C	F	25.81	-0.025	0.005	4.90E-07	-0.007	0.355	0.008	0.40	-0.030	0.354	0.011	0.003
rs326783	11	29633243	A	T	T	26.01	-0.026	0.005	3.27E-07	-0.015	0.642	0.008	0.08	-0.019	0.642	0.011	0.05
rs7118275[a]	11	16271678	T	C	F	24.02	0.029	0.006	1.09E-06	0.008	0.793	0.010	0.38	0.023	0.795	0.013	0.04
rs7130926	11	113496928	T	C	F	22.13	-0.033	0.007	2.22E-06	-0.020	0.857	0.011	0.09	-0.016	0.858	0.015	0.15
rs2956287[a]	12	84709216	A	G	F	21.50	-0.026	0.006	3.70E-06	-0.019	0.748	0.009	0.03	-0.030	0.747	0.012	0.007
rs4074723[a]	12	23947737	A	C	F	30.37	-0.027	0.005	3.12E-08	0.000	0.410	0.008	0.97	-0.026	0.409	0.011	0.009
rs58235352	12	121186246	A	G	F	27.92	-0.058	0.011	1.29E-07	-0.023	0.053	0.018	0.19	-0.031	0.054	0.024	0.10
rs12552	13	53625781	A	G	F	79.87	0.043	0.005	6.07E-19	0.008	0.436	0.008	0.30	0.016	0.436	0.011	0.07
rs548886	13	75792799	A	C	F	25.79	0.026	0.005	3.19E-07	-0.006	0.344	0.008	0.49	0.005	0.342	0.011	0.33
rs574593	13	27118279	T	C	F	26.66	-0.028	0.006	2.11E-07	0.005	0.272	0.009	0.60	-0.011	0.271	0.012	0.19
rs9592461[a,b,c]	13	66941792	A	G	F	27.14	0.025	0.005	1.77E-07	0.012	0.478	0.008	0.14	0.030	0.479	0.011	0.003
rs10149470	14	104017953	A	G	F	35.02	-0.029	0.005	3.05E-09	-0.012	0.486	0.008	0.13	-0.017	0.488	0.011	0.06
rs12882789	14	32467041	A	G	F	21.95	0.025	0.005	2.89E-06	-0.009	0.729	0.009	0.34	0.008	0.729	0.012	0.26
rs2005864	14	75377692	T	C	F	33.12	0.028	0.005	6.73E-09	0.005	0.439	0.008	0.53	0.002	0.438	0.011	0.43
rs4904738[a,c,d]	14	42179732	T	C	F	34.79	-0.029	0.005	2.57E-09	-0.026	0.573	0.008	0.001	-0.037	0.574	0.011	3.33E-04
rs915057	14	64686207	A	G	F	37.47	-0.030	0.005	7.61E-10	0.000	0.435	0.008	0.97	0.001	0.435	0.011	0.45

© 2020 Rosoff DB et al. *JAMA Psychiatry*.

							EXPOSURE:			OUTCOMES:							
							MDD			SALICYLIC ACID/ DERIVATIVES USE				OPIOID USE			
**SNP**	**CHR**	**POS**	**EA**	**OA**	**PAL**	**F**	**effect**	**se**	**P-value**	**effect**	**eaf**	**se**	**P-value**	**effect**	**eaf**	**se**	**P-value**
rs12440728	15	36405461	A	T	T	25.62	0.025	0.005	3.93E-07	-0.014	0.407	0.008	0.08	0.013	0.408	0.011	0.12
rs281279[a]	15	47660902	T	C	F	24.85	0.033	0.007	5.78E-07	0.011	0.150	0.011	0.30	0.030	0.149	0.015	0.02
rs4542627[a]	15	78070743	A	T	T	23.59	-0.024	0.005	1.20E-06	-0.006	0.416	0.008	0.45	-0.030	0.416	0.011	0.003
rs8025231	15	37648402	A	C	F	49.87	-0.034	0.005	2.36E-12	-0.016	0.552	0.008	0.05	-0.024	0.553	0.011	0.014
rs11643192	16	72214276	A	C	F	30.37	0.027	0.005	3.36E-08	0.006	0.389	0.008	0.43	-0.001	0.389	0.011	0.45
rs11649444[a]	16	83435153	C	G	T	21.72	-0.025	0.005	3.44E-06	-0.012	0.712	0.009	0.16	-0.022	0.712	0.012	0.03
rs1542919	16	19844790	C	G	T	23.55	-0.030	0.006	1.14E-06	-0.002	0.787	0.010	0.82	-0.003	0.788	0.013	0.40
rs67253370	16	52294303	A	T	T	22.33	-0.029	0.006	2.61E-06	-0.014	0.183	0.010	0.19	-0.003	0.184	0.014	0.42
rs7198928	16	7666402	T	C	F	32.27	0.028	0.005	1.00E-08	-0.005	0.617	0.008	0.52	0.005	0.617	0.011	0.34
rs8063603	16	6310645	A	G	F	33.77	-0.031	0.005	6.87E-09	-0.008	0.682	0.009	0.37	-0.007	0.683	0.012	0.26
rs17727765	17	27576962	T	C	F	33.32	-0.051	0.009	8.51E-09	0.016	0.924	0.015	0.30	0.018	0.924	0.021	0.19
rs11663393[a]	18	50614732	A	G	F	32.19	0.028	0.005	1.65E-08	0.005	0.461	0.008	0.52	0.030	0.462	0.011	0.003
rs12958048	18	53101598	A	G	F	43.93	0.034	0.005	3.61E-11	0.001	0.333	0.008	0.93	0.013	0.335	0.011	0.13
rs62099069	18	36883737	A	T	T	32.41	-0.028	0.005	1.31E-08	0.007	0.422	0.008	0.41	-0.007	0.421	0.011	0.26
rs75344947	18	40940932	T	C	F	24.01	-0.034	0.007	1.01E-06	-0.002	0.863	0.012	0.83	-0.011	0.863	0.016	0.25
rs28567442	19	30930222	T	C	F	26.63	0.026	0.005	2.57E-07	0.009	0.590	0.008	0.27	0.006	0.591	0.011	0.29
rs7248751	19	32447377	A	G	F	21.05	-0.023	0.005	3.99E-06	0.014	0.324	0.009	0.10	-0.004	0.323	0.012	0.35
rs11697370[b]	20	47731767	T	C	F	21.71	-0.023	0.005	3.53E-06	0.000	0.378	0.008	0.96	-0.012	0.376	0.011	0.13
rs41278104	20	39990700	T	C	F	29.55	-0.034	0.006	7.13E-08	0.009	0.819	0.010	0.36	0.011	0.818	0.014	0.21
rs6063085[a,c]	20	45840459	A	C	F	24.60	0.025	0.005	7.84E-07	0.006	0.626	0.008	0.46	0.023	0.625	0.011	0.019
rs372519[a,d]	21	46574554	A	G	F	22.37	0.023	0.005	2.94E-06	0.021	0.538	0.008	0.009	0.022	0.538	0.011	0.02
rs2267983	22	50698157	A	C	F	28.32	-0.028	0.005	1.00E-07	0.005	0.327	0.008	0.56	-0.009	0.326	0.011	0.22
rs415551	22	34152661	T	G	F	21.69	0.033	0.007	2.78E-06	-0.019	0.138	0.011	0.09	-0.009	0.139	0.016	0.29
rs5758265	22	41617897	A	G	F	32.97	0.031	0.005	7.55E-09	0.004	0.285	0.009	0.61	0.010	0.285	0.012	0.19

© 2020 Rosoff DB et al. *JAMA Psychiatry*.

[a] Identified as potentially invalid instrument due to reverse causation by Steiger filtering for exposure on outcome Opioid Use.
[b] Identified as potentially invalid instrument due to reverse causation by Steiger filtering for exposure on outcome Anilide Use.
[c] Identified as potentially invalid instrument due to reverse causation by Steiger filtering for exposure on outcome NSAID Use
[d] Identified as potentially invalid instrument due to reverse causation by Steiger filtering for exposure on outcome Salicylic Acid Use.

**Abbreviations:**
MDD: Major depressive disorder/ major depression outcome
ASRD: Anxiety and stress-related disorders outcome
SNP: Single nucleotide polymorphism - genetic instrument
CHR: Chromosome
POS: Position
beta: SNP effect
se: standard error of SNP effect
P-value: P-value associated with SNP effect
N: GWAS sample size<br>F: F statistic

© 2020 Rosoff DB et al. *JAMA Psychiatry*.

eTable 9. Multivariable Instrument for Medications Use Independent Instruments for Prescription Opioid and Non-Opioid Pain Medications: Harmonized Data

					EXPOSURES:											
					OPIOID USE			NSAID USE			ANILIDE USE			SALICYLIC ACID USE		
SNP	CHR	POS	EA	OA	effect	se	P-value	effect	se	P-value	effect	se	P-value	effect	se	P-value
rs11206268	1	54445348	G	A	-0.051	0.021	0.02	-0.034	0.014	0.01	-0.049	0.013	2.20E-04	-0.075	0.016	2.20E-06
rs113242618	1	234118046	A	G	-0.161	0.045	3.30E-04	-0.119	0.029	4.20E-05	-0.041	0.028	0.14	-0.154	0.033	3.40E-06
rs12238134	1	128746612	A	G	-0.071	0.012	8.20E-10	-0.011	0.007	0.15	-0.010	0.007	0.16	-0.010	0.009	0.24
rs12409445	1	192403191	C	T	-0.029	0.014	0.04	-0.045	0.009	6.80E-07	-0.030	0.009	3.60E-04	-0.006	0.010	0.59
rs12568655	1	174434332	A	G	-0.041	0.011	2.20E-04	-0.023	0.007	0.001	-0.041	0.007	1.20E-09	-0.041	0.008	6.10E-07
rs138200696	1	117727725	C	A	0.199	0.042	2.80E-06	0.076	0.027	0.01	0.064	0.026	0.01	0.079	0.031	0.01
rs1632360	1	154267672	G	A	0.069	0.024	0.003	0.079	0.015	2.00E-07	0.055	0.014	1.20E-04	0.006	0.017	0.71
rs2430157	1	187819353	T	C	-0.024	0.011	0.03	-0.022	0.007	0.001	-0.031	0.007	2.60E-06	-0.019	0.008	0.02
rs2618039	1	112324111	T	A	0.061	0.011	3.20E-08	0.020	0.007	0.005	0.022	0.007	9.90E-04	0.027	0.008	9.40E-04
rs3737240	1	150483355	T	C	-0.034	0.011	0.002	-0.037	0.007	1.80E-07	-0.024	0.007	2.90E-04	-0.016	0.008	0.05
rs4110942	1	29337802	G	A	-0.028	0.019	0.14	-0.056	0.012	3.90E-06	-0.036	0.012	0.002	-0.012	0.014	0.39
rs945211	1	32191798	G	C	-0.051	0.011	3.20E-06	-0.006	0.007	0.37	-0.010	0.007	0.16	-0.007	0.008	0.36
rs138405014	2	221947203	A	G	-0.218	0.048	4.60E-06	0.021	0.030	0.50	-0.019	0.028	0.51	-0.076	0.035	0.03
rs139152977	2	199098906	A	T	0.164	0.034	1.90E-06	0.043	0.022	0.05	0.056	0.021	0.007	0.057	0.025	0.02
rs3821269	2	70698590	A	G	0.037	0.011	5.90E-04	0.038	0.007	5.90E-08	0.017	0.007	0.01	0.013	0.008	0.11
rs41272663	2	211302627	A	C	-0.037	0.012	0.002	-0.021	0.008	0.006	-0.035	0.007	2.40E-06	0.003	0.009	0.75
rs4299376	2	44072576	G	T	0.012	0.012	0.280	-0.018	0.007	0.02	-0.011	0.007	0.11	0.045	0.008	8.60E-08
rs4663983	2	234815005	G	A	-0.050	0.014	2.20E-04	-0.036	0.009	4.00E-05	-0.056	0.008	1.70E-11	-0.026	0.010	0.01
rs62180637	2	217424243	C	T	-0.040	0.011	1.70E-04	-0.021	0.007	0.003	-0.031	0.007	1.80E-06	-0.022	0.008	0.006
rs7567892	2	242470476	T	C	0.050	0.018	0.006	0.042	0.012	2.90E-04	0.061	0.011	2.00E-08	0.034	0.013	0.01
rs896232	2	2732877	T	C	-0.013	0.011	0.26	-0.019	0.007	0.008	-0.016	0.007	0.02	-0.040	0.008	1.50E-06

© 2020 Rosoff DB et al. *JAMA Psychiatry*.

					EXPOSURES:											
					OPIOID USE			NSAID USE			ANILIDE USE			SALICYLIC ACID USE		
**SNP**	**CHR**	**POS**	**EA**	**OA**	**effect**	**se**	**P-value**	**effect**	**se**	**P-value**	**effect**	**se**	**P-value**	**effect**	**se**	**P-value**
rs140729888	3	10521537	C	T	-0.205	0.045	4.30E-06	0.001	0.029	0.96	-0.003	0.027	0.91	-0.007	0.032	0.84
rs521956	3	173117975	T	C	-0.054	0.011	5.10E-07	-0.008	0.007	0.26	-0.021	0.007	0.001	-0.019	0.008	0.02
rs7428430	3	50174184	T	C	-0.065	0.011	1.20E-09	-0.020	0.007	0.004	-0.018	0.007	0.007	-0.023	0.008	0.003
rs7631941	3	80480052	T	C	0.073	0.016	4.90E-06	0.011	0.010	0.30	0.028	0.010	0.004	0.041	0.012	5.50E-04
rs9865841	3	135876125	A	G	-0.051	0.013	8.30E-05	-0.032	0.008	1.20E-04	-0.012	0.008	0.14	-0.045	0.010	2.30E-06
rs10033431	4	67782113	C	T	0.049	0.012	5.00E-05	0.020	0.008	0.009	0.038	0.007	4.10E-07	0.025	0.009	0.006
rs13135092	4	103198082	G	A	0.099	0.020	5.20E-07	0.052	0.013	3.80E-05	0.040	0.012	7.40E-04	0.019	0.015	0.20
rs1397440	4	140892872	T	C	-0.053	0.012	3.50E-06	-0.034	0.007	4.60E-06	-0.026	0.007	1.80E-04	-0.011	0.009	0.18
rs17449582	4	87240157	T	C	0.013	0.011	0.23	0.011	0.007	0.11	0.034	0.007	4.20E-07	0.012	0.008	0.13
rs1825548	4	20150967	T	A	0.048	0.016	0.003	0.023	0.010	0.03	0.023	0.010	0.02	0.055	0.012	3.90E-06
rs4356969	4	112302592	C	G	-0.041	0.012	8.90E-04	-0.022	0.008	0.005	-0.036	0.008	1.40E-06	-0.015	0.009	0.09
rs76034781	4	3272782	A	G	0.098	0.020	1.20E-06	0.002	0.013	0.87	0.007	0.012	0.57	0.027	0.015	0.07
rs7687973	4	81139104	C	T	-0.029	0.013	0.03	-0.044	0.008	1.00E-07	-0.024	0.008	0.002	-0.010	0.010	0.32
rs11743674	5	151418837	C	G	0.061	0.013	2.60E-06	0.009	0.008	0.28	0.016	0.008	0.05	0.027	0.009	0.005
rs12522598	5	120174466	G	A	0.033	0.012	0.006	0.045	0.008	3.00E-09	0.023	0.007	0.001	0.015	0.009	0.10
rs359431	5	173288534	C	T	0.024	0.011	0.03	0.037	0.007	1.30E-07	0.016	0.007	0.01	0.016	0.008	0.05
rs62378567	5	173106225	G	A	-0.037	0.038	0.34	-0.041	0.025	0.10	-0.116	0.024	1.00E-06	-0.046	0.028	0.11
rs6891880	5	149972794	G	A	-0.017	0.011	0.13	-0.038	0.007	4.90E-08	-0.007	0.007	0.27	-0.016	0.008	0.05
rs11242905	6	474405	C	T	0.021	0.016	0.20	0.029	0.010	0.01	0.045	0.010	4.30E-06	0.020	0.012	0.10
rs114212906	6	31391389	T	C	0.097	0.026	1.70E-04	0.092	0.016	1.70E-08	0.036	0.016	0.02	0.040	0.019	0.04
rs117733303	6	160922870	G	A	0.056	0.040	0.16	-0.042	0.026	0.10	-0.004	0.024	0.87	0.185	0.028	8.90E-11
rs1589738	6	147967761	A	G	0.021	0.011	0.06	0.033	0.007	3.60E-06	0.014	0.007	0.04	0.016	0.008	0.05
rs1931656	6	82610188	A	T	0.022	0.011	0.04	0.003	0.007	0.71	0.014	0.007	0.03	0.037	0.008	4.00E-06
rs2237257	6	112005126	G	T	-0.023	0.016	0.15	-0.052	0.011	1.10E-06	-0.018	0.010	0.07	-0.033	0.012	0.006

© 2020 Rosoff DB et al. *JAMA Psychiatry*.

					EXPOSURES:											
					OPIOID USE			NSAID USE			ANILIDE USE			SALICYLIC ACID USE		
SNP	CHR	POS	EA	OA	effect	se	P-value	effect	se	P-value	effect	se	P-value	effect	se	P-value
rs6568392	6	96861476	G	T	0.037	0.013	0.004	0.031	0.008	2.30E-04	0.040	0.008	3.60E-07	0.000	0.010	0.98
rs74617384	6	160997118	T	A	0.041	0.020	0.04	-0.042	0.013	0.001	-0.009	0.012	0.44	0.105	0.014	2.70E-13
rs4718618	7	67224778	A	G	0.026	0.014	0.06	0.025	0.009	0.005	0.040	0.008	2.70E-06	0.020	0.010	0.05
rs56226325	7	2078981	T	C	-0.074	0.015	4.90E-07	-0.021	0.010	0.03	-0.022	0.009	0.01	-0.057	0.011	2.20E-07
rs73168813	7	135846513	C	G	0.065	0.037	0.08	0.068	0.024	0.005	0.106	0.022	2.10E-06	-0.005	0.028	0.87
rs117710046	8	59430010	T	C	0.210	0.043	1.20E-06	0.035	0.028	0.21	0.072	0.026	0.006	0.072	0.031	0.02
rs11993835	8	88867707	A	G	-0.006	0.016	0.71	-0.049	0.010	1.70E-06	-0.026	0.010	0.008	-0.001	0.012	0.91
rs1519369	8	139393909	T	C	-0.053	0.012	4.00E-06	-0.010	0.007	0.17	-0.003	0.007	0.63	-0.021	0.009	0.01
rs28601761	8	126500031	G	C	-0.028	0.011	0.01	0.008	0.007	0.24	0.000	0.007	0.99	-0.061	0.008	1.30E-13
rs75278536	8	19821425	G	T	-0.044	0.017	0.01	-0.036	0.011	0.001	-0.028	0.011	0.009	-0.067	0.013	1.60E-07
rs75786943	8	97103955	C	G	0.005	0.043	0.91	0.050	0.028	0.07	0.010	0.026	0.69	0.149	0.031	1.60E-06
rs17487601	9	37213641	A	G	-0.055	0.011	7.70E-07	-0.007	0.007	0.30	-0.015	0.007	0.03	-0.009	0.008	0.29
rs4741328	9	13764507	C	T	-0.038	0.011	6.60E-04	-0.020	0.007	0.005	-0.033	0.007	1.10E-06	-0.008	0.008	0.30
rs71510292	9	91898790	C	T	0.016	0.025	0.54	0.082	0.016	5.40E-07	0.032	0.015	0.04	0.021	0.019	0.27
rs73531210	9	112519447	T	C	-0.108	0.024	8.20E-06	-0.060	0.016	1.50E-04	-0.070	0.015	2.50E-06	-0.071	0.018	8.90E-05
rs7856204	9	95173814	C	G	0.042	0.011	1.50E-04	0.034	0.007	1.80E-06	0.019	0.007	0.005	0.009	0.008	0.27
rs7860752	9	81389671	G	A	0.069	0.014	3.40E-07	0.011	0.009	0.20	0.008	0.008	0.32	-0.003	0.010	0.73
rs10508267	10	3846959	C	G	-0.018	0.011	0.11	-0.016	0.007	0.03	-0.022	0.007	0.002	-0.039	0.008	2.70E-06
rs10887793	10	89990905	T	G	0.035	0.011	0.001	0.009	0.007	0.19	0.012	0.007	0.08	0.043	0.008	1.00E-07
rs113041162	10	71106964	A	G	0.028	0.015	0.06	0.050	0.010	3.20E-07	0.035	0.009	1.80E-04	0.009	0.011	0.41
rs11597763	10	98244903	G	A	0.030	0.013	0.02	0.043	0.009	5.30E-07	0.030	0.008	2.20E-04	0.036	0.010	2.70E-04
rs12357321	10	21790476	A	G	0.063	0.012	7.10E-08	0.020	0.008	0.01	0.026	0.007	2.40E-04	0.012	0.009	0.16
rs4258296	10	134953361	C	T	0.056	0.011	6.50E-07	0.017	0.007	0.02	0.019	0.007	0.006	0.026	0.008	0.001
rs12285912	11	126727315	G	A	0.022	0.034	0.51	0.103	0.022	1.70E-06	0.057	0.021	0.005	0.034	0.025	0.18

© 2020 Rosoff DB et al. *JAMA Psychiatry*.

					EXPOSURES:											
					OPIOID USE			NSAID USE			ANILIDE USE			SALICYLIC ACID USE		
SNP	CHR	POS	EA	OA	effect	se	P-value	effect	se	P-value	effect	se	P-value	effect	se	P-value
rs1544861	11	10679441	T	C	-0.021	0.011	0.07	-0.037	0.007	6.60E-07	-0.016	0.007	0.02	-0.022	0.008	0.008
rs59681006	11	70179401	C	T	-0.002	0.016	0.91	0.000	0.010	0.97	-0.032	0.010	9.40E-04	-0.057	0.012	1.70E-06
rs7110825	11	46120317	C	T	0.057	0.012	2.30E-06	0.011	0.008	0.15	0.015	0.007	0.05	0.010	0.009	0.27
rs77507211	11	24918930	T	G	0.086	0.041	0.04	0.039	0.026	0.13	0.055	0.025	0.03	0.137	0.030	4.80E-06
rs964184	11	116648917	G	C	0.017	0.016	0.28	-0.014	0.010	0.17	-0.022	0.010	0.02	0.063	0.012	4.90E-08
rs11171710	12	56368078	A	G	0.026	0.011	0.02	0.034	0.007	1.40E-06	0.026	0.007	9.10E-05	0.025	0.008	0.002
rs11172113	12	57527283	C	T	-0.011	0.011	0.32	-0.052	0.007	1.00E-13	-0.053	0.007	8.90E-16	-0.019	0.008	0.02
rs12580718	12	67407656	A	G	-0.033	0.011	0.003	-0.026	0.007	2.90E-04	-0.013	0.007	0.05	-0.039	0.008	2.20E-06
rs1458612	12	75483802	T	C	-0.026	0.012	0.03	-0.035	0.008	3.70E-06	-0.022	0.007	0.002	-0.010	0.009	0.25
rs4763505	12	10477712	T	A	-0.053	0.012	4.50E-06	-0.021	0.007	0.004	-0.011	0.007	0.11	-0.006	0.009	0.45
rs7315004	12	31928182	A	T	-0.012	0.011	0.27	0.004	0.007	0.57	-0.007	0.007	0.28	-0.041	0.008	4.60E-07
rs1960743	13	83832609	A	G	0.023	0.017	0.18	0.029	0.011	0.009	0.023	0.011	0.03	0.059	0.013	4.00E-06
rs4995008	13	106831286	A	G	0.036	0.016	0.02	0.023	0.010	0.02	0.014	0.010	0.16	0.054	0.012	4.30E-06
rs56262049	13	42644705	C	T	-0.066	0.014	2.50E-06	-0.006	0.009	0.50	-0.013	0.009	0.14	-0.014	0.010	0.17
rs7319102	13	58252801	G	A	0.060	0.013	2.40E-06	0.018	0.008	0.03	0.022	0.008	0.006	0.024	0.009	0.01
rs9571576	13	66936133	C	T	0.030	0.011	0.006	0.033	0.007	1.80E-06	0.032	0.007	1.20E-06	0.014	0.008	0.08
rs2526922	14	73101118	A	G	-0.013	0.011	0.22	-0.022	0.007	0.002	-0.030	0.007	3.50E-06	-0.014	0.008	0.08
rs56044130	14	94821812	G	A	0.056	0.018	0.002	0.058	0.012	9.40E-07	0.035	0.011	0.002	0.022	0.014	0.10
rs7154623	14	73798243	G	A	0.064	0.014	2.40E-06	0.022	0.009	0.01	0.014	0.008	0.09	0.020	0.010	0.05
rs8013531	14	23247687	T	C	0.007	0.011	0.51	0.034	0.007	1.40E-06	0.016	0.007	0.02	0.013	0.008	0.10
rs950100	14	42971842	A	G	-0.030	0.021	0.16	-0.033	0.014	0.02	-0.026	0.013	0.04	-0.072	0.016	4.50E-06
rs113544339	15	86576076	G	T	-0.166	0.036	3.60E-06	-0.038	0.023	0.10	-0.056	0.022	0.01	-0.065	0.026	0.01
rs2289739	15	41801512	T	G	0.054	0.011	2.00E-06	0.015	0.007	0.04	0.028	0.007	4.00E-05	0.035	0.008	3.60E-05
rs75169822	15	75887405	G	A	0.059	0.012	1.30E-06	0.037	0.008	3.00E-06	0.031	0.007	3.30E-05	0.016	0.009	0.08

© 2020 Rosoff DB et al. *JAMA Psychiatry*.

					EXPOSURES:											
					OPIOID USE			NSAID USE			ANILIDE USE			SALICYLIC ACID USE		
**SNP**	**CHR**	**POS**	**EA**	**OA**	**effect**	**se**	**P-value**	**effect**	**se**	**P-value**	**effect**	**se**	**P-value**	**effect**	**se**	**P-value**
rs185197153	16	58450949	C	A	-0.082	0.046	0.08	-0.062	0.030	0.04	-0.043	0.028	0.13	-0.163	0.035	2.60E-06
rs74618578	16	54646068	G	A	-0.090	0.034	0.007	-0.062	0.022	0.004	-0.104	0.021	4.60E-07	-0.057	0.025	0.02
rs975376	16	73100503	C	G	0.071	0.015	1.00E-06	0.023	0.009	0.01	0.022	0.009	0.01	0.012	0.011	0.28
rs9925184	16	5800933	C	T	0.010	0.011	0.37	0.011	0.007	0.11	0.033	0.007	8.10E-07	0.008	0.008	0.31
rs59695806	17	43156023	C	T	0.057	0.012	1.10E-06	0.025	0.008	0.001	0.031	0.007	2.00E-05	0.019	0.009	0.03
rs73998427	17	70840296	T	C	0.089	0.031	0.004	0.054	0.020	0.006	0.088	0.019	2.90E-06	0.064	0.023	0.005
rs9915717	17	10561049	A	G	0.031	0.014	0.02	0.024	0.009	0.005	0.042	0.008	3.10E-07	0.022	0.010	0.03
rs10164103	18	65419274	G	A	-0.011	0.018	0.55	-0.058	0.012	8.00E-07	-0.041	0.011	2.20E-04	-0.021	0.013	0.12
rs1942262	18	52873317	A	G	0.043	0.012	3.50E-04	0.025	0.008	0.001	0.037	0.007	3.00E-07	0.022	0.009	0.01
rs7244655	18	67986087	A	G	-0.017	0.012	0.16	-0.022	0.008	0.006	-0.012	0.008	0.10	-0.043	0.009	2.20E-06
rs73015016	19	11191300	A	G	-0.061	0.016	2.20E-04	0.027	0.011	0.011	0.016	0.010	0.10	-0.096	0.012	6.40E-15
rs7412	19	45412079	T	C	-0.023	0.019	0.23	0.045	0.013	4.00E-04	0.045	0.012	1.90E-04	-0.111	0.015	4.90E-14
rs2297197	20	44673546	G	C	0.060	0.012	7.70E-07	0.007	0.008	0.36	0.024	0.007	0.001	0.020	0.009	0.03
rs6141277	20	30831157	C	G	0.052	0.011	2.20E-06	0.014	0.007	0.04	0.020	0.007	0.003	0.017	0.008	0.04
rs2825128	21	20172863	C	A	0.025	0.011	0.02	0.015	0.007	0.03	0.031	0.007	1.90E-06	0.016	0.008	0.04
rs73228014	21	42752754	A	G	0.161	0.035	3.80E-06	-0.007	0.023	0.75	0.031	0.021	0.14	0.016	0.026	0.54
rs139696858	22	47836888	A	G	0.016	0.045	0.73	-0.037	0.030	0.21	-0.008	0.028	0.77	-0.164	0.034	1.60E-06
rs9612563	22	24597540	C	A	-0.040	0.023	0.08	-0.014	0.015	0.33	0.011	0.014	0.42	-0.080	0.017	2.80E-06

					OUTCOMES:					
					MDD			ASRD		
**SNP**	**CHR**	**POS**	**EA**	**OA**	**effect**	**se**	**P-value**	**effect**	**se**	**P-value**
rs11206268	1	54445348	G	A	-0.015	0.018	0.39	0.002	0.033	0.96

© 2020 Rosoff DB et al. *JAMA Psychiatry*.

					OUTCOMES:					
					MDD			ASRD		
**SNP**	**CHR**	**POS**	**EA**	**OA**	**effect**	**se**	**P-value**	**effect**	**se**	**P-value**
rs113242618	1	234118046	A	G	-0.011	0.039	0.77	0.086	0.086	0.31
rs12238134	1	128746612	A	G	-0.003	0.010	0.75	-0.023	0.019	0.24
rs12409445	1	192403191	C	T	-0.013	0.012	0.26	0.001	0.022	0.95
rs12568655	1	174434332	A	G	-0.003	0.009	0.74	-0.007	0.018	0.68
rs138200696	1	117727725	C	A	0.044	0.044	0.32	NA	NA	NA
rs1632360	1	154267672	G	A	0.026	0.022	0.25	0.032	0.048	0.51
rs2430157	1	187819353	T	C	-0.005	0.009	0.57	-0.014	0.017	0.42
rs2618039	1	112324111	T	A	0.007	0.009	0.47	0.007	0.018	0.68
rs3737240	1	150483355	T	C	0.003	0.009	0.77	-0.013	0.017	0.46
rs4110942	1	29337802	G	A	0.007	0.016	0.67	0.016	0.032	0.61
rs945211	1	32191798	G	C	-0.010	0.010	0.30	0.000	0.019	0.99
rs138405014	2	221947203	A	G	0.028	0.052	0.59	0.211	0.079	0.008
rs139152977	2	199098906	A	T	0.010	0.030	0.74	0.044	0.056	0.43
rs3821269	2	70698590	A	G	0.015	0.009	0.10	0.036	0.017	0.04
rs41272663	2	211302627	A	C	0.002	0.011	0.81	-0.005	0.021	0.79
rs4299376	2	44072576	G	T	0.002	0.010	0.85	-0.011	0.019	0.57
rs4663983	2	234815005	G	A	-0.009	0.012	0.44	0.033	0.022	0.13
rs62180637	2	217424243	C	T	-0.020	0.009	0.03	0.006	0.018	0.72
rs7567892	2	242470476	T	C	0.006	0.017	0.72	-0.025	0.031	0.42
rs896232	2	2732877	T	C	-0.020	0.010	0.04	-0.015	0.019	0.41
rs140729888	3	10521537	C	T	-0.087	0.036	0.01	0.006	0.059	0.92
rs521956	3	173117975	T	C	-0.010	0.009	0.30	-0.011	0.018	0.54
rs7428430	3	50174184	T	C	-0.012	0.009	0.19	-0.037	0.017	0.04
rs7631941	3	80480052	T	C	-0.016	0.014	0.24	0.008	0.027	0.77
rs9865841	3	135876125	A	G	-0.014	0.012	0.23	-0.067	0.022	0.002

© 2020 Rosoff DB et al. *JAMA Psychiatry*.

					OUTCOMES:					
					MDD			ASRD		
SNP	CHR	POS	EA	OA	effect	se	P-value	effect	se	P-value
rs10033431	4	67782113	C	T	0.007	0.010	0.52	0.003	0.020	0.87
rs13135092	4	103198082	G	A	-0.019	0.019	0.31	0.003	0.040	0.94
rs1397440	4	140892872	T	C	-0.022	0.010	0.02	-0.022	0.018	0.23
rs17449582	4	87240157	T	C	-0.007	0.010	0.44	-0.007	0.018	0.70
rs1825548	4	20150967	T	A	0.004	0.015	0.78	0.044	0.027	0.11
rs4356969	4	112302592	C	G	-0.013	0.011	0.24	-0.039	0.021	0.06
rs76034781	4	3272782	A	G	0.038	0.021	0.07	0.096	0.031	0.002
rs7687973	4	81139104	C	T	-0.016	0.011	0.14	-0.018	0.021	0.39
rs11743674	5	151418837	C	G	0.007	0.011	0.53	0.010	0.021	0.64
rs12522598	5	120174466	G	A	0.015	0.010	0.13	0.046	0.019	0.02
rs359431	5	173288534	C	T	-0.003	0.009	0.71	0.006	0.017	0.72
rs62378567	5	173106225	G	A	0.037	0.038	0.34	0.024	0.080	0.77
rs6891880	5	149972794	G	A	0.017	0.009	0.07	0.000	0.018	>0.99
rs11242905	6	474405	C	T	-0.011	0.014	0.42	-0.033	0.026	0.21
rs114212906	6	31391389	T	C	-0.002	0.022	0.93	-0.029	0.040	0.47
rs117733303	6	160922870	G	A	0.013	0.037	0.73	-0.012	0.088	0.89
rs1589738	6	147967761	A	G	0.016	0.009	0.09	0.005	0.018	0.77
rs1931656	6	82610188	A	T	-0.002	0.009	0.85	0.005	0.017	0.79
rs2237257	6	112005126	G	T	0.019	0.014	0.17	0.025	0.025	0.32
rs6568392	6	96861476	G	T	0.015	0.011	0.16	0.057	0.021	0.006
rs74617384	6	160997118	T	A	0.008	0.018	0.65	-0.005	0.033	0.88
rs4718618	7	67224778	A	G	0.003	0.012	0.77	-0.012	0.022	0.58
rs56226325	7	2078981	T	C	-0.035	0.012	0.004	-0.081	0.024	6.41E-04
rs73168813	7	135846513	C	G	-0.052	0.045	0.25	NA	NA	NA
rs117710046	8	59430010	T	C	0.008	0.031	0.80	-0.024	0.056	0.68

© 2020 Rosoff DB et al. *JAMA Psychiatry*.

					OUTCOMES:					
					MDD			ASRD		
**SNP**	**CHR**	**POS**	**EA**	**OA**	**effect**	**se**	**P-value**	**effect**	**se**	**P-value**
rs11993835	8	88867707	A	G	-0.009	0.014	0.50	0.004	0.027	0.88
rs1519369	8	139393909	T	C	-0.001	0.010	0.91	0.010	0.019	0.58
rs28601761	8	126500031	G	C	-0.005	0.009	0.58	0.000	0.017	0.99
rs75278536	8	19821425	G	T	0.001	0.015	0.96	0.011	0.028	0.69
rs75786943	8	97103955	C	G	-0.003	0.038	0.93	-0.038	0.081	0.64
rs17487601	9	37213641	A	G	-0.005	0.010	0.62	-0.015	0.018	0.41
rs4741328	9	13764507	C	T	-0.008	0.010	0.43	0.019	0.018	0.30
rs71510292	9	91898790	C	T	-0.010	0.026	0.70	0.039	0.044	0.37
rs73531210	9	112519447	T	C	0.006	0.020	0.74	0.001	0.039	0.98
rs7856204	9	95173814	C	G	-0.008	0.010	0.42	-0.015	0.018	0.40
rs7860752	9	81389671	G	A	0.014	0.011	0.23	0.044	0.021	0.04
rs10508267	10	3846959	C	G	0.008	0.010	0.41	-0.025	0.019	0.18
rs10887793	10	89990905	T	G	0.002	0.011	0.88	0.023	0.019	0.24
rs113041162	10	71106964	A	G	-0.010	0.013	0.44	0.010	0.025	0.68
rs11597763	10	98244903	G	A	0.003	0.012	0.81	-0.003	0.022	0.90
rs12357321	10	21790476	A	G	-0.001	0.010	0.95	0.002	0.019	0.89
rs4258296	10	134953361	C	T	0.011	0.011	0.32	0.024	0.019	0.22
rs12285912	11	126727315	G	A	0.019	0.028	0.49	-0.010	0.058	0.86
rs1544861	11	10679441	T	C	-0.010	0.010	0.29	-0.018	0.018	0.32
rs59681006	11	70179401	C	T	0.009	0.014	0.49	0.019	0.026	0.45
rs7110825	11	46120317	C	T	0.014	0.010	0.18	0.011	0.019	0.56
rs77507211	11	24918930	T	G	0.034	0.044	0.43	NA	NA	NA
rs964184	11	116648917	G	C	0.001	0.013	0.96	-0.016	0.025	0.53
rs11171710	12	56368078	A	G	-0.004	0.010	0.70	0.000	0.018	0.99
rs11172113	12	57527283	C	T	-0.013	0.010	0.18	-0.026	0.017	0.14

© 2020 Rosoff DB et al. *JAMA Psychiatry*.

					OUTCOMES:					
					MDD			ASRD		
SNP	CHR	POS	EA	OA	effect	se	P-value	effect	se	P-value
rs12580718	12	67407656	A	G	-0.011	0.009	0.25	0.003	0.018	0.87
rs1458612	12	75483802	T	C	0.005	0.010	0.62	-0.038	0.019	0.05
rs4763505	12	10477712	T	A	-0.011	0.010	0.29	-0.037	0.020	0.06
rs7315004	12	31928182	A	T	0.018	0.009	0.06	0.026	0.018	0.14
rs1960743	13	83832609	A	G	-0.032	0.015	0.03	0.001	0.028	0.97
rs4995008	13	106831286	A	G	0.007	0.013	0.60	0.017	0.026	0.49
rs56262049	13	42644705	C	T	-0.011	0.012	0.35	-0.026	0.022	0.25
rs7319102	13	58252801	G	A	0.012	0.011	0.28	0.051	0.020	0.01
rs9571576	13	66936133	C	T	0.013	0.009	0.16	0.024	0.017	0.16
rs2526922	14	73101118	A	G	0.006	0.009	0.48	0.017	0.017	0.32
rs56044130	14	94821812	G	A	0.023	0.016	0.17	0.025	0.033	0.45
rs7154623	14	73798243	G	A	-0.012	0.011	0.31	-0.030	0.022	0.18
rs8013531	14	23247687	T	C	-0.001	0.010	0.94	NA	NA	NA
rs950100	14	42971842	A	G	-0.004	0.019	0.81	0.001	0.035	0.97
rs113544339	15	86576076	G	T	-0.047	0.031	0.13	-0.067	0.061	0.27
rs2289739	15	41801512	T	G	0.009	0.010	0.36	-0.010	0.018	0.59
rs75169822	15	75887405	G	A	-0.010	0.010	0.33	-0.001	0.019	0.97
rs185197153	16	58450949	C	A	0.007	0.035	0.85	NA	NA	NA
rs74618578	16	54646068	G	A	-0.066	0.034	0.05	-0.043	0.077	0.57
rs975376	16	73100503	C	G	-0.006	0.013	0.65	0.014	0.025	0.58
rs9925184	16	5800933	C	T	0.004	0.009	0.66	-0.002	0.018	0.90
rs59695806	17	43156023	C	T	0.011	0.010	0.29	-0.011	0.019	0.57
rs73998427	17	70840296	T	C	0.039	0.027	0.15	-0.005	0.056	0.93
rs9915717	17	10561049	A	G	0.007	0.012	0.56	0.005	0.022	0.84
rs10164103	18	65419274	G	A	-0.019	0.015	0.20	-0.006	0.030	0.85

© 2020 Rosoff DB et al. *JAMA Psychiatry*.

					OUTCOMES:					
					MDD			ASRD		
**SNP**	**CHR**	**POS**	**EA**	**OA**	**effect**	**se**	**P-value**	**effect**	**se**	**P-value**
rs1942262	18	52873317	A	G	0.025	0.010	0.01	0.026	0.019	0.16
rs7244655	18	67986087	A	G	-0.017	0.011	0.12	-0.010	0.020	0.62
rs73015016	19	11191300	A	G	-0.012	0.015	0.40	-0.016	0.028	0.58
rs7412	19	45412079	T	C	-0.002	0.019	0.94	-0.010	0.031	0.74
rs2297197	20	44673546	G	C	0.028	0.010	0.006	0.028	0.020	0.15
rs6141277	20	30831157	C	G	0.006	0.009	0.52	0.045	0.017	0.009
rs2825128	21	20172863	C	A	0.021	0.009	0.02	0.021	0.017	0.22
rs73228014	21	42752754	A	G	0.010	0.052	0.85	NA	NA	NA
rs139696858	22	47836888	A	G	0.018	0.044	0.69	NA	NA	NA
rs9612563	22	24597540	C	A	0.001	0.019	0.94	-0.027	0.037	0.47

**Abbreviations:**
MDD: Major depressive disorder/ major depression outcome
ASRD: Anxiety and stress-related disorders outcome
SNP: Single nucleotide polymorphism - genetic instrument
CHR: Chromosome
POS: Position
beta: SNP effect
se: standard error of SNP effect
P-value: P-value associated with SNP effect
N: GWAS sample size

	Opioid Use instrument: conventional significant effect at $P < 5\text{x}10^{-6}$	
	NSAID Use instrument: conventional significant effect at $P < 5\text{x}10^{-6}$	
	Salicylic Acid Use instrument: conventional significant effect at $P < 5\text{x}10^{-6}$	
	Anilide Use instrument: conventional significant effect at $P < 5\text{x}10^{-6}$	
	Conventionally significant at nominal $P = 0.05$ adjusted for 111 comparisons ($P < 4.50\text{x}10^{-4}$)	

© 2020 Rosoff DB et al. *JAMA Psychiatry*.

eTable 10. Multivariable Instruments for Prescription Opioid Use and Abdominal/ Stomach Pain for 3+ Months in UKB Participants on MDD and ASRD: Harmonized Data[a]

					**EXPOSURES:**								**OUTCOMES:**					
					**OPIOID USE**				**ABDOMINAL/ STOMACH PAIN FOR 3+ MONTHS**				**MDD**			**ASRD**		
**SNP**	**CHR**	**POS**	**EA**	**OA**	**eaf**	**effect**	**se**	**P-value**	**eaf**	**effect**	**se**	**P-value**	**effect**	**se**	**P-value**	**effect**	**se**	**P-value**
rs127204	1	112277693	C	A	0.400	0.059	0.011	8.50E-08	0.402	0.005	0.004	0.15	0.008	0.009	0.41	0.010	0.018	0.58
rs945211	1	32191798	G	C	0.383	-0.051	0.011	3.20E-06	0.617	0.001	0.004	0.89	-0.010	0.010	0.30	0.000	0.019	0.99
rs139152977	2	199098906	A	T	0.025	0.164	0.034	1.90E-06	0.026	-0.009	0.011	0.42	0.010	0.030	0.74	0.044	0.056	0.43
rs1437316	2	139826907	C	T	0.315	0.014	0.012	0.22	0.313	-0.018	0.004	4.80E-06	0.011	0.010	0.28	-0.017	0.019	0.35
rs72822393	2	104801697	T	C	0.033	0.034	0.030	0.27	0.031	-0.051	0.010	8.20E-07	0.027	0.025	0.28	0.077	0.047	0.10
rs13086611	3	49385417	T	A	0.303	-0.054	0.012	3.30E-06	0.304	-0.003	0.004	0.39	-0.022	0.010	0.04	0.002	0.019	0.91
rs247975	3	173107443	T	C	0.467	-0.050	0.011	3.80E-06	0.538	-0.002	0.004	0.61	-0.006	0.009	0.53	-0.001	0.017	0.97
rs7631941	3	80480052	T	C	0.131	0.073	0.016	4.90E-06	0.130	0.016	0.005	0.003	-0.016	0.014	0.24	0.008	0.027	0.77
rs13107325	4	103188709	T	C	0.074	0.100	0.020	1.10E-06	0.077	0.007	0.007	0.31	-0.027	0.020	0.17	-0.013	0.041	0.75
rs76034781	4	3272782	A	G	0.077	0.098	0.020	1.20E-06	0.078	0.013	0.007	0.05	0.038	0.021	0.07	0.096	0.031	0.002
rs769657	4	140877659	G	A	0.330	-0.053	0.011	4.80E-06	0.326	-0.005	0.004	0.15	-0.021	0.010	0.03	-0.030	0.019	0.11
rs78986576	4	77491598	T	C	0.015	0.000	0.045	>0.99	0.017	0.068	0.014	2.80E-06	-0.044	0.037	0.24	-0.012	0.069	0.87
rs116509576	5	24829795	T	C	0.034	0.022	0.030	0.47	0.040	0.043	0.009	4.20E-06	-0.042	0.028	0.14	NA	NA	NA
rs11746310	5	151391143	C	G	0.222	0.060	0.013	3.00E-06	0.222	0.004	0.004	0.42	0.008	0.011	0.48	0.011	0.021	0.61
rs2814982	6	34546560	T	C	0.102	0.082	0.018	3.20E-06	0.104	0.009	0.006	0.12	-0.008	0.015	0.57	-0.042	0.027	0.11
rs34350244	6	32561465	T	C	0.192	0.064	0.014	3.30E-06	0.196	0.002	0.005	0.68	-0.002	0.013	0.90	-0.019	0.024	0.43
rs11764780	7	2020904	C	G	0.185	-0.064	0.014	3.80E-06	0.187	0.001	0.005	0.90	-0.030	0.012	0.01	-0.063	0.022	0.004
rs117884048	8	59546371	C	T	0.015	0.213	0.044	1.50E-06	0.017	-0.026	0.014	0.07	0.008	0.030	0.79	-0.028	0.054	0.61
rs11996942	8	21674875	T	C	0.017	0.009	0.042	0.83	0.018	-0.063	0.014	2.90E-06	0.060	0.043	0.16	NA	NA	NA
rs13263652	8	139346461	C	T	0.378	-0.051	0.011	4.80E-06	0.616	0.005	0.004	0.18	-0.001	0.009	0.88	0.008	0.018	0.65
rs12554874	9	37073902	T	C	0.376	-0.053	0.011	1.60E-06	0.377	0.004	0.004	0.24	-0.006	0.009	0.50	-0.020	0.018	0.27

© 2020 Rosoff DB et al. *JAMA Psychiatry*.

					EXPOSURES:								OUTCOMES:					
					OPIOID USE				ABDOMINAL/ STOMACH PAIN FOR 3+ MONTHS				MDD			ASRD		
**SNP**	**CHR**	**POS**	**EA**	**OA**	**eaf**	**effect**	**se**	**P-value**	**eaf**	**effect**	**se**	**P-value**	**effect**	**se**	**P-value**	**effect**	**se**	**P-value**
rs13302047	9	81386827	G	C	0.192	0.069	0.014	3.60E-07	0.192	0.000	0.005	1.00	0.013	0.011	0.24	0.042	0.021	0.04
rs4837022	9	128475379	T	C	0.357	-0.054	0.011	1.40E-06	0.641	0.000	0.004	0.93	-0.001	0.010	0.96	-0.022	0.018	0.21
rs10159545	10	21766969	G	C	0.349	0.054	0.011	2.20E-06	0.355	0.000	0.004	0.91	-0.009	0.010	0.33	-0.007	0.018	0.70
rs761777	10	134938075	G	A	0.253	0.057	0.012	4.10E-06	0.259	0.005	0.004	0.20	0.029	0.012	0.02	0.055	0.022	0.01
rs1542724	11	46117183	C	A	0.269	0.057	0.012	2.70E-06	0.270	0.003	0.004	0.47	0.013	0.010	0.19	0.012	0.019	0.53
rs6484804	11	11337945	G	A	0.336	-0.007	0.012	0.53	0.335	0.019	0.004	8.70E-07	0.007	0.010	0.50	0.015	0.020	0.46
rs4763505	12	10477712	T	A	0.317	-0.053	0.012	4.50E-06	0.684	-0.002	0.004	0.65	-0.011	0.010	0.29	-0.037	0.020	0.06
rs56262049	13	42644705	C	T	0.182	-0.066	0.014	2.50E-06	0.185	0.007	0.005	0.13	-0.011	0.012	0.35	-0.026	0.022	0.25
rs7319102	13	58252801	G	A	0.229	0.060	0.013	2.40E-06	0.771	0.002	0.004	0.72	0.012	0.011	0.28	0.051	0.020	0.01
rs11624521	14	73760832	T	C	0.193	0.064	0.014	3.10E-06	0.196	0.013	0.004	0.005	-0.009	0.011	0.40	-0.020	0.022	0.37
rs112133463	15	99120809	A	G	0.090	0.017	0.019	0.37	0.091	0.028	0.006	4.50E-06	-0.005	0.016	0.77	0.010	0.031	0.76
rs113544339	15	86576076	G	T	0.023	-0.166	0.036	3.60E-06	0.024	-0.024	0.012	0.05	-0.047	0.031	0.13	-0.067	0.061	0.27
rs12594013	15	75596353	T	C	0.263	0.058	0.012	2.00E-06	0.266	-0.002	0.004	0.60	-0.009	0.011	0.41	-0.002	0.020	0.93
rs2289739	15	41801512	T	G	0.343	0.054	0.011	2.00E-06	0.348	0.003	0.004	0.49	0.009	0.010	0.36	-0.010	0.018	0.59
rs975376	16	73100503	C	G	0.164	0.071	0.015	1.00E-06	0.168	0.009	0.005	0.07	-0.006	0.013	0.65	0.014	0.025	0.58
rs9898793	17	43116313	T	C	0.261	0.056	0.012	4.20E-06	0.265	0.013	0.004	0.002	0.022	0.011	0.03	0.013	0.020	0.51
rs7359937	19	5143693	C	G	0.063	0.016	0.022	0.46	0.065	-0.034	0.007	3.60E-06	-0.004	0.019	0.83	-0.003	0.040	0.94
rs2297197	20	44673546	G	C	0.281	0.060	0.012	7.70E-07	0.290	0.004	0.004	0.35	0.028	0.010	0.01	0.028	0.020	0.15
rs6141277	20	30831157	C	G	0.392	0.052	0.011	2.20E-06	0.392	0.004	0.004	0.23	0.006	0.009	0.52	0.045	0.017	0.009
rs73228014	21	42752754	A	G	0.024	0.161	0.035	3.80E-06	0.024	0.006	0.012	0.59	0.010	0.052	0.85	-0.003	0.040	0.94

[a] For outcome phenotype MDD, summary statistics on MV instruments extracted from PGC GWAS excluding both UK Biobank (N = 29740) and 23andMe cohorts for total N = 143265 (no sample overlap).		
**Abbreviations:**		
MRC-IEU	MRC Integrative Epidemiology Unit, University of Bristol UK	
beta	effect estimate	

© 2020 Rosoff DB et al. *JAMA Psychiatry*.

se	standard error of effect estimate
SNP	single nucleotide polymorphism rsid variant identifier
CHR	chromosome
POS	base pair position
eaf	effect allele frequency
p-value	p value of effect estimate
MDD	Major depression/ major depressive disorder: outcome phenotype
ASRD	Anxiety and stress-related disorder: outcome phenotype

© 2020 Rosoff DB et al. *JAMA Psychiatry*.

eTable 11. Multivariable Instruments for Prescription Opioid Use and Neck/ Shoulder Pain for 3+ Months in UKB Participants on MDD and ASRD: Harmonized Data[a]

					EXPOSURES:								OUTCOMES:					
					OPIOID USE				NECK/ SHOULDER PAIN FOR 3+ MONTHS				MDD			ASRD		
SNP	CHR	POS	EA	OA	eaf	effect	se	P-value	eaf	effect	se	P-value	effect	se	P-value	effect	se	P-value
rs127204	1	112277693	C	A	0.400	0.059	0.011	8.50E-08	0.403	0.002	0.002	0.40	0.008	0.009	0.41	0.010	0.018	0.58
rs1391377	1	31053082	C	T	0.175	0.009	0.014	0.53	0.176	0.012	0.003	4.90E-06	-0.009	0.012	0.45	-0.013	0.022	0.56
rs138405014	2	221947203	A	G	0.013	-0.218	0.048	4.60E-06	0.014	-0.005	0.009	0.60	0.028	0.052	0.59	0.211	0.079	0.008
rs139152977	2	199098906	A	T	0.025	0.164	0.034	1.90E-06	0.027	0.010	0.006	0.10	0.010	0.030	0.74	0.044	0.056	0.43
rs2715933	2	160877502	C	T	0.204	-0.008	0.013	0.57	0.793	0.012	0.002	1.50E-06	0.005	0.011	0.65	-0.019	0.023	0.39
rs13086611	3	49385417	T	A	0.303	-0.054	0.012	3.30E-06	0.302	0.000	0.002	0.92	-0.022	0.010	0.04	0.002	0.019	0.91
rs140729888	3	10521537	C	T	0.015	-0.205	0.045	4.30E-06	0.015	-0.002	0.008	0.84	-0.087	0.036	0.01	0.006	0.059	0.92
rs17270102	3	122404806	G	A	0.075	0.000	0.020	0.99	0.076	-0.019	0.004	9.00E-07	-0.017	0.018	0.34	-0.058	0.036	0.10
rs247975	3	173107443	T	C	0.467	-0.050	0.011	3.80E-06	0.534	0.000	0.002	0.90	-0.006	0.009	0.53	-0.001	0.017	0.97
rs7631941	3	80480052	T	C	0.131	0.073	0.016	4.90E-06	0.132	0.005	0.003	0.08	-0.016	0.014	0.24	0.008	0.027	0.77
rs13107325	4	103188709	T	C	0.074	0.100	0.020	1.10E-06	0.078	0.006	0.004	0.11	-0.027	0.020	0.17	-0.013	0.041	0.75
rs16874363	4	23931209	G	T	0.276	-0.020	0.012	0.10	0.278	0.011	0.002	2.20E-06	0.002	0.010	0.85	-0.014	0.020	0.47
rs76034781	4	3272782	A	G	0.077	0.098	0.020	1.20E-06	0.078	0.001	0.004	0.73	0.038	0.021	0.07	NA	NA	NA
rs769657	4	140877659	G	A	0.330	-0.053	0.011	4.80E-06	0.328	0.002	0.002	0.40	-0.021	0.010	0.03	-0.030	0.019	0.11
rs11746310	5	151391143	C	G	0.222	0.060	0.013	3.00E-06	0.223	0.002	0.002	0.46	0.008	0.011	0.48	0.011	0.021	0.61
rs77608563	5	157581578	G	A	0.054	0.004	0.024	0.88	0.055	0.021	0.004	2.70E-06	-0.018	0.027	0.50	-0.053	0.056	0.34
rs2814982	6	34546560	T	C	0.102	0.082	0.018	3.20E-06	0.103	0.002	0.003	0.65	-0.008	0.015	0.57	-0.042	0.027	0.11
rs34350244	6	32561465	T	C	0.192	0.064	0.014	3.30E-06	0.196	0.004	0.003	0.14	-0.002	0.013	0.90	-0.019	0.024	0.43
rs11764780	7	2020904	C	G	0.185	-0.064	0.014	3.80E-06	0.184	-0.001	0.003	0.60	-0.030	0.012	0.01	-0.063	0.022	0.004
rs117710046	8	59430010	T	C	0.016	0.210	0.043	1.20E-06	0.016	0.003	0.008	0.70	0.008	0.031	0.80	-0.024	0.056	0.68
rs13263652	8	139346461	C	T	0.378	-0.051	0.011	4.80E-06	0.618	-0.001	0.002	0.79	-0.001	0.009	0.88	0.008	0.018	0.65

© 2020 Rosoff DB et al. *JAMA Psychiatry*.

					EXPOSURES:								OUTCOMES:					
					OPIOID USE				NECK/ SHOULDER PAIN FOR 3+ MONTHS				MDD			ASRD		
SNP	CHR	POS	EA	OA	eaf	effect	se	P-value	eaf	effect	se	P-value	effect	se	P-value	effect	se	P-value
rs4840542	8	10944809	G	T	0.497	0.026	0.011	0.02	0.506	0.009	0.002	4.10E-06	0.000	0.009	0.98	0.044	0.017	0.01
rs111285820	9	27991498	T	C	0.026	-0.099	0.034	0.003	0.030	-0.028	0.006	4.20E-06	0.025	0.027	0.37	-0.078	0.054	0.15
rs12554874	9	37073902	T	C	0.376	-0.053	0.011	1.60E-06	0.375	0.002	0.002	0.42	-0.006	0.009	0.50	-0.020	0.018	0.27
rs12685059	9	77549830	C	A	0.017	-0.024	0.041	0.57	0.020	0.037	0.007	6.20E-07	-0.003	0.033	0.92	0.148	0.069	0.03
rs13302047	9	81386827	G	C	0.192	0.069	0.014	3.60E-07	0.189	0.003	0.003	0.20	0.013	0.011	0.24	0.042	0.021	0.04
rs4837022	9	128475379	T	C	0.357	-0.054	0.011	1.40E-06	0.642	0.000	0.002	0.86	-0.001	0.010	0.96	-0.022	0.018	0.21
rs10159545	10	21766969	G	C	0.349	0.054	0.011	2.20E-06	0.352	0.002	0.002	0.25	-0.009	0.010	0.33	-0.007	0.018	0.70
rs117686932	10	5436486	T	C	0.018	0.051	0.041	0.22	0.024	0.032	0.007	3.50E-06	-0.027	0.037	0.46	NA	NA	NA
rs761777	10	134938075	G	A	0.253	0.057	0.012	4.10E-06	0.256	0.001	0.002	0.57	0.029	0.012	0.02	0.055	0.022	0.01
rs1542724	11	46117183	C	A	0.269	0.057	0.012	2.70E-06	0.269	0.002	0.002	0.48	0.013	0.010	0.19	0.012	0.019	0.53
rs4763505	12	10477712	T	A	0.317	-0.053	0.012	4.50E-06	0.682	-0.003	0.002	0.16	-0.011	0.010	0.29	-0.037	0.020	0.06
rs56262049	13	42644705	C	T	0.182	-0.066	0.014	2.50E-06	0.187	0.002	0.003	0.42	-0.011	0.012	0.35	-0.026	0.022	0.25
rs7319102	13	58252801	G	A	0.229	0.060	0.013	2.40E-06	0.773	0.000	0.002	0.94	0.012	0.011	0.28	0.051	0.020	0.01
rs11624521	14	73760832	T	C	0.193	0.064	0.014	3.10E-06	0.197	0.007	0.003	0.005	-0.009	0.011	0.40	-0.020	0.022	0.37
rs113544339	15	86576076	G	T	0.023	-0.166	0.036	3.60E-06	0.024	0.003	0.007	0.65	-0.047	0.031	0.13	-0.067	0.061	0.27
rs12594013	15	75596353	T	C	0.263	0.058	0.012	2.00E-06	0.266	0.001	0.002	0.74	-0.009	0.011	0.41	-0.002	0.020	0.93
rs2289739	15	41801512	T	G	0.343	0.054	0.011	2.00E-06	0.347	-0.001	0.002	0.51	0.009	0.010	0.36	-0.010	0.018	0.59
rs112512634	16	69532492	T	C	0.086	0.028	0.019	0.15	0.086	0.016	0.004	4.40E-06	0.002	0.020	0.91	NA	NA	NA
rs975376	16	73100503	C	G	0.164	0.071	0.015	1.00E-06	0.168	-0.003	0.003	0.30	-0.006	0.013	0.65	0.014	0.025	0.58
rs9898793	17	43116313	T	C	0.261	0.056	0.012	4.20E-06	0.261	0.002	0.002	0.29	0.022	0.011	0.03	0.013	0.020	0.51
rs2297197	20	44673546	G	C	0.281	0.060	0.012	7.70E-07	0.287	0.003	0.002	0.17	0.028	0.010	0.006	0.028	0.020	0.15
rs6141277	20	30831157	C	G	0.392	0.052	0.011	2.20E-06	0.393	0.002	0.002	0.28	0.006	0.009	0.52	0.045	0.017	0.009
rs232477	21	22699418	T	C	0.057	-0.009	0.023	0.69	0.059	-0.020	0.004	4.30E-06	0.012	0.020	0.55	-0.041	0.041	0.31
rs73228014	21	42752754	A	G	0.024	0.161	0.035	3.80E-06	0.024	0.010	0.007	0.15	0.010	0.052	0.85	0.096	0.031	0.002

© 2020 Rosoff DB et al. *JAMA Psychiatry*.

					EXPOSURES:								OUTCOMES:					
					OPIOID USE				NECK/ SHOULDER PAIN FOR 3+ MONTHS				MDD			ASRD		
SNP	CHR	POS	EA	OA	eaf	effect	se	P-value	eaf	effect	se	P-value	effect	se	P-value	effect	se	P-value
rs855793	22	37449608	T	C	0.137	0.006	0.016	0.70	0.862	-0.013	0.003	4.50E-06	0.028	0.014	0.05	0.017	0.028	0.54

[a] For outcome phenotype MDD, summary statistics on MV instruments extracted from PGC GWAS excluding both UK Biobank (N = 29740) and 23andMe cohorts for total N = 143265 (no sample overlap).		
**Abbreviations:**		
MRC-IEU	MRC Integrative Epidemiology Unit, University of Bristol UK	
beta	effect estimate	
se	standard error of effect estimate	
SNP	single nucleotide polymorphism rsid variant identifier	
CHR	chromosome	
POS	base pair position	
eaf	effect allele frequency	
p-value	p value of effect estimate	
MDD	Major depression/ major depressive disorder: outcome phenotype	
ASRD	Anxiety and stress-related disorder: outcome phenotype	

© 2020 Rosoff DB et al. *JAMA Psychiatry*.

eTable 12. Multivariable Instruments for Prescription Opioid Use and Pain for 3+ Months in UKB Participants on MDD and ASRD: Harmonized Data[a]

					EXPOSURES:								OUTCOMES:					
					OPIOID USE				BACK PAIN FOR 3+ MONTHS				MDD			ASRD		
SNP	**CHR**	**POS**	**EA**	**OA**	**eaf**	**effect**	**se**	**P-value**	**eaf**	**effect**	**se**	**P-value**	**effect**	**se**	**P-value**	**effect**	**se**	**P-value**
rs127204	1	112277693	C	A	0.400	0.059	0.011	8.50E-08	0.404	0.004	0.002	0.05	0.008	0.009	0.41	0.010	0.018	0.58
rs142015271	1	61346309	G	C	0.011	-0.049	0.053	0.35	0.014	0.039	0.008	3.40E-06	0.054	0.047	0.25	-0.008	0.089	0.93
rs633297	1	41985453	A	C	0.473	0.009	0.011	0.42	0.474	0.009	0.002	4.20E-06	0.010	0.009	0.28	0.009	0.018	0.63
rs72746941	1	225085775	A	G	0.056	0.004	0.023	0.88	0.057	0.021	0.004	5.00E-07	0.036	0.025	0.14	NA	NA	NA
rs80140055	1	190277915	C	T	0.030	0.047	0.032	0.14	0.032	0.027	0.006	1.50E-06	0.029	0.029	0.32	NA	NA	NA
rs945211	1	32191798	G	C	0.383	-0.051	0.011	3.20E-06	0.617	-0.001	0.002	0.48	-0.010	0.010	0.30	0.000	0.019	0.99
rs138405014	2	221947203	A	G	0.013	-0.218	0.048	4.60E-06	0.014	0.005	0.008	0.54	0.028	0.052	0.59	0.211	0.079	0.008
rs139152977	2	199098906	A	T	0.025	0.164	0.034	1.90E-06	0.027	0.002	0.006	0.72	0.010	0.030	0.74	0.044	0.056	0.43
rs72961848	2	118482463	T	C	0.144	-0.041	0.015	0.007	0.144	-0.013	0.003	4.10E-06	0.007	0.013	0.58	0.000	0.024	0.99
rs13086611	3	49385417	T	A	0.303	-0.054	0.012	3.30E-06	0.302	-0.006	0.002	0.005	-0.022	0.010	0.04	0.002	0.019	0.91
rs140729888	3	10521537	C	T	0.015	-0.205	0.045	4.30E-06	0.015	-0.012	0.008	0.13	-0.087	0.036	0.01	0.006	0.059	0.92
rs247975	3	173107443	T	C	0.467	-0.050	0.011	3.80E-06	0.534	-0.002	0.002	0.20	-0.006	0.009	0.53	-0.001	0.017	0.97
rs7631941	3	80480052	T	C	0.131	0.073	0.016	4.90E-06	0.132	0.006	0.003	0.04	-0.016	0.014	0.24	0.008	0.027	0.77
rs13107325	4	103188709	T	C	0.074	0.100	0.020	1.10E-06	0.077	0.010	0.004	0.006	-0.027	0.020	0.17	-0.013	0.041	0.75
rs144117099	4	129240996	A	G	0.018	-0.012	0.040	0.76	0.018	0.036	0.007	5.40E-07	0.001	0.043	0.98	0.015	0.074	0.84
rs76034781	4	3272782	A	G	0.077	0.098	0.020	1.20E-06	0.078	0.007	0.004	0.05	0.038	0.021	0.07	0.096	0.031	0.002
rs769657	4	140877659	G	A	0.330	-0.053	0.011	4.80E-06	0.328	-0.002	0.002	0.22	-0.021	0.010	0.03	-0.030	0.019	0.11
rs11746310	5	151391143	C	G	0.222	0.060	0.013	3.00E-06	0.223	0.001	0.002	0.71	0.008	0.011	0.48	0.011	0.021	0.61
rs2814982	6	34546560	T	C	0.102	0.082	0.018	3.20E-06	0.105	0.003	0.003	0.41	-0.008	0.015	0.57	-0.042	0.027	0.11
rs34350244	6	32561465	T	C	0.192	0.064	0.014	3.30E-06	0.194	-0.002	0.002	0.35	-0.002	0.013	0.90	-0.019	0.024	0.43
rs11764780	7	2020904	C	G	0.185	-0.064	0.014	3.80E-06	0.184	-0.002	0.002	0.54	-0.030	0.012	0.01	-0.063	0.022	0.004

© 2020 Rosoff DB et al. *JAMA Psychiatry*.

					EXPOSURES:								OUTCOMES:					
					**OPIOID USE**				**BACK PAIN FOR 3+ MONTHS**				**MDD**			**ASRD**		
SNP	**CHR**	**POS**	**EA**	**OA**	**eaf**	**effect**	**se**	**P-value**	**eaf**	**effect**	**se**	**P-value**	**effect**	**se**	**P-value**	**effect**	**se**	**P-value**
rs10112515	8	121062335	C	T	0.015	0.005	0.045	0.92	0.015	-0.036	0.008	3.60E-06	0.029	0.036	0.42	-0.088	0.080	0.27
rs117710046	8	59430010	T	C	0.016	0.210	0.043	1.20E-06	0.016	-0.002	0.008	0.83	0.008	0.031	0.80	-0.024	0.056	0.68
rs13263652	8	139346461	C	T	0.378	-0.051	0.011	4.80E-06	0.619	0.000	0.002	0.83	-0.001	0.009	0.88	0.008	0.018	0.65
rs79700578	8	119859562	C	T	0.041	-0.009	0.027	0.75	0.044	0.023	0.005	2.20E-06	-0.005	0.023	0.83	0.012	0.042	0.77
rs12554874	9	37073902	T	C	0.376	-0.053	0.011	1.60E-06	0.377	0.003	0.002	0.09	-0.006	0.009	0.50	-0.020	0.018	0.27
rs13302047	9	81386827	G	C	0.192	0.069	0.014	3.60E-07	0.190	-0.002	0.002	0.48	0.013	0.011	0.24	0.042	0.021	0.04
rs4837022	9	128475379	T	C	0.357	-0.054	0.011	1.40E-06	0.642	0.000	0.002	0.97	-0.001	0.010	0.96	-0.022	0.018	0.21
rs79657771	9	110608572	G	A	0.024	-0.068	0.035	5.20E-02	0.026	-0.028	0.006	3.80E-06	-0.020	0.029	0.49	0.123	0.057	0.03
rs10159545	10	21766969	G	C	0.349	0.054	0.011	2.20E-06	0.353	0.004	0.002	0.03	-0.009	0.010	0.33	-0.007	0.018	0.70
rs761777	10	134938075	G	A	0.253	0.057	0.012	4.10E-06	0.257	0.004	0.002	0.09	0.029	0.012	0.02	0.055	0.022	0.01
rs1542724	11	46117183	C	A	0.269	0.057	0.012	2.70E-06	0.270	0.002	0.002	0.37	0.013	0.010	0.19	0.012	0.019	0.53
rs524968	11	117368805	A	G	0.335	0.008	0.011	0.48	0.663	0.010	0.002	1.20E-06	-0.012	0.010	0.24	-0.019	0.018	0.30
rs4763505	12	10477712	T	A	0.317	-0.053	0.012	4.50E-06	0.683	0.001	0.002	0.52	-0.011	0.010	0.29	-0.037	0.020	0.06
rs56262049	13	42644705	C	T	0.182	-0.066	0.014	2.50E-06	0.185	0.000	0.002	0.84	-0.011	0.012	0.35	-0.026	0.022	0.25
rs7319102	13	58252801	G	A	0.229	0.060	0.013	2.40E-06	0.773	0.001	0.002	0.66	0.012	0.011	0.28	0.051	0.020	0.01
rs11624521	14	73760832	T	C	0.193	0.064	0.014	3.10E-06	0.197	0.006	0.002	0.01	-0.009	0.011	0.40	-0.020	0.022	0.37
rs113544339	15	86576076	G	T	0.023	-0.166	0.036	3.60E-06	0.024	-0.010	0.006	0.12	-0.047	0.031	0.13	-0.067	0.061	0.27
rs12442154	15	67241569	G	A	0.234	0.029	0.013	0.03	0.236	-0.010	0.002	3.90E-06	-0.010	0.011	0.36	-0.012	0.022	0.59
rs12594013	15	75596353	T	C	0.263	0.058	0.012	2.00E-06	0.266	0.002	0.002	0.32	-0.009	0.011	0.41	-0.002	0.020	0.93
rs2289739	15	41801512	T	G	0.343	0.054	0.011	2.00E-06	0.347	-0.001	0.002	0.78	0.009	0.010	0.36	-0.010	0.018	0.59
rs975376	16	73100503	C	G	0.164	0.071	0.015	1.00E-06	0.167	0.003	0.003	0.30	-0.006	0.013	0.65	0.014	0.025	0.58
rs9898793	17	43116313	T	C	0.261	0.056	0.012	4.20E-06	0.262	0.003	0.002	0.15	0.022	0.011	0.03	0.013	0.020	0.51
rs78328176	18	75009972	A	T	0.051	-0.027	0.025	0.27	0.058	-0.020	0.004	1.70E-06	0.009	0.024	0.71	NA	NA	NA
rs2297197	20	44673546	G	C	0.281	0.060	0.012	7.70E-07	0.288	-0.001	0.002	0.59	0.028	0.010	0.006	0.028	0.020	0.15

© 2020 Rosoff DB et al. *JAMA Psychiatry*.

					EXPOSURES:								OUTCOMES:					
					OPIOID USE				BACK PAIN FOR 3+ MONTHS				MDD			ASRD		
SNP	**CHR**	**POS**	**EA**	**OA**	**eaf**	**effect**	**se**	**P-value**	**eaf**	**effect**	**se**	**P-value**	**effect**	**se**	**P-value**	**effect**	**se**	**P-value**
rs6141277	20	30831157	C	G	0.392	0.052	0.011	2.20E-06	0.392	0.006	0.002	0.002	0.006	0.009	0.52	0.045	0.017	0.009
rs73228014	21	42752754	A	G	0.024	0.161	0.035	3.80E-06	0.024	-0.002	0.006	0.73	0.010	0.052	0.85	NA	NA	NA

[a] For outcome phenotype MDD, summary statistics on MV instruments extracted from PGC GWAS excluding both UK Biobank (N = 29740) and 23andMe cohorts for total N = 143265 (no sample overlap).	
**Abbreviations:**	
MRC-IEU	MRC Integrative Epidemiology Unit, University of Bristol UK
beta	effect estimate
se	standard error of effect estimate
SNP	single nucleotide polymorphism rsid variant identifier
CHR	chromosome
POS	base pair position
eaf	effect allele frequency
p-value	p value of effect estimate
MDD	Major depression/ major depressive disorder: outcome phenotype
ASRD	Anxiety and stress-related disorder: outcome phenotype

© 2020 Rosoff DB et al. *JAMA Psychiatry*.

eTable 13. Multivariable Instruments for Prescription Opioid Use and Knee Pain for 3+ Months in UKB Participants on MDD and ASRD: Harmonized Data[a]

					EXPOSURES:								OUTCOMES:					
					OPIOID USE				KNEE PAIN FOR 3 + MONTHS				MDD			ASRD		
**SNP**	**CHR**	**POS**	**EA**	**OA**	**eaf**	**effect**	**se**	**P-value**	**eaf**	**effect**	**se**	**P-value**	**effect**	**se**	**P-value**	**effect**	**se**	**P-value**
rs127204	1	112277693	C	A	0.400	0.059	0.011	8.50E-08	0.403	0.000	0.002	0.81	0.008	0.009	0.41	0.010	0.018	0.58
rs945211	1	32191798	G	C	0.383	-0.051	0.011	3.20E-06	0.617	0.000	0.002	0.81	-0.010	0.010	0.30	0.000	0.019	0.99
rs11886592	2	114010048	T	C	0.022	0.009	0.037	0.81	0.024	-0.028	0.006	3.70E-06	0.036	0.029	0.21	-0.001	0.063	0.99
rs138405014	2	221947203	A	G	0.013	-0.218	0.048	4.60E-06	0.014	-0.003	0.008	0.72	0.028	0.052	0.59	0.211	0.079	0.008
rs139152977	2	199098906	A	T	0.025	0.164	0.034	1.90E-06	0.027	0.002	0.006	0.78	0.010	0.030	0.74	0.044	0.056	0.43
rs67230931	2	57714037	G	A	0.309	0.019	0.012	0.10	0.310	0.010	0.002	1.50E-06	-0.021	0.010	0.03	-0.006	0.018	0.74
rs13086611	3	49385417	T	A	0.303	-0.054	0.012	3.30E-06	0.302	-0.001	0.002	0.63	-0.022	0.010	0.04	0.002	0.019	0.91
rs140729888	3	10521537	C	T	0.015	-0.205	0.045	4.30E-06	0.015	-0.002	0.008	0.76	-0.087	0.036	0.01	0.006	0.059	0.92
rs247975	3	173107443	T	C	0.467	-0.050	0.011	3.80E-06	0.536	-0.004	0.002	0.06	-0.006	0.009	0.53	-0.001	0.017	0.97
rs7631941	3	80480052	T	C	0.131	0.073	0.016	4.90E-06	0.133	0.004	0.003	0.13	-0.016	0.014	0.24	0.008	0.027	0.77
rs13107325	4	103188709	T	C	0.074	0.100	0.020	1.10E-06	0.078	0.004	0.003	0.29	-0.027	0.020	0.17	-0.013	0.041	0.75
rs75984500	4	15671383	T	C	0.064	0.031	0.022	0.16	0.070	0.017	0.004	3.80E-06	0.020	0.019	0.30	0.090	0.037	0.01
rs76034781	4	3272782	A	G	0.077	0.098	0.020	1.20E-06	0.079	0.000	0.003	0.94	0.038	0.021	0.07	0.096	0.031	0.002
rs769657	4	140877659	G	A	0.330	-0.053	0.011	4.80E-06	0.328	-0.004	0.002	0.06	-0.021	0.010	0.03	-0.030	0.019	0.11
rs11746310	5	151391143	C	G	0.222	0.060	0.013	3.00E-06	0.223	-0.001	0.002	0.78	0.008	0.011	0.48	0.011	0.021	0.61
rs2814982	6	34546560	T	C	0.102	0.082	0.018	3.20E-06	0.104	0.003	0.003	0.35	-0.008	0.015	0.57	-0.042	0.027	0.11
rs34350244	6	32561465	T	C	0.192	0.064	0.014	3.30E-06	0.196	0.002	0.002	0.36	-0.002	0.013	0.90	-0.019	0.024	0.43
rs10230736	7	150496104	A	G	0.198	0.003	0.014	0.82	0.202	-0.011	0.002	4.70E-06	0.001	0.012	0.92	0.011	0.022	0.62
rs116893232	7	113665542	T	C	0.019	-0.029	0.039	0.47	0.019	0.032	0.007	2.30E-06	-0.006	0.042	0.89	-0.089	0.084	0.29
rs11764780	7	2020904	C	G	0.185	-0.064	0.014	3.80E-06	0.185	-0.005	0.002	0.03	-0.030	0.012	0.01	-0.063	0.022	0.004
rs117710046	8	59430010	T	C	0.016	0.210	0.043	1.20E-06	0.016	0.007	0.007	0.37	0.008	0.031	0.80	-0.024	0.056	0.68

© 2020 Rosoff DB et al. *JAMA Psychiatry*.

					EXPOSURES:								OUTCOMES:					
					OPIOID USE				KNEE PAIN FOR 3 + MONTHS				MDD			ASRD		
**SNP**	**CHR**	**POS**	**EA**	**OA**	**eaf**	**effect**	**se**	**P-value**	**eaf**	**effect**	**se**	**P-value**	**effect**	**se**	**P-value**	**effect**	**se**	**P-value**
rs13263652	8	139346461	C	T	0.378	-0.051	0.011	4.80E-06	0.621	0.001	0.002	0.46	-0.001	0.009	0.88	0.008	0.018	0.65
rs12554874	9	37073902	T	C	0.376	-0.053	0.011	1.60E-06	0.374	0.000	0.002	0.81	-0.006	0.009	0.50	-0.020	0.018	0.27
rs13302047	9	81386827	G	C	0.192	0.069	0.014	3.60E-07	0.190	0.001	0.002	0.55	0.013	0.011	0.24	0.042	0.021	0.04
rs147463499	9	34990871	T	C	0.011	-0.003	0.052	0.95	0.011	-0.042	0.009	2.70E-06	-0.017	0.040	0.67	-0.004	0.085	0.96
rs4837022	9	128475379	T	C	0.357	-0.054	0.011	1.40E-06	0.642	0.000	0.002	0.82	-0.001	0.010	0.96	-0.022	0.018	0.21
rs10159545	10	21766969	G	C	0.349	0.054	0.011	2.20E-06	0.352	0.003	0.002	0.14	-0.009	0.010	0.33	-0.007	0.018	0.70
rs761777	10	134938075	G	A	0.253	0.057	0.012	4.10E-06	0.257	0.000	0.002	0.96	0.029	0.012	0.02	0.055	0.022	0.01
rs1542724	11	46117183	C	A	0.269	0.057	0.012	2.70E-06	0.271	0.002	0.002	0.40	0.013	0.010	0.19	0.012	0.019	0.53
rs4763505	12	10477712	T	A	0.317	-0.053	0.012	4.50E-06	0.683	0.002	0.002	0.44	-0.011	0.010	0.29	-0.037	0.020	0.06
rs56262049	13	42644705	C	T	0.182	-0.066	0.014	2.50E-06	0.186	0.002	0.002	0.51	-0.011	0.012	0.35	-0.026	0.022	0.25
rs7319102	13	58252801	G	A	0.229	0.060	0.013	2.40E-06	0.772	0.001	0.002	0.61	0.012	0.011	0.28	0.051	0.020	0.01
rs11624521	14	73760832	T	C	0.193	0.064	0.014	3.10E-06	0.197	0.003	0.002	0.17	-0.009	0.011	0.40	-0.020	0.022	0.37
rs113544339	15	86576076	G	T	0.023	-0.166	0.036	3.60E-06	0.024	-0.009	0.006	0.13	-0.047	0.031	0.13	-0.067	0.061	0.27
rs12594013	15	75596353	T	C	0.263	0.058	0.012	2.00E-06	0.267	0.001	0.002	0.61	-0.009	0.011	0.41	-0.002	0.020	0.93
rs2289739	15	41801512	T	G	0.343	0.054	0.011	2.00E-06	0.348	0.000	0.002	0.83	0.009	0.010	0.36	-0.010	0.018	0.59
rs975376	16	73100503	C	G	0.164	0.071	0.015	1.00E-06	0.169	0.006	0.002	0.03	-0.006	0.013	0.65	0.014	0.025	0.58
rs9898793	17	43116313	T	C	0.261	0.056	0.012	4.20E-06	0.262	0.003	0.002	0.17	0.022	0.011	0.03	0.013	0.020	0.51
rs2297197	20	44673546	G	C	0.281	0.060	0.012	7.70E-07	0.288	-0.003	0.002	0.14	0.028	0.010	0.006	0.028	0.020	0.15
rs6141277	20	30831157	C	G	0.392	0.052	0.011	2.20E-06	0.393	0.000	0.002	0.85	0.006	0.009	0.52	0.045	0.017	0.00
rs73228014	21	42752754	A	G	0.024	0.161	0.035	3.80E-06	0.023	-0.006	0.006	0.34	0.010	0.052	0.85	NA	NA	NA

[a] For outcome phenotype MDD, summary statistics on MV instruments extracted from PGC GWAS excluding both UK Biobank (N = 29740) and 23andMe cohorts for total N = 143265 (no sample overlap).

Abbreviations:

MRC-IEU	MRC Integrative Epidemiology Unit, University of Bristol UK

© 2020 Rosoff DB et al. *JAMA Psychiatry*.

beta	effect estimate	
se	standard error of effect estimate	
SNP	single nucleotide polymorphism rsid variant identifier	
CHR	chromosome	
POS	base pair position	
eaf	effect allele frequency	
p-value	p value of effect estimate	
MDD	Major depression/ major depressive disorder: outcome phenotype	
ASRD	Anxiety and stress-related disorder: outcome phenotype	

© 2020 Rosoff DB et al. *JAMA Psychiatry*.

eTable 14. Multivariable Instruments for Prescription Opioid Use and Headache Pain for 3+ Months in UKB Participants on MDD and ASRD: Harmonized Data[a]

					**EXPOSURES:**								**OUTCOMES:**					
					**OPIOID USE**				**HEADACHES FOR 3+ MONTHS**				**MDD**			**ASRD**		
**SNP**	**CHR**	**POS**	**EA**	**OA**	**eaf**	**effect**	**se**	**P-value**	**eaf**	**effect**	**se**	**P-value**	**effect**	**se**	**P-value**	**effect**	**se**	**P-value**
rs1050316	1	156434703	G	T	0.346	0.010	0.011	0.38	0.645	0.011	0.002	4.80E-06	-0.009	0.010	0.35	-0.028	0.018	0.13
rs10797381	1	3072482	A	T	0.229	0.011	0.013	0.39	0.237	0.015	0.003	7.00E-08	-0.014	0.012	0.25	0.004	0.021	0.86
rs10864035	1	207208335	T	C	0.408	0.015	0.011	0.16	0.407	0.011	0.002	1.30E-06	0.007	0.009	0.45	0.042	0.017	0.02
rs127204	1	112277693	C	A	0.400	0.059	0.011	8.50E-08	0.403	0.003	0.002	0.15	0.008	0.009	0.41	0.010	0.018	0.58
rs2036100	1	205907872	G	C	0.387	0.007	0.011	0.50	0.390	0.011	0.002	2.10E-06	-0.014	0.010	0.16	-0.013	0.018	0.46
rs945211	1	32191798	G	C	0.383	-0.051	0.011	3.20E-06	0.616	-0.002	0.002	0.30	-0.010	0.010	0.30	0.000	0.019	0.99
rs12618833	2	53864413	T	C	0.063	-0.021	0.022	0.34	0.067	0.022	0.005	2.40E-06	0.013	0.018	0.47	-0.013	0.034	0.71
rs139152977	2	199098906	A	T	0.025	0.164	0.034	1.90E-06	0.027	-0.013	0.007	0.07	0.010	0.030	0.74	0.044	0.056	0.43
rs16846013	2	212250195	A	G	0.211	0.019	0.013	0.16	0.214	0.014	0.003	9.60E-07	0.018	0.011	0.10	0.016	0.021	0.45
rs6431647	2	234809496	C	T	0.337	-0.041	0.011	3.10E-04	0.329	-0.011	0.002	3.20E-06	-0.015	0.010	0.13	0.006	0.018	0.73
rs13086611	3	49385417	T	A	0.303	-0.054	0.012	3.30E-06	0.305	0.001	0.003	0.65	-0.022	0.010	0.04	0.002	0.019	0.91
rs140729888	3	10521537	C	T	0.015	-0.205	0.045	4.30E-06	0.016	-0.005	0.009	0.58	-0.087	0.036	0.01	0.006	0.059	0.92
rs150448008	3	126884057	T	C	0.011	-0.044	0.051	0.39	0.012	-0.051	0.011	2.90E-06	0.003	0.036	0.92	0.056	0.063	0.38
rs247975	3	173107443	T	C	0.467	-0.050	0.011	3.80E-06	0.537	0.001	0.002	0.70	-0.006	0.009	0.53	-0.001	0.017	0.97
rs7631941	3	80480052	T	C	0.131	0.073	0.016	4.90E-06	0.131	-0.001	0.003	0.85	-0.016	0.014	0.24	0.008	0.027	0.77
rs13107325	4	103188709	T	C	0.074	0.100	0.020	1.10E-06	0.075	-0.009	0.004	0.05	-0.027	0.020	0.17	-0.013	0.041	0.75
rs76034781	4	3272782	A	G	0.077	0.098	0.020	1.20E-06	0.078	-0.009	0.004	0.04	0.038	0.021	0.07	0.096	0.031	0.002
rs769657	4	140877659	G	A	0.330	-0.053	0.011	4.80E-06	0.328	-0.005	0.002	0.05	-0.021	0.010	0.03	-0.030	0.019	0.11
rs10063803	5	176667327	C	A	0.168	-0.002	0.014	0.91	0.834	-0.015	0.003	1.70E-06	-0.006	0.012	0.65	-0.011	0.023	0.62
rs11746310	5	151391143	C	G	0.222	0.060	0.013	3.00E-06	0.223	0.005	0.003	0.07	0.008	0.011	0.48	0.011	0.021	0.61
rs7703548	5	28544070	C	A	0.052	-0.005	0.024	0.85	0.055	0.024	0.005	3.00E-06	-0.027	0.020	0.18	-0.043	0.037	0.25

© 2020 Rosoff DB et al. *JAMA Psychiatry*.

					EXPOSURES:								OUTCOMES:					
					OPIOID USE				HEADACHES FOR 3+ MONTHS				MDD			ASRD		
**SNP**	**CHR**	**POS**	**EA**	**OA**	**eaf**	**effect**	**se**	**P-value**	**eaf**	**effect**	**se**	**P-value**	**effect**	**se**	**P-value**	**effect**	**se**	**P-value**
rs1246939	6	4963177	A	C	0.340	0.017	0.012	0.14	0.342	0.012	0.002	0.00	0.006	0.010	0.55	0.027	0.019	0.15
rs2814982	6	34546560	T	C	0.102	0.082	0.018	3.20E-06	0.103	0.003	0.004	0.42	-0.008	0.015	0.57	-0.042	0.027	0.11
rs34350244	6	32561465	T	C	0.192	0.064	0.014	3.30E-06	0.196	0.001	0.003	0.77	-0.002	0.013	0.90	-0.019	0.024	0.43
rs9399923	6	96850379	G	A	0.218	0.032	0.013	0.01	0.773	0.014	0.003	3.70E-07	0.007	0.011	0.55	0.072	0.021	4.64E-04
rs11764780	7	2020904	C	G	0.185	-0.064	0.014	3.80E-06	0.183	-0.006	0.003	0.03	-0.030	0.012	0.01	-0.063	0.022	0.004
rs6975058	7	53040800	C	A	0.058	0.036	0.023	0.11	0.060	0.022	0.005	4.60E-06	0.017	0.020	0.38	0.028	0.036	0.45
rs116944254	8	31813439	T	C	0.053	0.005	0.024	0.83	0.057	-0.023	0.005	4.70E-06	0.041	0.022	0.07	0.047	0.049	0.34
rs117702299	8	106194797	A	G	0.017	-0.049	0.042	0.25	0.018	-0.042	0.009	2.10E-06	0.016	0.034	0.64	-0.023	0.060	0.70
rs117710046	8	59430010	T	C	0.016	0.210	0.043	1.20E-06	0.017	-0.007	0.009	0.44	0.008	0.031	0.80	-0.024	0.056	0.68
rs13263652	8	139346461	C	T	0.378	-0.051	0.011	4.80E-06	0.618	0.001	0.002	0.65	-0.001	0.009	0.88	0.008	0.018	0.65
rs12554874	9	37073902	T	C	0.376	-0.053	0.011	1.60E-06	0.376	0.002	0.002	0.37	-0.006	0.009	0.50	-0.020	0.018	0.27
rs13302047	9	81386827	G	C	0.192	0.069	0.014	3.60E-07	0.188	0.007	0.003	0.01	0.013	0.011	0.24	0.042	0.021	0.04
rs4837022	9	128475379	T	C	0.357	-0.054	0.011	1.40E-06	0.639	0.001	0.002	0.74	-0.001	0.010	0.96	-0.022	0.018	0.21
rs7022359	9	119251502	C	T	0.418	-0.013	0.011	0.22	0.411	-0.013	0.002	6.90E-08	0.001	0.009	0.95	0.010	0.018	0.55
rs10159545	10	21766969	G	C	0.349	0.054	0.011	2.20E-06	0.353	-0.001	0.002	0.59	-0.009	0.010	0.33	-0.007	0.018	0.70
rs761777	10	134938075	G	A	0.253	0.057	0.012	4.10E-06	0.256	0.002	0.003	0.56	0.029	0.012	0.02	0.055	0.022	0.01
rs1542724	11	46117183	C	A	0.269	0.057	0.012	2.70E-06	0.270	-0.003	0.003	0.25	0.013	0.010	0.19	0.012	0.019	0.53
rs4763505	12	10477712	T	A	0.317	-0.053	0.012	4.50E-06	0.682	0.003	0.002	0.28	-0.011	0.010	0.29	-0.037	0.020	0.06
rs7305700	12	57256380	T	A	0.386	-0.009	0.011	0.41	0.624	-0.011	0.002	3.50E-06	-0.015	0.009	0.10	0.003	0.018	0.85
rs7970708	12	72734488	G	A	0.056	-0.002	0.023	0.95	0.057	-0.023	0.005	4.50E-06	0.007	0.019	0.70	0.051	0.037	0.16
rs139243823	13	104770199	A	G	0.014	0.037	0.047	0.42	0.016	0.044	0.010	3.90E-06	0.076	0.052	0.14	0.082	0.089	0.35
rs191154499	13	37484160	T	C	0.014	-0.010	0.047	0.83	0.021	-0.042	0.009	1.40E-06	0.021	0.041	0.61	NA	NA	NA
rs56262049	13	42644705	C	T	0.182	-0.066	0.014	2.50E-06	0.185	-0.001	0.003	0.76	-0.011	0.012	0.35	-0.026	0.022	0.25

© 2020 Rosoff DB et al. *JAMA Psychiatry*.

					EXPOSURES:								OUTCOMES:					
					OPIOID USE				HEADACHES FOR 3+ MONTHS				MDD			ASRD		
**SNP**	**CHR**	**POS**	**EA**	**OA**	**eaf**	**effect**	**se**	**P-value**	**eaf**	**effect**	**se**	**P-value**	**effect**	**se**	**P-value**	**effect**	**se**	**P-value**
rs7319102	13	58252801	G	A	0.229	0.060	0.013	2.40E-06	0.773	0.001	0.003	0.82	0.012	0.011	0.28	0.051	0.020	0.01
rs11620998	14	99157430	C	T	0.375	0.000	0.011	0.98	0.621	0.011	0.002	3.30E-06	0.003	0.009	0.72	0.014	0.018	0.45
rs11624521	14	73760832	T	C	0.193	0.064	0.014	3.10E-06	0.198	0.006	0.003	0.03	-0.009	0.011	0.40	-0.020	0.022	0.37
rs113544339	15	86576076	G	T	0.023	-0.166	0.036	3.60E-06	0.024	-0.011	0.008	0.13	-0.047	0.031	0.13	-0.067	0.061	0.27
rs12594013	15	75596353	T	C	0.263	0.058	0.012	2.00E-06	0.264	-0.003	0.003	0.30	-0.009	0.011	0.41	-0.002	0.020	0.93
rs2289739	15	41801512	T	G	0.343	0.054	0.011	2.00E-06	0.348	0.003	0.002	0.23	0.009	0.010	0.36	-0.010	0.018	0.59
rs55916483	16	6070256	A	G	0.119	0.003	0.017	0.87	0.127	0.017	0.004	2.70E-06	0.013	0.015	0.39	NA	NA	NA
rs975376	16	73100503	C	G	0.164	0.071	0.015	1.00E-06	0.168	-0.004	0.003	0.22	-0.006	0.013	0.65	0.014	0.025	0.58
rs72848244	17	72195077	C	G	0.101	-0.009	0.018	0.60	0.100	-0.018	0.004	3.80E-06	0.010	0.016	0.54	0.034	0.030	0.25
rs9898793	17	43116313	T	C	0.261	0.056	0.012	4.20E-06	0.260	0.005	0.003	0.05	0.022	0.011	0.03	0.013	0.020	0.51
rs4987766	18	60904863	G	A	0.011	0.008	0.052	0.87	0.014	-0.048	0.010	2.70E-06	-0.016	0.047	0.73	NA	NA	NA
rs660948	18	55146073	C	G	0.307	-0.019	0.012	0.10	0.307	-0.014	0.003	1.90E-08	-0.017	0.010	0.09	-0.025	0.019	0.19
rs2297197	20	44673546	G	C	0.281	0.060	0.012	7.70E-07	0.287	0.000	0.003	0.99	0.028	0.010	0.006	0.028	0.020	0.15
rs6012735	20	36291470	G	A	0.426	-0.013	0.011	0.22	0.433	-0.011	0.002	2.60E-06	0.015	0.009	0.10	-0.004	0.017	0.82
rs6141277	20	30831157	C	G	0.392	0.052	0.011	2.20E-06	0.395	0.005	0.002	0.03	0.006	0.009	0.52	0.045	0.017	0.009
rs2845791	21	38035224	T	C	0.026	0.032	0.034	0.34	0.029	-0.034	0.007	1.80E-06	0.020	0.024	0.40	0.047	0.047	0.32
rs73228014	21	42752754	A	G	0.024	0.161	0.035	3.80E-06	0.025	0.007	0.007	0.34	0.010	0.052	0.85	NA	NA	NA

[a] For outcome phenotype MDD, summary statistics on MV instruments extracted from PGC GWAS excluding both UK Biobank (N = 29740) and 23andMe cohorts for total N = 143265 (no sample overlap).

**Abbreviations:**	
MRC-IEU	MRC Integrative Epidemiology Unit, University of Bristol UK
beta	effect estimate
se	standard error of effect estimate
SNP	single nucleotide polymorphism rsid variant identifier

© 2020 Rosoff DB et al. *JAMA Psychiatry*.

CHR	chromosome	
POS	base pair position	
eaf	effect allele frequency	
p-value	p value of effect estimate	
MDD	Major depression/ major depressive disorder: outcome phenotype	
ASRD	Anxiety and stress-related disorder: outcome phenotype	

© 2020 Rosoff DB et al. *JAMA Psychiatry*.

eTable 15. Bidirectional Single Variable Mendelian Randomization Results of Opioid Use Risk in UK Biobank Participants Versus Neuropsychiatric Disorders

													MR Presso	Steiger	Instrument strength [c,d]	
**EXPOSURE**	**OUTCOME**	**METHOD**	**N SNPS**	**beta**	**se**	**LCI**	**UCI**	**OR**	**OR LCI**	**OR UCI**	**P-value**	**Q P-value**	**P-value**	**P-value**	***F*-stat**	***F*-stat LCI**
**OPIOID USE**	**MDD[a]**	MR Egger	26	0.15	0.12	-0.08	0.38	1.17	0.92	1.47	0.21	0.24				
		Weighted median	26	0.16	0.05	0.07	0.26	1.18	1.07	1.30	9.69E-04					
		IVW	26	0.13	0.04	0.05	0.20	1.14	1.06	1.22	5.92E-04	0.29	0.28	2.94E-78	31.85	30.04
		Weighted mode	26	0.17	0.09	-0.01	0.34	1.18	0.99	1.41	0.07					
		MR Egger intercept		0.00	0.01	-0.02	0.01	1.00	0.98	1.01	0.82					
**OPIOID USE**	**ASRD**	MR Egger	24	-0.03	0.25	-0.52	0.47	0.97	0.59	1.60	0.91	0.04				
		Weighted median	24	0.19	0.10	-0.01	0.39	1.21	0.99	1.48	0.06					
		IVW	24	0.18	0.08	0.01	0.34	1.19	1.01	1.41	0.04	0.04	0.04	1.23E-18	0.010	32.18
		Weighted mode	24	0.10	0.17	-0.24	0.43	1.10	0.79	1.54	0.58					
		MR Egger intercept		0.01	0.02	-0.02	0.05	1.01	0.98	1.05	0.40					
		OUTLIER CORRECTED														
		MR Egger	23	0.23	0.24	-0.25	0.70	1.26	0.78	2.02	0.36	0.19				
		Weighted median	23	0.20	0.10	0.00	0.40	1.22	1.00	1.49	0.05					
		IVW	23	0.22	0.07	0.07	0.36	1.24	1.07	1.44	0.004	0.24	0.16	2.15E-19	0.010	32.57
		Weighted mode	23	0.03	0.20	-0.36	0.42	1.03	0.70	1.53	0.87					
		MR Egger intercept		0.00	0.02	-0.03	0.03	1.00	0.97	1.03	0.96					
**MDD[b]**	**OPIOID USE**	MR Egger	72	0.43	0.22	0.01	0.85	1.54	1.01	2.34	0.05	>0.99				
		Weighted median	72	0.22	0.07	0.09	0.35	1.25	1.10	1.42	0.001					
		IVW	72	0.17	0.05	0.07	0.26	1.18	1.08	1.30	4.45E-04	>0.99	>0.99	1.03E-14	0.005	24.32
		Weighted mode	72	0.28	0.15	-0.02	0.58	1.32	0.98	1.78	0.07					

© 2020 Rosoff DB et al. *JAMA Psychiatry*.

													MR Presso	Steiger	Instrument strength [c,d]	
**EXPOSURE**	**OUTCOME**	**METHOD**	**N SNPS**	**beta**	**se**	**LCI**	**UCI**	**OR**	**OR LCI**	**OR UCI**	**P-value**	**Q P-value**	**P-value**	**P-value**	***F*-stat**	***F*-stat LCI**
		MR Egger Intercept		-0.01	0.01	-0.02	0.00	0.99	0.98	1.00	0.22					
**ASRD**	**OPIOID USE**	MR Egger	17	0.01	0.13	-0.24	0.26	1.01	0.78	1.30	0.94	0.07				
		Weighted median	17	-0.02	0.04	-0.10	0.07	0.98	0.90	1.07	0.70					
		IVW	17	0.04	0.04	-0.03	0.11	1.04	0.97	1.12	0.29	0.09	0.21	<0.001	25.02	21.17
		Weighted mode	17	-0.05	0.08	-0.20	0.11	0.96	0.82	1.12	0.58					
		MR Egger intercept		0.00	0.01	-0.02	0.03	1.00	0.98	1.03	0.81					

[a] For outcome phenotype MDD, summary statistics on NSAIDs instruments extracted from PGC GWAS excluding both UK Biobank (N = 29740) 23andMe cohorts for total N = 143265
[b] For exposure phenotype MDD, summary statistics on MDD instruments extracted from PGC GWAS including both UK Biobank (N = 29740) and 23andMe cohorts for total N = 480359 (% UKB = 5.6)
[c] R^2 for each instrument variant: R^2 = [beta.exposure^2]/[se.exposure^2*N + beta.exposure^2] ; R^2 combined= SUM[R^2], where beta.exposure = SNP exposure effect, se. exposure = standard error of SNP exposure effect
[d] Combined *F*-statistic (also lower bound thereof) calculated according to Burgess et al. (2016): [R^2combined/(1-R^2combined)]*[(N-K-1)/K], where N = GWAS sample size, K = number variants comprising instrument.

**Abbreviations:**	
SVMR	Single-variable Mendelian randomization
IVW	Inverse variance weighted MR
UKB	UK Biobank
N SNPs	number of single nucleotide polymorphisms in instrument
beta	MR effect estimate
se	standard error of MR effect estimate
LCI	lower bound of 95% confidence interval of estimate
UCI	upper bound of 95% confidence interval of estimate
OR	odds ratio for binary outcome
OR LCI	lower bound of 95% confidence interval of odds ratio estimate
OR UCI	upper bound of 95% confidence interval of odds ratio estimate
P-value	P-value of MR effect estimate
**Heterogeneity Tests:**	
Q P-value	Cochran Q statistic P-value
**MR PRESSO test:**	

© 2020 Rosoff DB et al. *JAMA Psychiatry*.

RSSobs	residual sums of squares of observations
P-value	P-value of global test
**Directionality test**	
SNPs R^2 exposure	variance in exposure explained by exposure instruments
SNPs R^2 outcome<br>Steiger p-value	variance in outcome explained by exposure instruments<br>strength of evidence against null hypotheses in favor of alternative direction of causality TRUE
**Instrument strength**	
F-stat	F-statistic
F-stat LCI	lower bound 95% confidence interval of F-stat

© 2020 Rosoff DB et al. *JAMA Psychiatry*.

eTable 16. Bidirectional Single Variable Mendelian Randomization Results of Salicylic Acid and Derivatives Use Risk in UK Biobank Participants Versus Neuropsychiatric Disorders

													MR Presso	Steiger	Instrument strength [c,d]	
**EXPOSURE**	**OUTCOME**	**METHOD**	**N SNPs**	**beta**	**se**	**LCI**	**UCI**	**OR**	**OR LCI**	**OR UCI**	**P-value**	**Q P-value**	**P-value**	**P-value**	***F*-stat**	***F*-stat LCI**
**SALICYLIC ACID USE**	**MDD[a]**	MR Egger	27	-0.19	0.12	-0.42	0.04	0.83	0.66	1.04	0.12	0.34				
		Weighted median	27	0.04	0.06	-0.08	0.16	1.04	0.92	1.18	0.52					
		IVW	27	0.07	0.05	-0.03	0.17	1.07	0.97	1.18	0.15	0.15	0.09	<0.001	43.55	39.46
		Weighted mode	27	0.02	0.11	-0.18	0.23	1.02	0.83	1.26	0.84					
		MR Egger intercept		0.02	0.01	0.00	0.03	1.02	1.00	1.03	0.02					
**SALICYLIC ACID USE**	**ASRD**	MR Egger	21	-0.08	0.26	-0.59	0.42	0.92	0.56	1.52	0.75	0.87				
		Weighted median	21	0.13	0.12	-0.09	0.36	1.14	0.91	1.44	0.25					
		IVW	21	0.17	0.09	0.00	0.34	1.18	1.00	1.40	0.05	0.86	0.87	1.50E-25	0.009	44.02
		Weighted mode	21	0.12	0.17	-0.22	0.46	1.13	0.81	1.58	0.49					
		MR Egger intercept		0.02	0.02	-0.01	0.05	1.02	0.99	1.05	0.31					
**MDD[b]**	**SALICYLIC ACID USE**	MR Egger	93	0.20	0.15	-0.09	0.49	1.22	0.91	1.63	0.18	0.46				
		Weighted median	93	0.10	0.05	0.01	0.19	1.11	1.01	1.21	0.02					
		IVW	93	0.10	0.03	0.03	0.16	1.10	1.03	1.17	0.00	0.48	0.50	7.30E-44	0.006	30.97
		Weighted mode	93	0.14	0.11	-0.08	0.36	1.15	0.92	1.43	0.22					
		MR Egger Intercept		0.00	0.00	-0.01	0.01	1.00	0.99	1.01	0.47					
**ASRD**	**SALICYLIC ACID USE**	MR Egger	10	-0.02	0.09	-0.19	0.15	0.98	0.82	1.16	0.79	0.91				
		Weighted median	10	0.06	0.04	-0.02	0.13	1.06	0.98	1.14	0.13					
		IVW	10	0.05	0.03	-0.01	0.10	1.05	0.99	1.11	0.11	0.90	0.91	<0.001	22.91	18.16

© 2020 Rosoff DB et al. *JAMA Psychiatry*.

													MR Presso	Steiger	Instrument strength [c,d]	
**EXPOSURE**	**OUTCOME**	**METHOD**	**N SNPs**	**beta**	**se**	**LCI**	**UCI**	**OR**	**OR LCI**	**OR UCI**	**P-value**	**Q P-value**	**P-value**	**P-value**	***F*-stat**	***F*-stat LCI**
		Weighted mode	10	0.08	0.06	-0.05	0.20	1.08	0.96	1.22	0.25					
		MR Egger Intercept		-0.19	0.12	-0.42	0.04	0.83	0.66	1.04	0.12					

[a] For outcome phenotype MDD, summary statistics on NSAIDs instruments extracted from PGC GWAS excluding both UK Biobank (N = 29740) 23andMe cohorts for total N = 143265
[b] For exposure phenotype MDD, summary statistics on MDD instruments extracted from PGC GWAS including both UK Biobank (N = 29740) and 23andMe cohorts for total N = 480359 (% UKB = 5.6)
[c] R^2 for each instrument variant: R^2 = [beta.exposure^2]/[se.exposure^2*N + beta.exposure^2] ; R^2 combined= SUM[R^2], where beta.exposure = SNP exposure effect, se. exposure = standard error of SNP exposure effect
[d] Combined *F*-statistic (also lower bound thereof) calculated according to Burgess et al. (2016): [R^2combined/(1-R^2combined)]*[(N-K-1)/K], where N = GWAS sample size, K = number variants comprising instrument.

**Abbreviations:**	
SVMR	Single-variable Mendelian randomization
IVW	Inverse variance weighted MR
UKB	UK Biobank
N SNPs	number of single nucleotide polymorphisms in instrument
beta	MR effect estimate
se	standard error of MR effect estimate
LCI	lower bound of 95% confidence interval of estimate
UCI	upper bound of 95% confidence interval of estimate
OR	odds ratio for binary outcome
OR LCI	lower bound of 95% confidence interval of odds ratio estimate
OR UCI	upper bound of 95% confidence interval of odds ratio estimate
P-value	P-value of MR effect estimate
**Heterogeneity Tests:**	
Q	Q statistic
Q df	Q statistic degrees of freedom
Q P-value	Q statistic P-value
**MR PRESSO test:**	
RSSobs	residual sums of squares of observations
P-value	P-value of global test
**Directionality test**	
SNPs R^2 exposure	variance in exposure explained by exposure instruments
SNPs R^2 outcome	variance in outcome explained by exposure instruments

© 2020 Rosoff DB et al. *JAMA Psychiatry*.

Steiger p-value	strength of evidence against null hypotheses in favor of alternative direction of causality TRUE
**Instrument strength**	
F-stat	F-statistic
F-stat LCI	lower bound 95% confidence interval of F-stat

© 2020 Rosoff DB et al. *JAMA Psychiatry*.

eTable 17. Bidirectional Single Variable Mendelian Randomization Results of Anilide Use Risk in UK Biobank Participants Versus Neuropsychiatric Disorders

													MR Presso	Steiger	Instrument strength [c,d]	
**EXPOSURE**	**OUTCOME**	**METHOD**	**N SNPs**	**beta**	**se**	**LCI**	**UCI**	**OR**	**OR LCI**	**OR UCI**	**P-value**	**Q P-value**	**P-value**	**P-value**	***F*-stat**	***F*-stat LCI**
**ANILIDE USE**	**MDD[a]**	MR Egger	29	0.05	0.23	-0.40	0.50	1.05	0.67	1.64	0.82	0.24				
		Weighted median	29	0.13	0.08	-0.03	0.29	1.14	0.97	1.34	0.10					
		IVW	29	0.13	0.06	0.01	0.24	1.14	1.01	1.27	0.03	0.28	0.28	1.29E-44	19.04	16.48
		Weighted mode	29	0.14	0.17	-0.18	0.46	1.15	0.83	1.59	0.42					
		MR Egger intercept		0.00	0.01	-0.01	0.02	1.00	0.99	1.02	0.73					
**ANILIDE USE**	**ASRD**	MR Egger	28	-0.16	0.42	-0.99	0.66	0.85	0.37	1.94	0.70	0.53				
		Weighted median	28	-0.05	0.16	-0.35	0.26	0.96	0.70	1.30	0.77					
		IVW	28	0.01	0.11	-0.20	0.22	1.01	0.82	1.25	0.93	0.58	0.58	5.63E-08	20.58	19.23
		Weighted mode	28	-0.18	0.35	-0.86	0.50	0.83	0.42	1.65	0.61					
		MR Egger intercept		0.01	0.02	-0.02	0.04	1.01	0.98	1.04	0.67					
**MDD[b]**	**ANILIDE USE**	MR Egger	89	0.13	0.14	-0.15	0.41	1.14	0.86	1.50	0.36	0.06				
		Weighted median	89	0.20	0.04	0.13	0.28	1.22	1.13	1.32	2.21E-07					
		IVW	89	0.19	0.03	0.13	0.24	1.20	1.14	1.28	4.30E-10	0.07	0.08	2.93E-59	29.95	30.24
		Weighted mode	89	0.25	0.10	0.06	0.44	1.28	1.06	1.55	0.01					
		MR Egger intercept		0.00	0.00	-0.01	0.01	1.00	0.99	1.01	0.69					
**ASRD**	**ANILIDE USE**	MR Egger	17	-0.01	0.08	-0.16	0.14	0.99	0.85	1.15	0.86	0.07				
		Weighted median	17	0.00	0.03	-0.05	0.06	1.00	0.95	1.06	0.92					
		IVW	17	0.02	0.02	-0.03	0.06	1.02	0.97	1.06	0.43	0.09	0.09	<.001	25.02	21.17

© 2020 Rosoff DB et al. *JAMA Psychiatry*.

													**MR Presso**	**Steiger**	**Instrument strength [c,d]**	
**EXPOSURE**	**OUTCOME**	**METHOD**	**N SNPs**	**beta**	**se**	**LCI**	**UCI**	**OR**	**OR LCI**	**OR UCI**	**P-value**	**Q P-value**	**P-value**	**P-value**	***F*-stat**	***F*-stat LCI**
		Weighted mode	17	0.00	0.05	-0.11	0.10	1.00	0.90	1.11	0.95					
		MR Egger intercept		0.00	0.01	-0.01	0.02	1.00	0.99	1.02	0.68					

[a] For outcome phenotype MDD, summary statistics on NSAIDs instruments extracted from PGC GWAS excluding both UK Biobank (N = 29740) 23andMe cohorts for total N = 143265
[b] For exposure phenotype MDD, summary statistics on MDD instruments extracted from PGC GWAS including both UK Biobank (N = 29740) and 23andMe cohorts for total N = 480359 (% UKB = 5.6)
[c] R^2 for each instrument variant: R^2 = [beta.exposure^2]/[se.exposure^2*N + beta.exposure^2] ; R^2 combined= SUM[R^2], where beta.exposure = SNP exposure effect, se. exposure = standard error of SNP exposure effect
[d] Combined *F*-statistic (also lower bound thereof) calculated according to Burgess et al. (2016): [R^2combined/(1-R^2combined)]*[(N-K-1)/K], where N = GWAS sample size, K = number variants comprising instrument.

**Abbreviations:**	
SVMR	Single-variable Mendelian randomization
IVW	Inverse variance weighted MR
UKB	UK Biobank
N SNPs	number of single nucleotide polymorphisms in instrument
beta	MR effect estimate
se	standard error of MR effect estimate
LCI	lower bound of 95% confidence interval of estimate
UCI	upper bound of 95% confidence interval of estimate
OR	odds ratio for binary outcome
OR LCI	lower bound of 95% confidence interval of odds ratio estimate
OR UCI	upper bound of 95% confidence interval of odds ratio estimate
P-value	P-value of MR effect estimate
**Heterogeneity Tests:**	
Q	Q statistic
Q df	Q statistic degrees of freedom
Q P-value	Q statistic P-value
**MR PRESSO test:**	
RSSobs	residual sums of squares of observations
P-value	P-value of global test
**Directionality test**	
SNPs R^2 exposure	variance in exposure explained by exposure instruments
SNPs R^2 outcome	variance in outcome explained by exposure instruments

© 2020 Rosoff DB et al. *JAMA Psychiatry*.

**Instrument strength**	
F-stat	F-statistic
F-stat LCI	lower bound 95% confidence interval of F-stat

© 2020 Rosoff DB et al. *JAMA Psychiatry*.

eTable 18. Bidirectional Single Variable Mendelian Randomization Results of NSAID Use Risk in UK Biobank Participants Versus Neuropsychiatric Disorders

													MR-PRESSO	Steiger	Instrument strength [c,d]	
**EXPOSURE**	**OUTCOME**	**METHOD**	**N SNPs**	**beta**	**se**	**LCI**	**UCI**	**OR**	**OR LCI**	**OR UCI**	**P-value**	**Q P-value**	**P-value**	**P-value**	***F*-stat**	***F*-stat LCI**
**NSAID USE**	**MDD[a]**	MR Egger	29	0.10	0.18	-0.24	0.45	1.11	0.78	1.58	0.56	0.56				
		Weighted median	29	0.18	0.07	0.05	0.31	1.20	1.05	1.37	0.008					
		IVW	29	0.11	0.05	0.02	0.21	1.12	1.02	1.23	0.02	0.61	0.30	3.76E-57	32.09	28.72
		Weighted mode	29	0.29	0.14	0.02	0.56	1.34	1.02	1.76	0.04					
		MR Egger intercept	29	0.00	0.01	-0.02	0.02	1.00	0.99	1.02	0.97					
**NSAID USE**	**ASRD**	MR Egger	29	0.17	0.42	-0.66	0.99	1.18	0.52	2.68	0.70	0.66				
		Weighted median	29	0.12	0.14	-0.15	0.39	1.13	0.86	1.47	0.38					
		IVW	29	0.25	0.10	0.06	0.44	1.29	1.06	1.56	0.01	0.71	0.81	3.40E-09	25.52	22.52
		Weighted mode	29	0.02	0.26	-0.50	0.54	1.02	0.61	1.71	0.95					
		MR Egger intercept	29	0.00	0.02	-0.03	0.04	1.00	0.97	1.04	0.83					
**MDD[b]**	**NSAID USE**	MR Egger	93	0.07	0.15	-0.22	0.36	1.07	0.81	1.43	0.63	0.04				
		Weighted median	93	0.16	0.04	0.08	0.24	1.17	1.08	1.27	1.30E-04					
		IVW	93	0.13	0.03	0.07	0.19	1.14	1.08	1.21	1.68E-05	0.04	0.5	1.11E-44	32.10	30.24
		Weighted mode	93	0.22	0.11	0.01	0.43	1.24	1.01	1.54	0.05					
		MR Egger intercept		0.00	0.00	-0.01	0.01	1.00	0.99	1.01	0.66					
		OUTLIER CORRECTED														
		MR Egger	92	0.08	0.14	-0.19	0.35	1.08	0.82	1.43	0.57	0.13				
		Weighted median	92	0.16	0.04	0.08	0.24	1.17	1.08	1.27	1.33E-04					
		IVW	92	0.14	0.03	0.08	0.20	1.15	1.09	1.22	1.77E-06	0.14	0.16	2.10E-45	32.29	30.37

© 2020 Rosoff DB et al. *JAMA Psychiatry*.

													MR-PRESSO	Steiger	Instrument strength [c,d]	
**EXPOSURE**	**OUTCOME**	**METHOD**	**N SNPs**	**beta**	**se**	**LCI**	**UCI**	**OR**	**OR LCI**	**OR UCI**	**P-value**	**Q P-value**	**P-value**	**P-value**	***F*-stat**	***F*-stat LCI**
		Weighted mode	92	0.22	0.11	0.01	0.43	1.25	1.01	1.54	0.04					
		MR Egger intercept		0.00	0.00	-0.01	0.01	1.00	0.99	1.01	0.65					
**ASRD**	**NSAID USE**	MR Egger	17	0.05	0.09	0.59	0.21	1.05	1.81	1.24	0.59	0.03	0.19	5.64E-57	24.45	21.83
		Weighted median	17	0.02	0.03	0.57	0.07	1.02	1.77	1.08	0.57					
		IVW	17	0.01	0.03	0.80	0.06	1.01	2.23	1.06	0.80	0.04				
		Weighted mode	17	0.03	0.05	0.53	0.14	1.03	1.71	1.15	0.53					
		MR Egger intercept		0.00	0.01	0.53	0.01	1.00	1.71	1.01	0.63					

[a] For outcome phenotype MDD, summary statistics on NSAIDs instruments extracted from PGC GWAS excluding both UK Biobank (N = 29740) 23andMe cohorts for total N = 143265

[b] For exposure phenotype MDD, summary statistics on MDD instruments extracted from PGC GWAS including both UK Biobank (N = 29740) and 23andMe cohorts for total N = 480359 (% UKB = 5.6)

[c] R^2 for each instrument variant: R^2 = [beta.exposure^2]/[se.exposure^2*N + beta.exposure^2] ; R^2 combined= SUM[R^2], where beta.exposure = SNP exposure effect, se. exposure = standard error of SNP exposure effect

[d] Combined *F*-statistic (also lower bound thereof) calculated according to Burgess et al. (2016): [R^2combined/(1-R^2combined)]*[(N-K-1)/K], where N = GWAS sample size, K = number variants comprising instrument.

**Abbreviations:**	
SVMR	Single-variable Mendelian randomization
IVW	Inverse variance weighted MR
UKB	UK Biobank
N SNPs	number of single nucleotide polymorphisms in instrument
beta	MR effect estimate
se	standard error of MR effect estimate
LCI	lower bound of 95% confidence interval of estimate
UCI	upper bound of 95% confidence interval of estimate
OR	odds ratio for binary outcome
OR LCI	lower bound of 95% confidence interval of odds ratio estimate
OR UCI	upper bound of 95% confidence interval of odds ratio estimate
P-value	P-value of MR effect estimate
**Heterogeneity Tests:**	

© 2020 Rosoff DB et al. *JAMA Psychiatry*.

Q	Q statistic
Q df	Q statistic degrees of freedom
Q P-value	Q statistic P-value
**MR PRESSO test:**	
RSSobs	residual sums of squares of observations
P-value	P-value of global test
**Directionality test**	
SNPs R^2 exposure	variance in exposure explained by exposure instruments
SNPs R^2 outcome	variance in outcome explained by exposure instruments
**Instrument strength**	
F-stat	F-statistic
F-stat LCI	lower bound 95% confidence interval of F-stat

© 2020 Rosoff DB et al. *JAMA Psychiatry*.

eTable 19. Multivariable Mendelian Randomization Results of Opioid and Non-Opioid Use Risk in UK Biobank Participants on Neuropsychiatric Disorders[a]

												Heterogeneity test			MR-PRESSO global test		
**OUTCOME**	**EXPOSURES**	**METHOD**	**N SNPs**	**beta**	**se**	**LCI**	**UCI**	**OR**	**OR LCI**	**OR UCI**	**P-value**	**Q**	**Q df**	**Q P-value**	**RSS obs**	**P-value**	**N outliers**
MDD[a]	**OPIOID USE**	MV IVW	92	0.13	0.05	0.04	0.23	1.14	1.04	1.25	0.005	85.14	88.00	0.57	94.58	0.63	0
	NSAID USE			-0.02	0.08	-0.18	0.14	0.98	0.84	1.15	0.81						
	ANILIDE USE			0.07	0.09	-0.11	0.25	1.07	0.89	1.29	0.46						
	SALICYLIC ACID USE			-0.01	0.06	-0.12	0.10	0.99	0.89	1.11	0.88						
MDD[a]	**OPIOID USE**	MV Egger	92	0.12	0.05	0.02	0.23	1.13	1.02	1.26	0.02	84.98	87.00	0.54			0
	NSAID USE			0.06	0.10	-0.14	0.25	1.06	0.87	1.28	0.56						
	ANILIDE USE			-0.03	0.09	-0.20	0.14	0.97	0.82	1.15	0.72						
	SALICYLIC ACID USE			-0.02	0.06	-0.14	0.10	0.98	0.87	1.11	0.76						
	Intercept			0.00	0.00	-0.01	0.01	1.00	0.99	1.01	0.69						
ASRD	**OPIOID USE**	MV IVW	86	0.26	0.10	0.07	0.45	1.30	1.08	1.56	0.006	91.49	82.00	0.22	86.34	0.59	0
	NSAID USE			0.32	0.17	-0.01	0.64	1.37	0.99	1.90	0.06						
	ANILIDE USE			-0.34	0.19	-0.71	0.03	0.71	0.49	1.03	0.07						
	SALICYLIC ACID USE			-0.05	0.12	-0.28	0.19	0.96	0.76	1.21	0.70						
ASRD	**OPIOID USE**	MV Egger	86	0.17	0.11	-0.05	0.38	1.18	0.95	1.46	0.13	87.98	81.00	0.28			0
	NSAID USE			-0.45	0.20	-0.84	-0.07	0.64	0.43	0.94	0.02						
	ANILIDE USE			0.20	0.17	-0.14	0.55	1.23	0.87	1.73	0.24						
	SALICYLIC ACID USE			-0.17	0.14	-0.44	0.10	0.84	0.64	1.10	0.21						
	Intercept			0.01	0.01	0.00	0.03	1.01	1.00	1.03	0.07						

[a] For outcome phenotype MDD, summary statistics on MV instruments extracted from PGC GWAS excluding both UK Biobank (N = 29740) and 23andMe cohorts for total N = 143265 (no sample overlap)

© 2020 Rosoff DB et al. *JAMA Psychiatry*.

**Abbreviations:**	
MVMR	Multivariable Mendelian randomization
UKB	UK Biobank
N SNPs	Number of single nucleotide polymorphisms in instrument
beta	effect estimate
se	standard error of effect estimate
LCI	lower bound of 95% confidence interval of estimate
UCI	upper bound of 95% confidence interval of estimate
OR	odds ratio for binary outcome
OR LCI	lower bound of 95% confidence interval of odds ratio estimate
OR UCI	upper bound of 95% confidence interval of odds ratio estimate
P-value	P-value of effect estimate
**Heterogeneity Tests:**	
Q	Q statistic
Q df	Q statistic degrees of freedom
Q P-value	Q statistic P-value
**MR PRESSO test:**	
RSS obs	residual sums squares of observations
P-value	P-value of global test

© 2020 Rosoff DB et al. *JAMA Psychiatry*.

eTable 20. Multivariable Mendelian Randomization Results of Opioid Use Risk Accounting for Chronic Pain in UK Biobank Participants on MDD and ASRD

												Heterogeneity test			MR-PRESSO global test		
OUTCOME	EXPOSURES	METHOD	N SNPs	beta	se	LCI	UCI	OR	OR LCI	OR UCI	P-value	Q	Q df	Q P-value	RSS obs	P-value	N Outliers
	-																
**MDD[a]**	**OPIOID**	MV IVW	47	0.12	0.04	0.05	0.19	1.12	1.05	1.21	.001	54.43	45	0.16	59.05	0.18	None
	BACK PAIN			0.12	0.32	-0.50	0.74	1.13	0.60	2.09	.71						
	**OPIOID**	MV Egger	47	0.11	0.09	-0.06	0.28	1.12	0.95	1.30	.19	54.40	44	0.14			
	BACK PAIN			0.11	0.33	-0.53	0.75	1.12	0.59	2.11	.74						
	Intercept			0.00	0.01	-0.01	0.01	1.00	0.99	1.01	.87						
**MDD**	**OPIOID**	MV IVW	61	0.14	0.03	0.08	0.21	1.15	1.08	1.23	<0.001	69.84	59	0.16	74.48	0.19	None
	HEADACHES			0.05	0.17	-0.28	0.39	1.06	0.76	1.47	.75						
	**OPIOID**	MV Egger	61	0.12	0.07	-0.02	0.26	1.13	0.98	1.29	.08	69.68	58	0.14			
	HEADACHES			0.01	0.22	-0.42	0.43	1.01	0.66	1.54	.98						
	Intercept			0.00	0.00	-0.01	0.01	1.00	0.99	1.01	.72						
**MDD**	**OPIOID**	MV IVW	46	0.14	0.03	0.07	0.20	1.15	1.07	1.22	2.74E-04	50.45	44	0.23	54.75	0.28	None
	NECK/ SHOULDER PAIN			-0.50	0.30	-1.10	0.10	0.61	0.33	1.10	0.10						
	**OPIOID**	MV Egger	46	0.11	0.08	-0.04	0.26	1.12	0.96	1.30	0.15	50.31	43	0.21			
	NECK/ SHOULDER PAIN			-0.50	0.31	-1.10	0.10	0.60	0.33	1.10	0.10						
	Intercept			0.00	0.01	-0.01	0.01	1.00	0.99	1.01	0.73						
**MDD**	**OPIOID**	MV IVW	41	0.14	0.04	0.07	0.22	1.15	1.07	1.24	3.42E-04	49.93	39	0.11	55.64	0.11	None
	KNEE PAIN			-0.61	0.45	-1.50	0.28	0.54	0.22	1.32	0.18						
	**OPIOID**	MV Egger	41	0.15	0.10	-0.05	0.34	1.16	0.96	1.40	0.14	49.93	38	0.09			

© 2020 Rosoff DB et al. *JAMA Psychiatry*.

												Heterogeneity test			MR-PRESSO global test		
**OUTCOME**	**EXPOSURES**	**METHOD**	**N SNPs**	**beta**	**se**	**LCI**	**UCI**	**OR**	**OR LCI**	**OR UCI**	**P-value**	**Q**	**Q df**	**Q P-value**	**RSS obs**	**P-value**	**N Outliers**
	KNEE PAIN			-0.61	0.46	-1.52	0.29	0.54	0.22	1.33	0.18						
	Intercept			0.00	0.01	-0.01	0.01	1.00	0.99	1.01	0.99						
**MDD**	**OPIOID**	MV IVW	44	0.13	0.03	0.07	0.19	1.14	1.07	1.21	1.97E-04	47.14	42	0.27	52.25	0.31	None
	HIP PAIN			0.23	0.26	-0.27	0.74	1.26	0.76	2.09	0.37						
	**OPIOID**	MV Egger	44	0.18	0.08	0.03	0.33	1.19	1.03	1.39	0.02	46.62	41	0.25			
	HIP PAIN			0.30	0.28	-0.25	0.85	1.35	0.78	2.33	0.28						
	Intercept			0.00	0.01	-0.01	0.01	1.00	0.99	1.01	0.50						
**MDD**	**OPIOID**	MV IVW	41	0.14	0.04	0.07	0.20	1.15	1.07	1.23	4.42E-04	46.52	39	0.19	50.94	0.22	None
	ABDOMINAL PAIN			-0.24	0.19	-0.61	0.14	0.79	0.54	1.15	0.21						
	**OPIOID**	MV Egger	41	0.10	0.10	-0.10	0.29	1.10	0.90	1.34	0.35	46.30	38	0.17			
	ABDOMINAL PAIN			-0.21	0.20	-0.61	0.19	0.81	0.54	1.21	0.30						
	Intercept			0.00	0.01	-0.01	0.01	1.00	0.99	1.01	0.67						
**ASRD**	**OPIOID**	MV IVW	42	0.19	0.08	0.04	0.34	1.21	1.04	1.41	0.002	65.72	40	0.01	71.78	0.01	one
	BACK PAIN			-0.04	0.77	-1.55	1.47	0.96	0.21	4.36	0.96						
	**OPIOID**	MV Egger	42	0.10	0.18	-0.26	0.46	1.11	0.77	1.58	0.58	65.21	39	0.01			
	BACK PAIN			-0.09	0.78	-1.62	1.44	0.91	0.20	4.24	0.91						
	Intercept			0.01	0.01	-0.02	0.03	1.01	0.98	1.03	0.57						
	OUTLIER CORRECTED																
	**OPIOID**	MV IVW	42	0.23	0.07	0.09	0.37	1.26	1.09	1.45	0.002	55.15	39	0.06	60.58	0.07	
	BACK PAIN			-0.23	0.72	-1.63	1.18	0.80	0.20	3.26	0.75						
	**OPIOID**	MV Egger	42	0.24	0.18	-0.11	0.59	1.27	0.90	1.80	0.18	55.14	38	0.04			
	BACK PAIN			-0.22	0.73	-1.65	1.21	0.80	0.19	3.36	0.76						

© 2020 Rosoff DB et al. *JAMA Psychiatry*.

												Heterogeneity test			MR-PRESSO global test		
**OUTCOME**	**EXPOSURES**	**METHOD**	**N SNPs**	**beta**	**se**	**LCI**	**UCI**	**OR**	**OR LCI**	**OR UCI**	**P-value**	**Q**	**Q df**	**Q P-value**	**RSS obs**	**P-value**	**N Outliers**
	Intercept			0.00	0.01	-0.02	0.02	1.00	0.98	1.02	0.95						
**ASRD**	**OPIOID**	MV IVW	57	0.26	0.07	0.12	0.39	1.29	1.13	1.48	4.60E-04	76.94	55	0.03	82.78	0.03	None
	HEADACHES			0.04	0.36	-0.67	0.75	1.04	0.51	2.12	0.91						
	**OPIOID**	MV Egger	57	0.11	0.14	-0.17	0.39	1.12	0.84	1.48	0.45	74.95	54	0.03			
	HEADACHES			-0.31	0.47	-1.23	0.60	0.73	0.29	1.83	0.50						
	Intercept			0.01	0.01	-0.01	0.03	1.01	0.99	1.03	0.23						
**ASRD**	**OPIOID**	MV IVW	42	0.25	0.08	0.10	0.39	1.28	1.11	1.48	1.96E-03	60.63	40	0.02	67.12	0.02	None
	NECK/ SHOULDER PAIN			0.67	0.73	-0.76	2.09	1.95	0.47	8.11	0.36						
	**OPIOID**	MV Egger	42	0.02	0.17	-0.31	0.36	1.02	0.73	1.43	0.90	57.47	39	0.03			
	NECK/ SHOULDER PAIN			0.65	0.72	-0.75	2.06	1.92	0.47	7.84	0.36						
	Intercept			0.02	0.01	0.00	0.03	1.02	1.00	1.04	0.14						
**ASRD**	**OPIOID**	MV IVW	40	0.22	0.08	0.07	0.37	1.25	1.07	1.45	5.00E-03	62.89	38	0.01	68.91	0.01	None
	KNEE PAIN			-0.25	1.02	-2.26	1.76	0.78	0.10	5.81	0.81						
	**OPIOID**	MV Egger	40	-0.06	0.20	-0.44	0.33	0.95	0.64	1.39	0.78	59.13	37	0.01			
	KNEE PAIN			-0.23	1.01	-2.20	1.74	0.79	0.11	5.72	0.82						
	Intercept			0.02	0.01	-0.01	0.04	1.02	0.99	1.04	0.13						
**ASRD**	**OPIOID**	MV IVW	34	0.23	0.08	0.07	0.39	1.26	1.08	1.48	4.00E-03	55.02	32	0.01	62.42	0.01	None
	HIP PAIN			-1.45	1.64	-4.67	1.77	0.23	0.01	5.86	0.38						
	**OPIOID**	MV Egger	34	-0.14	0.24	-0.60	0.32	0.87	0.55	1.38	0.55	50.46	31	0.02			
	HIP PAIN			-1.80	1.61	-4.96	1.36	0.17	0.01	3.90	0.26						

© 2020 Rosoff DB et al. *JAMA Psychiatry*.

												Heterogeneity test			MR-PRESSO global test		
OUTCOME	EXPOSURES	METHOD	N SNPs	beta	se	LCI	UCI	OR	OR LCI	OR UCI	P-value	Q	Q df	Q P-value	RSS obs	P-value	N Outliers
	Intercept			0.03	0.02	0.00	0.06	1.03	1.00	1.06	0.09						
**ASRD**	**OPIOID**	MV IVW	38	0.24	0.07	0.10	0.38	1.27	1.11	1.46	1.00E-03	49.11	36	0.07	54.50	0.09	None
	ABDOMINAL PAIN			0.14	0.42	-0.68	0.97	1.15	0.51	2.63	0.73						
	**OPIOID**	MV Egger	38	0.25	0.21	-0.16	0.66	1.29	0.85	1.94	0.24	49.11	35	0.06			
	ABDOMINAL PAIN			0.14	0.45	-0.74	1.02	1.15	0.48	2.77	0.76						
	Intercept			0.00	0.01	-0.02	0.02	1.00	0.98	1.02	0.97						

[a] For outcome phenotype MDD, summary statistics on MV instruments extracted from PGC GWAS excluding both UK Biobank (N = 29740) and 23andMe cohorts for total N = 143265 (no sample overlap)	
**Abbreviations:**	
MVMR	Multivariable Mendelian randomization
UKB	UK Biobank
N SNPs	Number of single nucleotide polymorphisms in instrument
beta	effect estimate
se	standard error of effect estimate
LCI	lower bound of 95% confidence interval of estimate
UCI	upper bound of 95% confidence interval of estimate
OR	odds ratio for binary outcome
OR LCI	lower bound of 95% confidence interval of odds ratio estimate
OR UCI	upper bound of 95% confidence interval of odds ratio estimate
P-value	P-value of effect estimate
**Heterogeneity Tests:**	
Q	Q statistic
Q df	Q statistic degrees of freedom
Q P-value	Q statistic P-value
**MR PRESSO test:**	
RSS obs	residual sums squares of observations
P-value	P-value of global test

© 2020 Rosoff DB et al. *JAMA Psychiatry*.

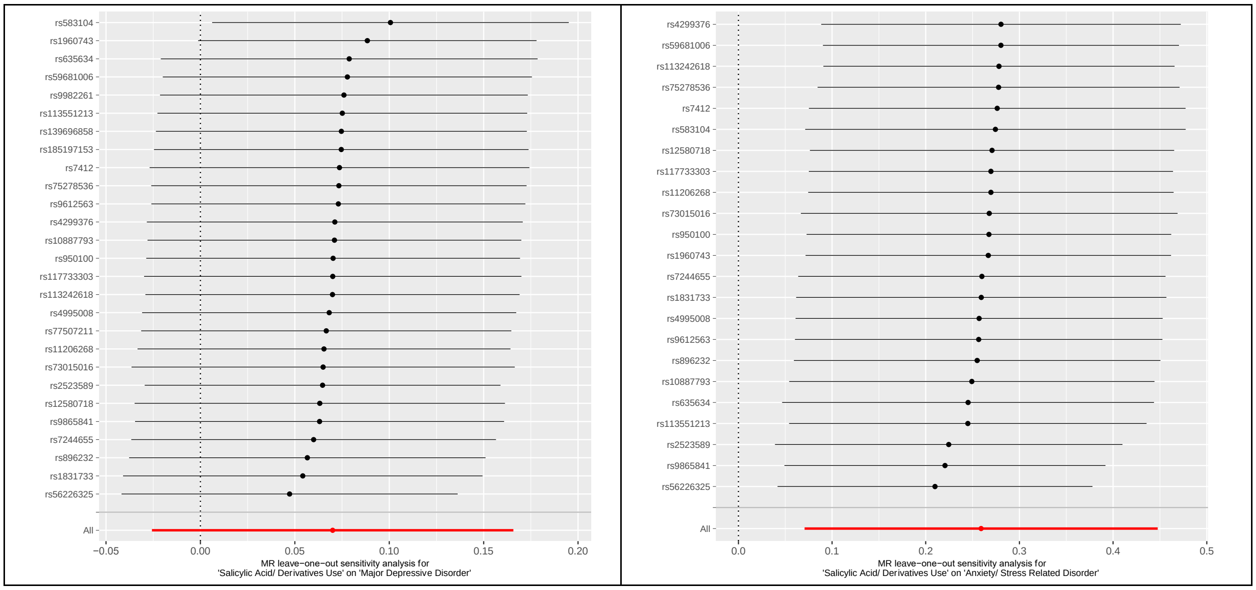

eFigure 1. Leave-1-Out Analysis of Associations of Genetic Risk of Salicylic Acid/ Derivatives Use on Risk of MDD and ASRD. IVW performed leaving out each SNP in turn to identify if a single SNP may be driving the association, with results illustrated in plot showing that no SNP was driving the association between genetic risk of prescription opioid use and MDD (LEFT) and ASRD (RIGHT). **Abbreviations**: MDD, major depression or major depressive disorder (depending on study used in GWAS); ASRD, anxiety and stress-related disorders; IVW, inverse-variance weighted Mendelian randomization; SNP, single nucleotide polymorphism.

© 2020 Rosoff DB et al. *JAMA Psychiatry*.

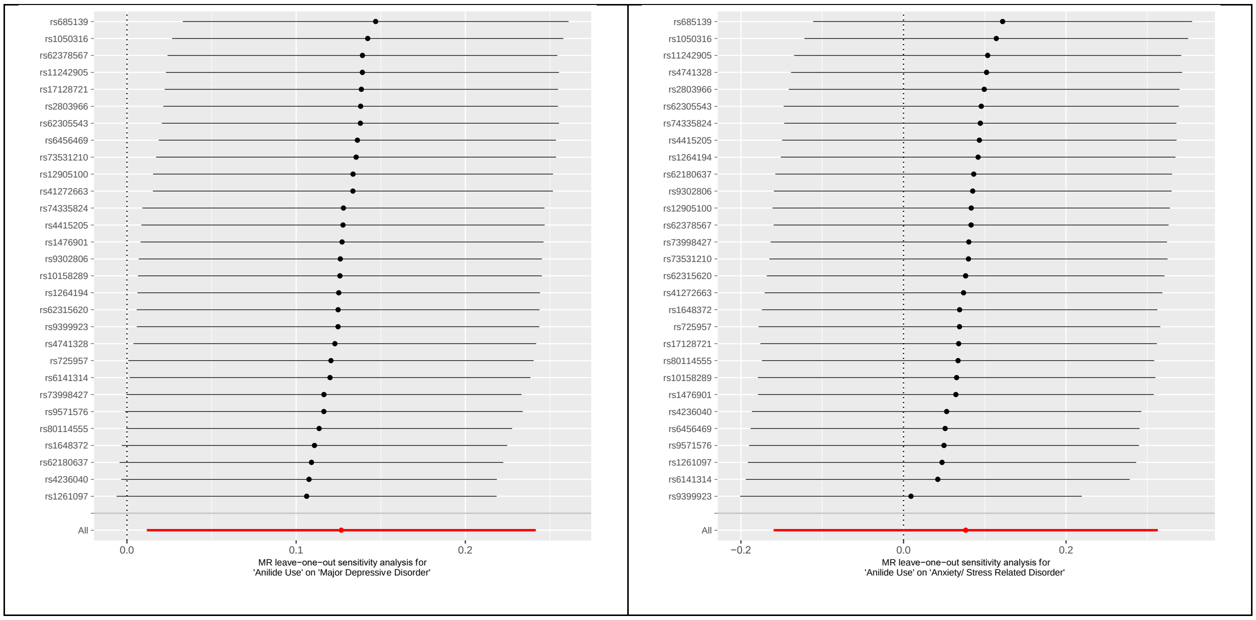

eFigure 2. Leave-1-Out Analysis of Associations of Genetic Risk of Anilide Use on Risk of MDD and ASRD. IVW performed leaving out each SNP in turn to identify if a single SNP may be driving the association, with results illustrated in plot showing that no SNP was driving the association between genetic risk of prescription opioid use and MDD (LEFT) and ASRD (RIGHT). **Abbreviations:** MDD, major depression or major depressive disorder (depending on study used in GWAS); ASRD, anxiety and stress-related disorders; IVW, inverse-variance weighted Mendelian randomization; SNP, single nucleotide polymorphism.

© 2020 Rosoff DB et al. *JAMA Psychiatry.*

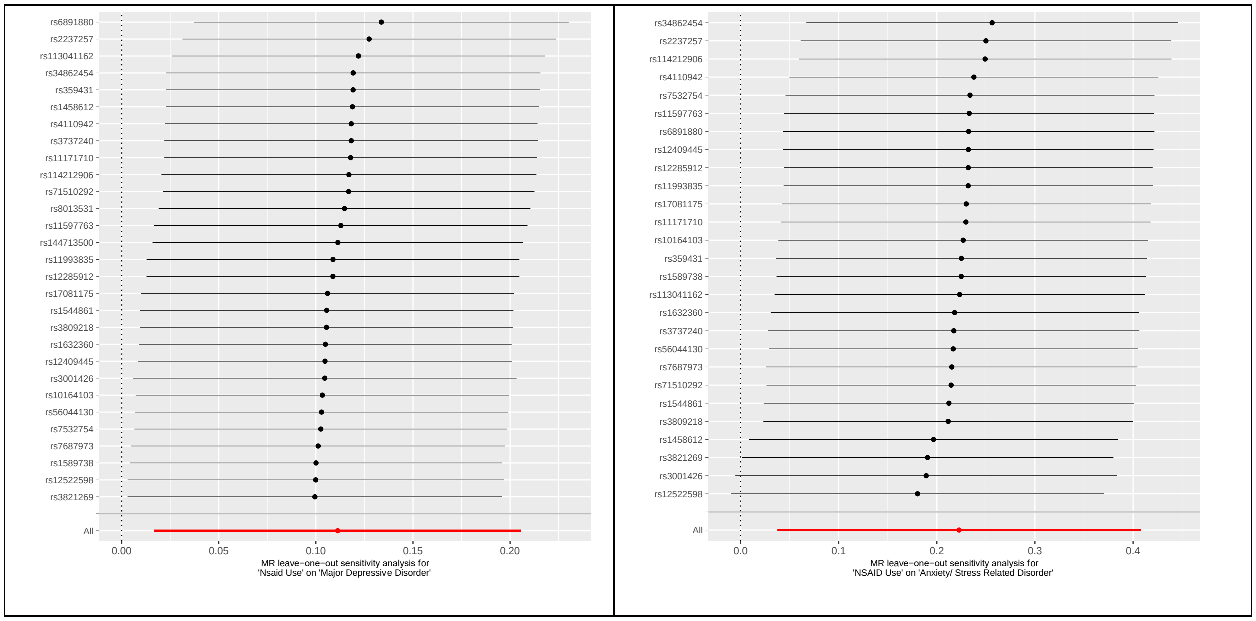

eFigure 3. Leave-1-Out Analysis of Associations of Genetic Risk of NSAID Use on Risk of MDD and ASRD. IVW performed leaving out each SNP in turn to identify if a single SNP may be driving the association, with results illustrated in plot showing that no SNP was driving the association between genetic risk of prescription opioid use and MDD (LEFT) and ASRD (RIGHT). **Abbreviations:** MDD, major depression or major depressive disorder (depending on study used in GWAS); ASRD, anxiety and stress-related disorders; IVW, inverse-variance weighted Mendelian randomization; SNP, single nucleotide polymorphism; NSAID, nonsteroidal anti-inflammatory drugs.

© 2020 Rosoff DB et al. *JAMA Psychiatry*.

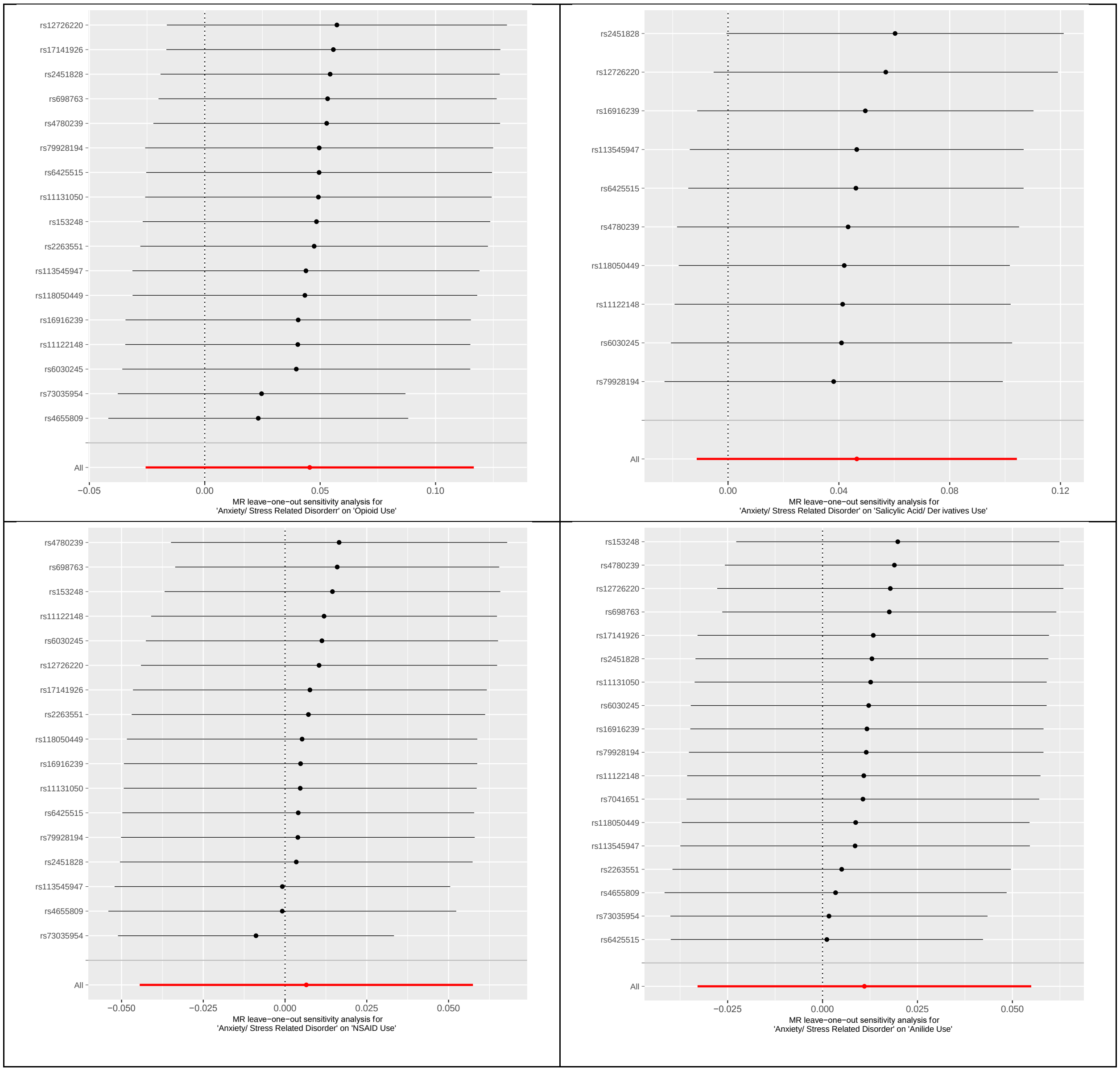

eFigure 4. Leave-1-Out Analysis of Associations of Genetic Risk of ASRD on Risk of Pain Medications Use. IVW performed leaving out each SNP in turn to identify if a single SNP may be driving the association, with results illustrated in plot showing that no SNP was driving the association between genetic risk of ASRD and Prescription Opioid Use (TOP LEFT), Salicylic Acid/ Derivatives Use (TOP RIGHT), NSAID Use (BOTTOM LEFT) and Anilde Use (BOTTOM RIGHT).
Abbreviations: MDD, major depression or major depressive disorder (depending on study used in GWAS); ASRD, anxiety and stress-related disorders; IVW, inverse-variance weighted Mendelian randomization; SNP, single nucleotide polymorphism; NSAID, nonsteroidal anti-inflammatory drugs.

© 2020 Rosoff DB et al. *JAMA Psychiatry*.

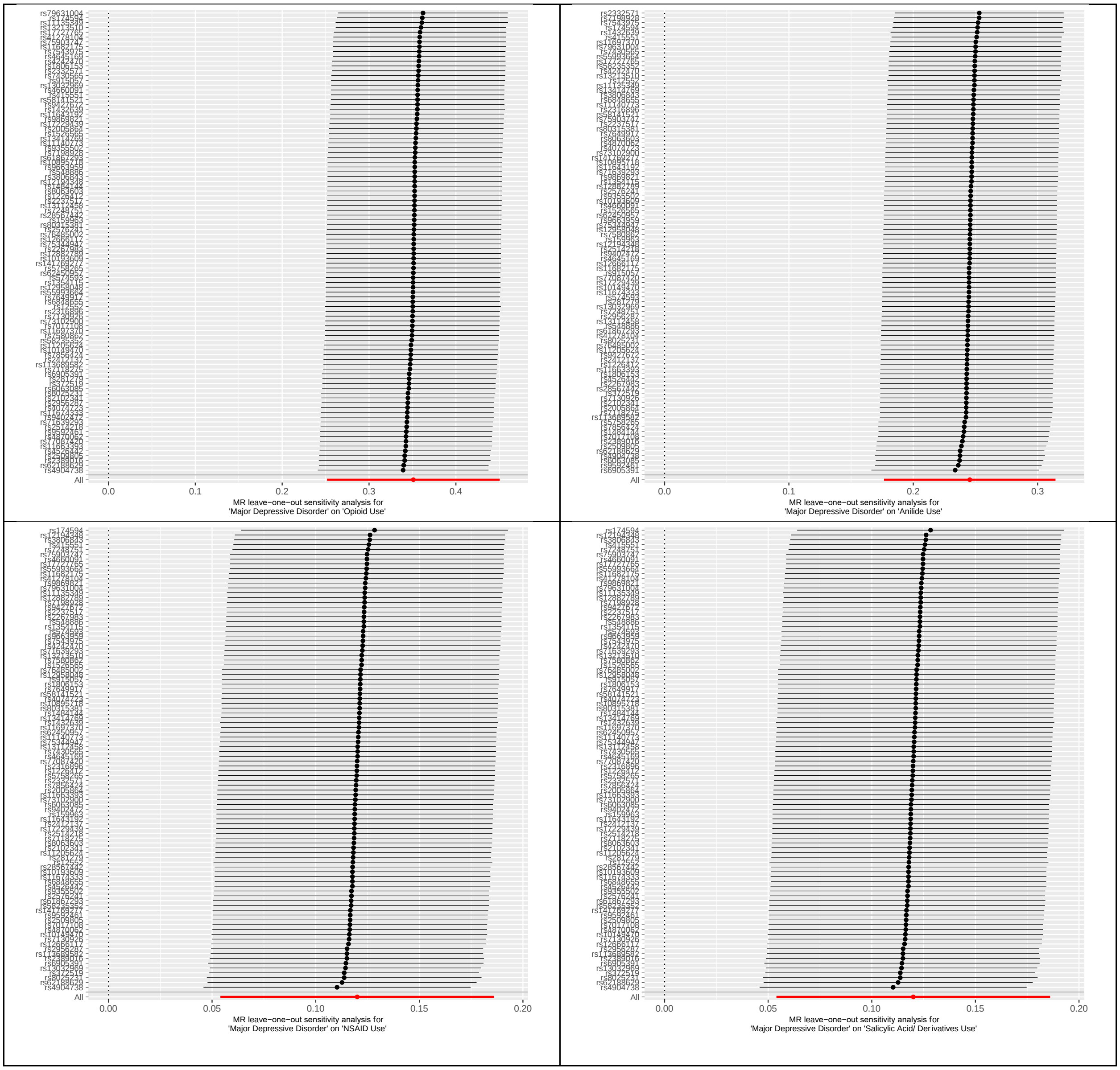

eFigure 5. Leave-1-Out Analysis of Associations of Genetic Risk of MDD on Risk of Pain Medications Use. IVW performed leaving out each SNP in turn to identify if a single SNP may be driving the association, with results illustrated in plot showing that no SNP was driving the association between genetic risk of ASRD and Prescription Opioid Use (TOP LEFT), Salicylic Acid/ Derivatives Use (TOP RIGHT), NSAID Use (BOTTOM LEFT) and Anilde Use (BOTTOM RIGHT).
Abbreviations: MDD, major depression or major depressive disorder (depending on study used in GWAS); ASRD, anxiety and stress-related disorders; IVW, inverse-variance weighted Mendelian randomization; SNP, single nucleotide polymorphism; NSAID, nonsteroidal anti-inflammatory drugs.

© 2020 Rosoff DB et al. *JAMA Psychiatry.*
